# Intratumoral delivery of 4-1BBL boosts IL-12-triggered anti-glioma immunity

**DOI:** 10.1016/j.ymthe.2025.08.028

**Published:** 2025-08-20

**Authors:** Taral R. Lunavat, Lisa Nieland, Sanne M. van de Looij, Alexandra J.E.M. de Reus, Charles P. Couturier, Chadi A. El Farran, Tyler E. Miller, Julia K. Lill, Maryam Schübel, Tianhe Xiao, Emilio Di Ianni, Elliot C. Woods, Yi Sun, David Rufino-Ramos, Thomas S. van Solinge, Shadi Mahjoum, Emily Grandell, Mao Li, Vamsi Mangena, Gavin P. Dunn, Russell W. Jenkins, Thorsten R. Mempel, Xandra O. Breakefield, Koen Breyne

**Affiliations:** 1 Molecular Neurogenetics Unit, Department of Neurology, Massachusetts General Hospital and Harvard Medical School, Boston, MA 02129, USA; 2 Department of Biomedicine, University of Bergen, 5019 Bergen, Norway; 3 Department of Neurosurgery, Leiden University Medical Center, 2300 RC Leiden, the Netherlands; 4 Broad Institute of MIT and Harvard, Cambridge, MA 02142, USA; 5 Department of Cancer Biology, Dana-Farber Cancer Institute, Boston, MA 02215, USA; 6 Institute of Medical Engineering and Sciences and Department of Chemistry, Massachusetts Institute Technology, Cambridge, MA 02139, USA; 7 Koch Institute for Integrative Cancer Research, Massachusetts Institutes of Technology, Cambridge, MA 02139, USA; 8 Department of Neurosurgery, Brigham and Women’s Hospital, Boston, MA 02115, USA; 9 Department of Neurology and Neurosurgery, Montreal Neurological Institute, McGill University, Montreal, QC, Canada; 10 Department of Human Genetics, McGill University, Montreal, QC, Canada; 11 Department of Cell Biology and Pathology, Harvard Medical School, Boston, MA 02215, USA; 12 Ludwig Center at Harvard Medical School, Boston, MA 02215, USA; 13 Department of Pathology and Center for Cancer Research, Massachusetts General Hospital and Harvard Medical School, Boston, MA 02114, USA; 14 Center for Immunology and Inflammatory Diseases, Massachusetts General Hospital and Harvard Medical School, Boston, MA 02129, USA; 15 Mass General Cancer Center, Krantz Family Center for Cancer Research, Department of Medicine, Massachusetts General Hospital, Harvard Medical School, Boston, MA 02114, USA; 16 Center for Genomic Medicine, Massachusetts General Hospital, Boston, MA 02114, USA; 17 Department of Pathology, Massachusetts General Hospital, Boston, MA 02114, USA; 18 Department of Pathology, Harvard Medical School, Boston, MA 02115, USA; 19 Department of Neurosurgery, Massachusetts General Hospital, Harvard Medical School, Boston, MA 02114, USA; 20 Brain Tumor Immunology and Immunotherapy Program, Department of Neurosurgery, Massachusetts General Hospital, Harvard Medical School, Boston, MA 02114, USA; 21 Harvard-MIT Health Sciences and Technology, Cambridge, MA 02139, USA

**Keywords:** glioblastoma, cytotoxic CD8 T cells, IL-12, immunotherapy, 4-1BBL, AAV vector, gene therapy

## Abstract

The standard of care in high-grade gliomas has remained unchanged in the past 20 years. Efforts to replicate effective immunotherapies in non-cranial tumors have led to only modest therapeutical improvements for patients with glioma. Here, we demonstrate that intratumoral (i.t.) administration of recombinant interleukin-12 (rIL-12) promotes local cytotoxic CD8^POS^ T cell accumulation and conversion into an effector-like state, resulting in a dose-dependent survival benefit in preclinical glioblastoma (GB) mouse models. This tumor-reactive CD8 T cell response is further supported by intratumoral rIL-12-sensing dendritic cells (DCs) and is accompanied by the co-stimulatory receptor 4-1BB expression in both cell types. Given that DCs and CD8^POS^ T cells are functionally suppressed in the tumor microenvironments (TME) of *de novo* and recurrent glioma patients, we tested whether anti-tumor response at the rIL-12-inflamed tumor site could be enhanced with 4-1BBL, the ligand of 4-1BB. 4-1BBL was delivered using an adeno-associated virus (AAV) vector targeting GFAP-expressing cells and resulted in prolonged survival of rIL-1 2-treated GB-bearing mice. This study establishes that tumor antigen (Ag)-specific CD8 T cell activity can be augmented by incorporating an AAV-vector-mediated gene therapy approach, effectively enhancing anti-GB immunity in the TME.

## Graphical abstract

**Figure:**
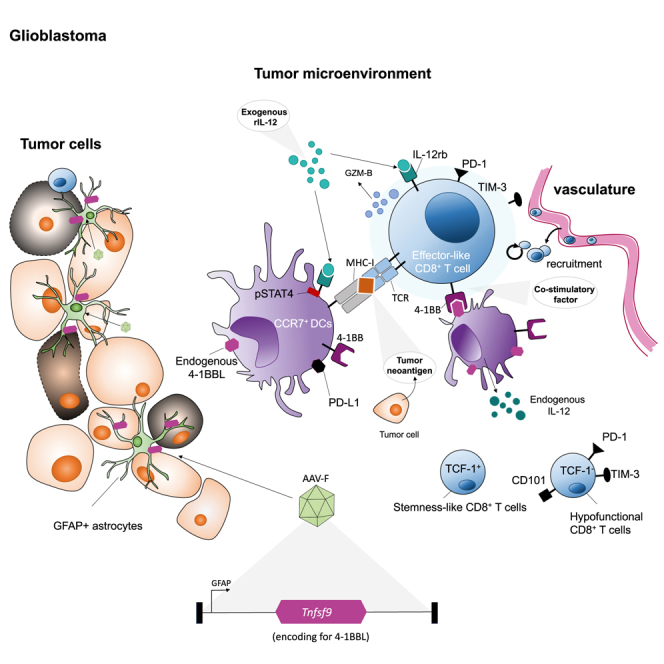

## Teaser

Glioblastoma is the most malignant cancer of the brain with an incidence of 2–5 cases per 100,000 people per year in the US. Breyne and colleagues utilize an immune-gene combination therapy, activating CTLs at the tumor border, showing prolonged survival in preclinical mouse models.

## Introduction

Glioblastoma (GB) isocitrate dehydrogenase (IDH) wild type (WT), a high-grade glioma, is the most lethal primary cancer in the central nervous system (CNS),^1^^,^^2^ with a median survival of 14.7 months after initial diagnosis.^3^^,^^4^ The current standard of care treatment paradigm includes surgical tumor resection, followed by radiotherapy and temozolomide.^5^ Although the complexity of GB immunology is still being uncovered, GB is generally considered a “cold” tumor typically marked by minimal expression of neoantigens and the presence of various immune checkpoints and immune-inhibitory cytokines that augment the immunosuppressive nature of this cancer.^6^^,^^7^ Even when an anti-tumor immune response develops, it is suppressed not only by tumor cells but also by an immune suppressed tumor microenvironment (TME).^8^^,^^9^

Numerous clinical trials have aimed to invigorate anti-tumor immunity through targeting immune checkpoint inhibitors (ICI) which target programmed cell death protein-1 (PD-1) (nivolumab and pembrolizumab), PD-L1 (atezolizumab and durvalumab), or T lymphocyte-associated antigen 4 (CTLA-4) (ipilimumab).^10^^,^^11^^,^^12^^,^^13^^,^^14^^,^^15^^,^^16^ Unfortunately, these ICI strategies have not shown therapeutic efficacy in GB patients.^17^^,^^18^ More recently, preclinical studies have utilized adeno-associated virus (AAV) vectors to promote anti-tumor immunity. For example, AAV-LIGHT vectors targeting endothelial cells in the tumor vasculature and expressing the lymphocyte recruiting cytokine LIGHT, induced CD8 T cell infiltration that prolonged survival in murine GB.^19^ AAV6 transduction of astrocytes to express chemokine CXCL9 increased tumor infiltration of cytotoxic lymphocytes when combined with anti-PD-1 immune checkpoint blockade.^20^ Combination therapies are clearly needed to activate multiple immune components.

Although GB remains refractory to immunotherapy, encouraging developments suggest that local administration of IL-12, a pro-inflammatory cytokine, can invigorate the immune system in recurrent glioma patients.^21^ The therapeutic effect of IL-12 was enhanced by injecting a replication-incompetent adenovirus vector encoding a drug-inducible IL-12 directly into the tumor resection site. IL-12 expression was activated following repeated, oral administration of 20 mg of the blood-brain barrier-permeable drug, veledimex (VDX).^22^^,^^23^ This gene therapy extended the median survival to 17.8 months in recurrent glioma patients without dexamethasone treatment, compared with a median survival of 8.14 months in historical controls. Despite this therapy, patients still progressed over time, and a local increase in PD-L1 was observed.^21^ To counteract this induced immune suppression, a combined therapy of IL-12 and the ICI nivolumab was explored but did not lead to extended survival in phase 2 clinical trials.^24^ Instead of focusing on blocking the immunosuppressive signaling by tumor cells and the TME,^21^^,^^25^ efforts could be redirected toward enhancing the activity of anti-tumor cells associated with GB.

In GB patients, CD8 T cells are typically present in low numbers, representing only 0.6% of primary-derived tumor tissue,^26^^,^^27^^,^^28^ and they have a heterogeneous phenotype.^29^ The failure of immunotherapy for GB is partly attributed to the suppression of both the accumulation and anti-tumor functions of CD8 T cells.^7^ In patients with malignancies, CD8 T cells can become dysfunctional with compromised cytotoxic functions.^30^^,^^31^ Some anti-tumor T cell responses require direct instructions from dendritic cells (DCs), that (cross-)present tumor-derived antigens (Ags) on their major histocompatibility complex (MHC).^32^ However, in GB, DCs themselves often become suppressed.^30^ Therefore, it is necessary to not only stimulate T cells directly but also activate DCs in the GB TME to promote the T cell activity needed for tumor control.

Here, we demonstrate that local administration of recombinant interleukin-12 (rIL-12) gives rise to intratumoral (i.t.) effector-like CD8^POS^ T cell response involved in GB regression. In addition to the direct stimulation of CD8^POS^ T cells by rIL-12, specialized DCs are recruited and activated at the tumor site, further enhancing CD8^POS^ T cell activity. To support both the function of CD8^POS^ T cells and DCs during the rIL-12 inflammatory response, we established a reservoir of 4-1BBL at the tumor. We selected this co-stimulatory molecule because both CD8^POS^ T cells and DCs express its receptor, 4-1BB. An AAV vector was used to express 4-1BBL, mainly in reactive astrocytes within the TME, thereby enhancing the rIL-12-driven survival benefit. Our findings were predominantly tested using a syngeneic mouse model with intracranially (i.c.) engrafted CT-2A GB cells and further validated with GL261 and 005 GBs.

## Results

Throughout the article, we use *TNFRSF9/TNFSF9* and *Tnfrsf9/Tnfsf9* to refer to gene and transcriptomic data and 4-1BB/4-1BBL to describe protein-based analyses (summarized in Table S1), in accordance with HGNC guidelines for human and MGI guidelines for mouse genes and proteins.

### Intratumorally administered rIL-12 prolongs survival of GB-bearing mice

**Figure 1:**
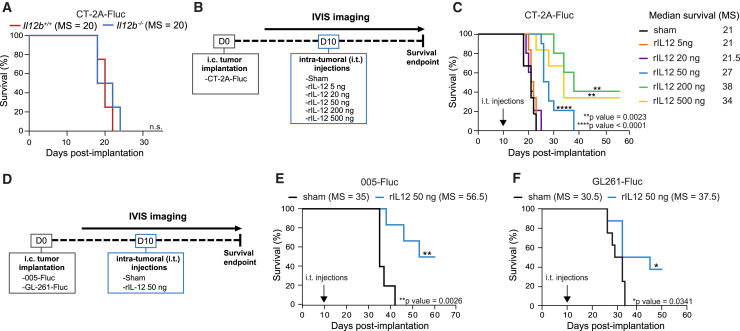
Intratumorally administered rIL-12 post-tumor engraftment prolongs survival of GB-bearing mice (A) Survival curves of GB-bearing mice in Il12b^+/+^ and Il12b^−/−^ mice*.* Kaplan-Meier survival curves showing no overall survival benefit of CT-2A tumor-bearing *Il12*^*+/+*^ mice (red) compared with *Il12*^−/−^ mice (blue) (*n* = 5 mice per group, 100,000 cells per mouse) (median survival [MS] of 20 and 18 days, respectively). Data represent at least two independent experiments. No differences were observed between the groups based on MS. Log rank (Mantel-Cox) test, not significant (n.s.). Median survival in days (MS). (B) Schematic illustration of the *in vivo* experimental setup*.* CT-2A-FLuc GB (*n* = 100,000) cells were injected i.c. into the left striatum on day 0. Starting on day 7, tumor growth was monitored every 3–4 days by IVIS an *in vivo* bioluminescent imaging system. Based on FLuc levels on days 7 and 10, mice with a similar tumor size were allocated to sham (50 ng of Fc control) or rIL-12 conjugated to Fc-treatment groups (ranging between 5 and 500 ng) on day 10 (Figure S2B). Sham and rIL-12 solutions were administered intratumorally (i.t.) on day 10. (C) Survival of CT-2A tumor-bearing mice with rIL-12 treatment. Kaplan-Meier survival curves show an rIL-12 dose-response study in mice implanted with (CT-2A-Fluc, 100,000 cells per mouse) GB cells, compared with sham treatment. The arrow indicates the time point of i.t. injections of sham or rIL-12. The median survival was significantly increased when GB mice were treated with 50, 200, or 500 ng (27, 38, and 34 days, respectively; ∗∗*p* = 0.0023, ∗∗∗∗*p* < 0.0001) compared with sham control 20 days. Median survival for 5 and 20 ng rIL-12 were 22.5 and 21.5 days, respectively (50 ng sham, *n* = 6; 5 ng rIL-12, *n* = 6; 20 ng rIL-12, *n* = 5; 50 ng rIL-12, *n* = 10; 200 ng rIL-12, *n* = 5, and 500 ng rIL-12, *n* = 6). Data represent three independent experiments and were analyzed using the log rank (Mantel-Cox) test. Median survival in days (MS). (D) Schematic illustration of the *in vivo* experimental setup*.* 005-FLuc and GL261-FLuc GB cells (*n* = 100,000) were injected i.c. into the left striatum on day 0. Starting on day 7, tumor growth was monitored every 3–4 days by IVIS BLI. Based on FLuc levels on days 7 and 10, mice with similar tumor sizes were allocated to 50 ng sham or 50 ng rIL-12 treatment groups on day 10. Sham and rIL-12 were administered i.t. (E) Survival of rIL-12-treated 005-FLuc-bearing mice*.* Kaplan-Meier curves displaying the percentage of survival of 005-FLuc-bearing mice (100,000 cells at the time of injection) with treatment on day 10 post-tumor injection, comparing i.t. injection of 50 ng rIL-12 (blue) to sham control (black) (*n* = 5–6 mice per group). Approximately ∼50% of the rIL-12-treated mice stayed healthy over 50 days (∗∗*p* = 0.0026). Data represent at least two independent experiments. Data were analyzed using log rank (Mantel-Cox) test, ∗∗*p* < 0.01. Median survival in days (MS). (F) Survival of rIL-12-treated GL261-FLuc-bearing mice*.* Kaplan-Meier curves displaying the percentage of survival of GL261-bearing mice (100,000 cells at the time of injection) with treatment on day 10 post-tumor injection comparing i.t. injection of 50 ng rIL-12 (blue) with sham control (Fc-black) (*n* = 5–6 mice per group). Approximately ∼40% of the rIL-12-treated mice stayed healthy over 50 days (∗*p* = 0.0341). Data represent at least two independent experiments. Data were analyzed using log rank (Mantel-Cox) test, ∗*p* < 0.05. Median survival in days (MS).

IL-12 is a pro-inflammatory cytokine composed of two subunits, IL-12A (p35) and IL-12B (p40), which are covalently linked to form a bioactive IL-12p70 heterodimer complex.^31^ At the tumor site, IL-12 can promote anti-tumor immunity^33^^,^^34^ by altering the cellular composition of the TME.^35^ In the context of primary human glioma, high or low expression levels of *IL12A*/*B* in the tumor did not predict an overall survival benefit of GB patients (Figure S1A). Single-cell RNA sequencing (scRNA-seq) analysis of human gliomas (Table S2), encompassing WHO grades II, III, and IV gliomas as well as both IDH 1 and 2 mutant and GB IDH-WT,^2^ suggests that the limited predictive value of *IL12A/B*-associated survival may be due to its low expression across human gliomas (Figure S1B). The transcript levels of *IL12A*/*B* in recurrent glioma were comparable with *de novo* tumors (Figure S1C) and independent of glioma grade (Figure S1D). By processing multiple scRNA-seq datasets across three murine GB cell lines (CT-2A, GL261, and 005) (Table S2) with different genetic and phenotypic profiles (Table S3) and analyzing GB tumor cells, we confirmed a trend of low *Il12a/b* gene expression in the TME and the tumor cells (Figures S1E and S1F). The low role of host-derived IL-12 in GB is illustrated by similar median survival of 20 days post-i.c. implantation of CT-2A cells, in *Il12b*^−/−^ mice^36^ compared with *Il12b*^*+/+*^mice (Figure 1A). We further quantified IL-12p70 protein levels in the ipsilateral (tumor-implanted) hemispheres in *Il12b*^*+/+*^ and *Il12b*^−/−^ mice (Figure S1G) and found that IL-12 was expressed at low levels by both genotypes (*Il-12b*^*+/+*^ 1.2 ± 0.34 fg and *Il-12b*^−/−^ 0.8 ± 0.03 fg, mean ± SEM (standard error of the mean)), indicating that CT-2A tumor growth did not increase in rIL-12 expression in the ipsilateral compared with the contralateral hemisphere.

To increase IL-12 at the tumor site to therapeutically effective levels, we injected different doses (5, 20, 50 200, or 500 ng) of murine rIL-12 conjugated to Fc (hereafter referred to as rIL-12) i.t. 10 days after engraftment of CT-2A-Firefly Luciferase (FLuc) cells (Figure 1B) and compared survival outcomes to mice i.t. injected with sham (Fc without the IL-12 fusion). FLuc was introduced into CT-2A cells using a lentiviral vector (LVV), enabling *in vivo* bioluminescence imaging (BLI) to monitor tumor growth in the mouse brain (Figure S2A). On day 7, 3 days prior to i.t. rIL-12/sham (sham represents i.t. injection of 50 ng of Fc) injections, tumors had similar sizes across groups based on BLI (Figure S2B), while after day 10 of treatment the BLI signals were different depending on the rIL-12 dose (Figure S2C). Together with the evaluation of body weight (Figure S2D) and survival (Figure 1C), BLI measurements revealed different treatment responses in the GB-bearing mice over time (Figure S2D). Some GB-bearing mice did not respond to treatment (non-responders), while others showed reduced tumor size but eventually died from the tumor (treatment responders). A third group of treated mice survived the GB implantation (treatment survivors). Among the treated non-responders, mice exhibited similar outcomes to the sham group, characterized by a steady increase in tumor size and a decline in body weight, indicative of poor health. This response pattern included all GB-bearing mice treated with 5 and 20 ng rIL-12. Notably, a cohort of mice, specifically 64% and 33% of the GB-bearing mice treated with 50 and 500 ng of rIL-12, respectively, exhibited a similar response as the sham-treated GB-bearing mice. A slower increase in the BLI signal and a minimal decrease in body mass were observed in the treatment responders compared with the non-responders. The proportion of treatment responders was 36%, 60%, and 33% among the GB-bearing mice treated with 50, 200, and 500 ng of rIL-12, respectively. The treatment survivors demonstrated favorable outcomes with rIL-12 treatment; 40% and 33% of GB-bearing mice treated with 200 and 500 ng rIL-12, respectively, displayed tumor regression concomitant with stable body weight and lived for at least 60 days without apparent health concerns.

The varying responses to different doses of rIL-12 resulted in different survival outcomes. Mice treated with 50 ng rIL-12 showed 6 days of improved median survival compared with sham-treated mice. Mice treated with 200 and 500 ng benefited 17 and 13 days, respectively (Figure 1C). Pathology evaluation of tumor-implanted mice was performed using hematoxylin and eosin (H&E) staining, and tumor sizes were quantified comparing the tested rIL-12 dosages (Figures S2E and S2F). The i.t. administration of 50 ng rIL-12 was considered the optimal dose for subsequent experiments, as while this dose significantly increased median survival, it was not sufficient to cure tumor-implanted mice. This closely reflected survival outcomes seen in IDH1/2-WT recurrent glioma patients treated i.t. with adenovirus vector gene therapy delivering IL-12.^24^ Additionally, the 50 ng rIL-12 dosage was chosen because it minimizes the risk of rIL-12-associated toxicity and allows for complementary therapies to further enhance the rIL-12-driven survival effect. The systemic lack of toxicity of the 50 ng rIL-12 treatment was demonstrated by comprehensive blood chemistry analysis in mice (Figure S2G). No significant elevation in liver biomarkers—including albumin, alkaline phosphatase (ALP), alanine transaminase (ALT), calcium, cholesterol, creatinine, blood urea nitrogen, globulin, glucose, and phosphorus—was detected after i.t. injection of rIL-12 (50 ng) compared with sham. The blood tests we conducted indicated no systemic toxicity upon localized administration of rIL-12, in contrast to the previously reported toxic effects of systemic rIL-12 administration.^37^

Next, we validated the efficacy of the rIL-12 therapy with the 005 and GL261 cell lines, the former known for its diffuse tumor growth, similar to human glioma^38^ (Figure 1D). I.t. rIL-12 treatment (50 ng) administered 10 days post-tumor cell implantation was effective in 005-FLuc GB-bearing mice treated with rIL-12 with a 21.5-day improved median survival compared with sham. Importantly, 50% of the mice survived for over 50 days (Figures 1E and S2H). In the GL261 model, the median survival was 37.5 days in the rIL-12-treated mice compared with 30.5 days in sham-treated mice. Approximately 40% of rIL-12-treated GL261-FLuc tumor-bearing mice survived for over 50 days (Figures 1F and S2I).

Overall, the survival outcomes indicate that i.t. administered rIL-12 supplemented the low levels of endogenous IL-12 in GB and achieved therapeutic effects with 50 ng dosage in multiple syngeneic mouse GB models.

### Identifying the cell types within the GB TME that respond to rIL-12

**Figure 2:**
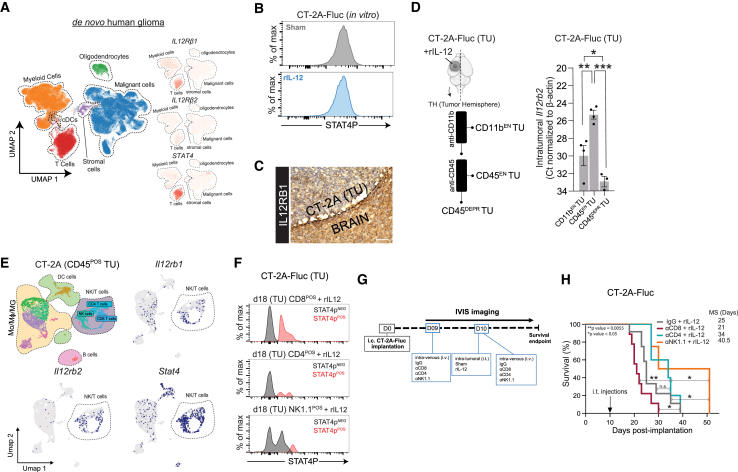
Identifying the cell types within the GB TME that can trigger a rIL-12-mediated anti-tumor response (A) *IL12Rβ1*, *IL12Rβ2*, and *STAT4* expression in immune cells of GB patients*.* Distinct cell types were clustered, annotated, and visualized with a high-resolution color-coded UMAP projection. To visualize *IL12Rβ1*, *IL12Rβ2*, and *STAT4* expression, dot plots were used. No expression was observed in the tumor compartment, but it was detected in the immune cell compartment. (B) Representative flow cytometry plots of STAT4p levels in CT-2A-FLuc-cultured cells*.* CT-2A-FLuc cells were exposed to sham or rIL-12 for 24 h, STAT4p expression was measured by flow cytometry and no differences were observed (dataset from Miller et al.^50^). (C) Positive *IL12RB1* receptor staining in brain tissues implanted with CT-2A cells*.* Immunohistochemistry of IL12RB1-positive cells (brown) in the TME of the CT-2A tumor (TU) cells (blue) (magnification 20×). Scale bar, 100 μm. (D) Decoupling non-immune cells and Mo/Mϕ/MG cells from other immune cells in GB-bearing mouse brains to explore Il-12R expression*.* A schematic display shows the sequential method used to isolate CD11b-enriched (EN) tumor (TU) cells, CD45^EN^ TU cells, and CD45-deprived (DEPR) TU cells derived from the tumor hemisphere (TH) of mouse brains post-rIL-12 treatment (left). I.t. *Il12rb2* expression was analyzed 8 days after rIL-12 treatement. *Il12rb2* was expressed significantly higher in CD45^EN^ TU compared with CD11b^EN^ TU (*p* = 0.0043) and CD45^DEPR^ TU cells (*p* = 0.0001). CD45^EN^ TU *Il12rb2* levels were significantly higher (*p* = 0.0136) than CD45^DEPR^ TU cells. Data represent CT values normalized to β-actin. Data represent three independent experiments and are presented as the mean ± SEM (error bars). Data were analyzed using one-way ANOVA, ∗*p* < 0.05, ∗∗*p* < 0.01, ∗∗∗*p* < 0.001 (right). (E) Expression of *Il12rb1*, *Il12rb2*, and *STAT4p* in immune cell populations of TME of mouse GB models*.* scRNA-seq datasets of CD45^POS^-sorted tumor cells derived from mouse GB tumor (TU) (CT-2A, *n* = 3) were analyzed (dataset from Tomaszewski et al.^45^). Distinct cell types were clustered, annotated, and visualized with a high-resolution color-coded UMAP projection. To visualize *Il12rb1*, *Il12rb2*, and *STAT4p* expression in different datasets, feature plots were used to display the expression in NK/T cluster (marked in dotted lines) (datasets from Pombo Antunes et al.^45^ Tomaszewski et al.^46^ and Chen et al.^45^^,^^46^^,^^119^). (F) *STAT4p* levels in CD8 T cell, CD4 T cell, and NK cell populations post-rIL-12 treatment*.* CD8 T cells, CD4 T cells, and NK1.1 cells were isolated from CT-2A-FLuc tumor (TU)-bearing, rIL-12-treated mice on day 18 post-tumor implantation. Representative flow plots showed STAT4p^POS^ and STAT4p^NEG^ levels as the percentage of max. All three cell types express STAT4p. (G) Schematic illustration of the T cell depletion strategy in rIL-12-treated GB mouse*.* On day 0, 100,000 GB cells (CT-2A-FLuc) were implanted i.c. into the left striatum. Anti-CD8 or IgG control was injected i.v. on day 9 (50 μg, retro-orbitally). On day 10, mice were injected with 50 ng rIL-12 or sham control i.t. at the tumor site and anti-CD8 or IgG control was injected i.v. (100 μg, retro-orbitally) to deplete endogenous CD8^POS^ T cells systemically. (H) Importance of CD8^POS^ T cell recruitment for survival benefit in anti-GB therapy with rIL-12*.* Kaplan-Meier curves showing survival outcome of tumor-bearing mice injected with IgG and rIL-12 (gray), with CD8 depletion (anti-CD8) and rIL-12 (red), with CD4 depletion (anti-CD4) and rIL-12 (turquoise) and with NK cells depletion (anti-NK1.1) and rIL-12 (orange) (*n* = 6–8 mice per group). IgG control treated with rIL-12 had a median survival (MS) of 25 days, whereas anti-CD8 had a median survival of 21 days, anti-CD4 34 days, and anti-NK 40.5 days. IgG control did not differ compared with anti-CD4 but had a significantly improved survival compared with anti-CD8 (*p* = 0.0372), and anti-NK (*p* = 0.0479). Anti-CD8 had significant improved median survival compared with anti-NK (*p* = 0.0122) and anti-CD4 (*p* = 0.0055). Data represent two independent experiments and were analyzed using log rank (Mantel-Cox) test, ∗*p* < 0.05; ∗∗*p* < 0.001. Median survival in days (MS).

IL-12p70 binds to the dimeric receptor composed of the IL-12 receptor β1 (IL12Rβ1) and β2 (IL12Rβ2) subunits, leading to phosphorylation of Tyr693 on the receptor-associated STAT4 transcription factor.^39^ This phosphorylation promotes STAT4 dimerization, thereby initiating pro-inflammatory signaling.^31^^,^^40^ To identify the cells capable of an IL-12p70-mediated anti-GB effect, we analyzed available human glioma scRNA-seq datasets (Table S2) for the expression of relevant genes (*IL12Rβ1*, *Il12Rβ2*, and *STAT4*) (Figure 2A). Immune cells expressed all three markers in contrast to malignant (tumor) cells, oligodendrocytes, and stromal (vascular) cells, which expressed low-to-no levels. Markers were predominantly co-expressed in tumor-associated CD4 T cells, CD8 T cells, and natural killer cells (annotated as the NK/T cells cluster); however, in datasets with broader immune representation (as these datasets were enriched with CD45^POS^ cells), expression was also observed in macrophages, monocytes, microglia, and DCs in the TME of *de novo* and recurrent glioma (Figure S3A). We also explored if rIL-12 had a direct effect on tumor cells. *Il12rb1* was expressed only at low levels and *Il12rb*2 was not detected in the murine tumor cells *in vitro* (Figure S3B). This explains why, upon *in vitro* exposure to 50 ng rIL-12 or sham, GB cell proliferation was not affected over the course of 5 days (Figure S3C), nor did it alter the Tyr693 STAT-4 phosphorylation (STAT4p) levels (Figures 2B and S3D). This observation was confirmed in brain sections of tumor-bearing mice, where IL12Rβ1 expression was minimally present within the CT-2A tumor and predominantly localized at the tumor periphery (Figure 2C) compared with the negative control (Figure S3E).

Given that scRNA-seq data of GB patients suggests that multiple immune cell populations may contribute to rIL-12-mediated anti-GB immunity, we sought to reduce the complexity of the TME in our GB mouse models by isolating cells most likely to respond to rIL-12. To achieve this, we enzymatically dissociated rIL-12-treated CT-2A-FLuc tumors and sequentially enriched for immune cell populations using anti-CD11b and anti-CD45 affinity columns (anti-CD11b and anti-CD45) (Figure 2D, left), followed by qRT-PCR analysis of *Il12rb2* expression to confirm successful isolation of potential IL-12-responsive cells (Figure 2D, right). We measured *Il12rb2* transcript levels in tumor-bearing mice that received rIL-12 treatment. In CD45-enriched (EN) tumor (TU) cells (immune cells derived from tumor after anti-CD11b depletion and anti-CD45 column enrichment) were 30.80- and 192.72-fold higher compared with CD11b^EN^ TU (immune cells derived from tumor cells after anti-CD11b column enrichment) and CD45-deprived (DEPR) TU cells (cells derived from tumor after anti-CD11b and anti-CD45 depletion), respectively (Figure 2D, right). To test whether CD45^EN^ TU cells contain NK/T cells—suggested by the human glioma scRNA-seq data to exhibit high levels of IL-12 receptor-related transcripts—we assessed *Ifng* transcript levels, as this cytokine is primarily produced by activated, anti-tumor relevant for NK/T cells^41^ (Figure S3F). *Ifng* was mainly detected in the CD45^EN^ CT-2A TU fraction separated magnetically from the TME following rIL-12 treatment (45.65- and 64.21-fold higher than CD11b^EN^ and CD45^DEPR^, respectively) (Figure S3G).

We confirmed that the expression profiles of relevant IL-12R genes (*Il12rb1*, *Il12rb2*, and *Stat4*) are comparable between human gliomas (Figure 2A) and the murine GB cell lines (CT-2A, GL261, and 005) (Table S2; Figures 2E and S3H), supporting the utility of these mouse models for studying IL-12-mediated mechanisms in GB. The 20%–40% of cells in the NK/T cluster including NK cells, CD4 T cells, and CD8 T cells expressed *Stat4* compared with the other cells (∼1%–5%) in murine GB models (GL261, CT-2A, and 005) (Figure S3I). Based on STAT4p levels, we confirmed that, post-rIL-12 treatment, all NK/T cell types have the potential to activate an IL-12 mediated immune response against the GB cells (Figures 2F and S3J). STAT4p^POS^ cells were found in 12.6% of the CD8 T cells, 3.7% of the CD4 T cells, and 2.3% of the NK1.1 cells in the TME of a CT-2A tumor treated with rIL-12 (Figure S3K). To identify which NK/T cell types contribute to the anti-GB effect of i.t. administered rIL-12, we depleted CD4, CD8, or NK cells systemically in rIL-12-treated mice (Figure 2G). Survival analysis of CT-2A tumor-bearing mice treated i.t. with rIL-12 and intravenously (i.v.) with anti-CD4, anti-CD8, or anti-NK1.1 revealed that only CD8 T cell depletion significantly reduced the rIL-12-mediated anti-GB response compared with the control (IgG) (Figure 2H). Conversely, anti-NK1.1-treated mice with rIL-12 showed a 37% improved survival compared with control (IgG), implying that NK cells can suppress the effect of rIL-12 therapy. Anti-CD4 treatment had no effect on the GB survival post-rIL-12 treatment. This was confirmed by increased tumor growth based on BLI measurements and a reduction in weight (Figures S3L and S3M). We next verified if the TME of an rIL-12-treated CT-2A tumor was changed upon i.v. anti-CD8 administration with flow cytometry of CD11b^EN^ and CD45^EN^ TU cells. CD8^POS^Thy1.2^POS^ T cells were detected only in the CD45^EN^ TU cell fraction of our non-depleted control (IgG) and not in the mice that received i.v. anti-CD8 injections (Figure S3N). As expected, CD11b^EN^ TU cells did not contain CD8^POS^Thy1.2^POS^ T cells. CD8 T cell depletion in an i.t. rIL-12 GB-bearing mouse can also be monitored by analyzing peripheral tissues indicating that the CD8 T cells were depleted not only in the brain but also in the spleen (Figure S3N). Additionally, on days 11 and 18 post-tumor implantation (1 and 8 days after the last i.v. injection with anti-CD8, respectively) lower *Cd8b* levels in the blood were observed due to the depletion, while this was not the case on day 7 (3 days before rIL-12 treatment and 2 days before the first injection with anti-CD8), indicating that our regimen was sufficient for successful depletion overtime (Figure S3O).

Taken together, our data demonstrate that CD8 T cells within the TME are key effectors driving tumor reduction during a rIL-12-mediated response.

### Identifying GB-associated DCs with the potential to stimulate CD8^POS^ T cells during IL-12 treatment

**Figure 3:**
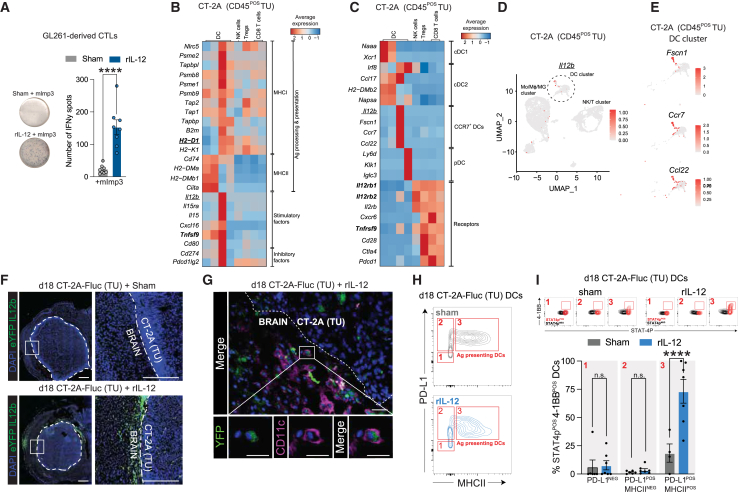
GB-associated DC states with the potential to stimulate CD8^POS^ T cells during IL-12 immunity increase 4-1BB expression (A) IFN-γ production by primary CT-2A-associated CD8^POS^ T cells following rIL-12 exposure and tumor Ag presentation via MHC class I*.* Representative images of the IFN-γ Elispot assay demonstrate an increased number of spots when primary CD8^POS^ T cells, isolated from GL261-bearing mice brain, were co-cultured with naive splenocyte-derived DCs that MHCI-present mImp3, a GL261-specific neopeptide, for 24 h. This increase was observed only in the presence of rIL-12 and not with the sham control (sham = Fc control). Each spot corresponds to an IFN-γ-releasing T cell. The accompanying bar graph quantifies the number of spots (mean number of spots: 22 for sham; 153 for rIL-12). Data represent two independent experiments and are presented as the mean ± SEM (error bars). Data were analyzed using an unpaired t test, ∗∗∗∗*p* < 0.0001. (B) MHCI-expressing DCs at the tumor site express regulatory factors, including Il12b and Tnfsf9, capable of modulating CD8^POS^ T cell activity. Heatmap showing co-expression of genes that are expressed by CT-2A-associated DCs involved in tumor Ag cross-presentation (dataset from Tomaszewski et al.^45^). The listed genes are selected based on their ability to modulate CD8^POS^ T cell activity through stimulatory factors and/or inhibitory factors. *H2-D1* encodes for H-2Db that is an MHC-I class molecule responsible for mImp3 presentation. MHC-I-expressing DCs co-express high levels of CD8^POS^ T cell activity modulating factors, including *Il12b* (encoded by IL-12p40), inhibitory factors, including *Cd274* (encoding for PD-L1), and co-stimulatory factors, including *Tnfsf9* (encoding for 4-1BBL). CT-2A (CD45^POS^) tumor (TU). (C) MHCI-expressing DCs at the tumor site co-express *Il12* receptor and *Tnfrsf9*. Heatmap illustrating that *Il12b*^POS^ DCs co-express *Il12rb1* (encoding the IL12 receptor subunit) and *Tnfrsf9* (encoding 4-1BB) at the CT-2A tumor site. Based on *Ccr7* expression, these *Il12b*^*POS*^ DCs can be classified as *CCR7*^*POS*^ DCs, which display a distinct transcriptional profile compared with (less/non-activated) conventional DCs (cDC1 and cDC2) and plasmacytoid DCs (pDCs). CT-2A (CD45^POS^) tumor (TU) (dataset from Tomaszewski et al.^4^^,^^5^). (D) *Il12b* expression is restricted to a subcluster of DCs*.* UMAP clustering shows expression of *Il12b* in distinct population of the DC cluster in CT-2A (CD45^POS^) tumor (TU) (dataset from Tomaszewski et al.^4^^,^^5^). (E) *Il12b*^*POS*^ DCs have migratory signatures. The cells positive in (D, marked with a dotted line) match with the migratory factors *Fscn1*, *Ccr7*, and *Ccl22*.^118^ DC cluster in CT-2A (CD45^POS^) tumor (TU) (dataset from Tomaszewski et al.^4^^,^^5^). (F) *IL-12-*expressing cells are recruited to the TME of *rIL-12*-treated GB tumors. High numbers of eYFP-expressing cells (in green) were observed in both the CT-2A tumor (TU) border (white dotted line) as well as the tumor itself in IL-12b-eYFP reporter mice treated on day 10 post-tumor implantation with sham control (50 ng of Fc) or 50 ng rIL-12. Mice were sacrificed on day 18 post-tumor implantation (4× magnification). Scale bar, 50 μm. (G) *IL-12b-eYFP-*expressing cells recruited to the GB TME post-*rIL-12* treatment are DCs. CT-2A-Fluc tumor (TU)-bearing mice were treated with rIL-12 and sacrificed on day 18 post-tumor implantation. Samples generated in (F) were stained with anti-CD11c, confirming that eYFP-expressing cells are DCs. Scale bars, 10 μm (10× magnification) and 50 μm (40× magnification). (H) Tumor-engaging and Ag-presenting DCs at the GB tumor site express PD-L1*.* Flow cytometry contour plots of MHC-II versus PD-L1 expression in DCs on day 18 post-tumor (TU) implantation i.t. treated with sham (gray) or rIL-12 (blue). Distinct DC subsets (CD11c^POS^) were identified: PD-L1^NEG^MHC-II^NEG^ (box 1), PD-L1^NEG^MHC-II^POS^ (box 2), and PD-L1^POS^MHC-II^POS^ (box 3), the latter representing activated DCs. (I) Ag-presenting PD-L1^POS^ DCs respond to rIL-12 by increasing 4-1BB expression*.* DCs were isolated from CT-2A tumor (TU) mice on day 18 post-tumor implantation i.t. treated with sham or rIL-12. DCs responsive to rIL-12 were identified based on STAT4p expression and were mainly present in PD-L1^POS^MHC-II^POS^ cells (box 3 of H, represented by the red “3”) and not in the PD-L1^NEG^MHC-II^NEG^ and PD-L1^NEG^MHC-II^POS^ (boxes 1 and 2 of H, represented by the red “1” and “2”) after rIL-12 treatment. 4-1BB was increased in the PD-L1^POS^MHC-II^POS^ pSTAT^POS^ cells (top). Quantification of STAT4p^POS^-expressing cells in 4-1BB^POS^ DCs post-treatment with rIL-12 showed an increased expression of 54.71% ± 15.41% (mean ± SEM) in cells pre-gated for PD-L1^POS^MHC-II^POS^ (box 3 of H) were found in rIL-12-treated cells compared with sham (*n* = 6–7 mice per group) (bottom). The 4-1BB^POS^ DCs are a subset of the STAT4p^POS^ DCs. Data represent two independent experiments and are presented as the mean ± SEM (error bars). Data were analyzed using multiple comparison two-way ANOVA, ∗∗∗∗*p* < 0.0001, n.s.

Although *Il12*^*+/+*^ and *Il12*^−/−^ mice showed no difference in survival of GB patients (Figure 1A), *Il12b* expression was still detectable in the immune compartment of CT-2A tumors after sham treatment from *Il12*^*+/+*^ mice (Figure S4A). This suggests that GB tumors are not entirely devoid of IL-12; however, the levels produced—or the presence of IL-12-producing cells—may be insufficient within the TME to elicit a robust anti-tumor response. Indeed, in a human glioma (*de novo* and recurrent) scRNA-seq dataset, we found that *IL12B* was mainly expressed in the DC cluster (Figure S4B). DCs are known to instruct tumor Ag-reactive T cells, including CD8^POS^ T cells (primarily through subsets such as conventional DC1s and, to a lesser extent, plasmacytoid DCs [pDCs]), to proliferate and activate their cytotoxic machinery.^42^ When CD8^POS^ T cells isolated from GL261 tumors were co-cultured with naive DCs, no effect in the rIL-12 condition (bottom image) was observed, compared with Fc sham control (top image), as assessed by IFN-γ secretion (Figure S4C). However, when DCs were stimulated to cross-present the GL261-neoepitope peptide, mImp3,^43^ followed by rIL-12 (bottom image) incubation, the mouse tumor-derived CD8^POS^ T cells secreted 7-times higher levels of IFN-γ compared with Fc sham controls (top image) (Figure 3A). This indicates that rIL-12 acts as a stimulatory cytokine for GB-associated CD8^POS^ T cells recognizing the DC MHC-I-tumor neoantigen peptide complex via their T cell receptors. Hence, we screened scRNA-seq datasets of human glioma tissue (Figures S4D and S4E) and murine GB models (CT-2A and GL261) (Figures 3B, 3C, S4F, and S4G) to identify DCs in the TME that might be involved in tumor Ag cross-presentation at the tumor site and whether they are equipped to modulate CD8^POS^ T cell activity and/or proliferation through co-stimulatory factors and/or cytokine production. In our analysis, *H2-D1* encoding H-2Db, an MHC-I class molecule that binds the mImp3 peptide,^44^ was highly expressed by two DC clusters in CT-2A (columns 3 and 4 in Figure 3B) and GL261 (columns 7 and 8 in Figure S4F) tumors (datasets from Tomaszewski et al.^45^^,^^46^ and Pombo Antunes et al.^45^^,^^46^ respectively) and was absent in the other tumor-associated DCs. These other DC clusters showed higher expression of genes involved in MHC-II-mediated Ag presentation (including *Cd74*). Interestingly, besides the *H2-D1* expression, one DC subset (column 3 in Figure 3B and column 7 in Figure S4F) excelled in expressing multiple genes important for MHC-I Ag processing and cross-presentation (such as *Psme2* and *TapbpI*). This DC subset also co-expressed high levels of factors that modulate CD8^POS^ T cell activity, including *Il15and Il15ra* (encoding IL-15), inhibitory CD8^POS^ T cell factors, including *Cd274* (encoding PD-L1), and co-stimulatory factors, including *Tnfsf9* (encoding 4-1BBL), while the other *H2-D1*-expressing DC cluster did not (Figures 3B and S4F). Interestingly, this DC subset not only expressed activation markers, but they also had an *Il2b* signature (Figures 3C, 3D, S4G, and S4H) and expressed migratory markers, including *Fscn1* and *Ccr7* which are important in facilitating DC migration to tumor-draining lymph nodes to shuttle between lymph nodes and the CNS. With *Ccl22*, involved in interactions between T regulatory cells and DCs^47^ (Figures 3E and S4I). Based on their *Ccr7* expression, we refer to these cells as CCR7^POS^ DCs (column 3 in Figures 3B and 3C and column 7 in Figures S4F and S4G)—an activated tumor-retained DC subset distinct from pDCs (column 4 in Figures 3B and 3C and column 8 in Figures S4F and S4G) and previously associated with MHC class I-expressing DCs in other tumor contexts.^48^

Drawing conclusions about these activated tumor-associated DCs or CCR7^POS^ DCs in human glioma datasets is challenging due to the low number of MHCI-expressing DCs in these tumors (Figures S4D and S4E). Specifically, in the *de novo* human glioma datasets from Mathewson et al.^46^^,^^49^^,^^50^ Miller et al.^50^ and Pombo Antunes et al.^46^^,^^49^^,^^50^ no *CCR7* expression in the DC cluster was observed. In one recurrent glioma dataset that contained *CCR7*-expressing DCs, we also observed MHC-I components, stimulatory factors including *IL-12B*, inhibitory factors (e.g., *CD274*), and co-stimulatory factors (e.g., *CD80*) that match our murine findings (second heatmap in Figure S4E). We hypothesize that the detection of CCR7^POS^ DCs in that human recurrent glioma dataset (fourth heatmap in Figure S4E) is due to its enrichment for immune cells prior to scRNA-seq processing, whereas the other dataset from Miller et al.^49^ (third heatmap in Figure S4E) did not perform this enrichment. Of note, the murine 005 dataset (Table S2) contained no relevant DC information.

Based on scRNA-seq analysis, *Il12b*^POS^ DCs are equipped with IL-12R machinery (mainly *Il12rb2*) and thus have the potential to be affected by rIL-12 (Figures 3C and S4G). Notably, scRNA-seq data also suggest that NK/T cells are more responsive to rIL-12 than CCR7^POS^ DCs, as they are co-expressing both *Il12rb1* and *Il12rb2*, the two subunits required for functional IL-12 receptor signaling. We assessed the involvement of CCR7^POS^ DCs in rIL-12-mediated immunity at the GB site in a preclinical mouse model. Using IL-12b^YFP^ reporter mice^51^ to label CCR7^POS^ DCs, we observed an accumulation of IL-12b^YFP^ cells at the CT-2A tumor site upon i.t. rIL-12 injection, but not after administering sham (Figures 3F and S4J). Tumor-associated IL-12b^YFP^-positive cells were confirmed to be DCs as they co-expressed CD11c (Figure 3G). Next, we analyzed the activity status of DCs upon rIL-12 treatment. Tumor-interacting and Ag-presenting CD11c^POS^ DCs post-rIL12/sham treatment of our CT-2A-bearing mice were identified by PD-L1 and MHC-II markers, respectively (Figure 3H). CCR7^POS^ DCs were predicted to express 4-1BB (*Tnfrsf9*) based on transcriptomic data (Figures 3C and S4G), which we confirmed at the protein level within the PD-L1^POS^MHC-II^POS^ population (Figure 3I, top panel). The PD-L1^POS^MHC-II^POS^4-1BB^POS^ DCs also displayed most of the rIL-12 reactivity based on STAT4p expression, the downstream signaling event of the IL-12 receptor pathway.^52^ rIL-12 treatment resulted in a 54.71% ± 15.41% (mean difference ± SEM) increase of PD-L1^POS^MHC-II^POS^4-1BB^POS^STAT4p^POS^ DCs at the tumor site compared with sham (Figure 3I, bottom bar graph).

Taken together, our findings suggest that, in addition to CD8^POS^ T cells, DCs also accumulate and become activated at the tumor site in response to exogenous rIL-12. These intratumoral-affected DCs are rare in non-stimulated tumors and have distinct signatures, migratory markers, and properties compared with other DCs and have the potential to modulate the CD8^POS^ T cell response, including providing stimulatory signals (e.g., co-stimulatory molecules and cytokines) to tumor-Ag-targeting CD8^POS^ T cells.

### I.t. injected rIL-12 increases the number of effector-like CD8^POS^ T cells at the tumor site

**Figure 4:**
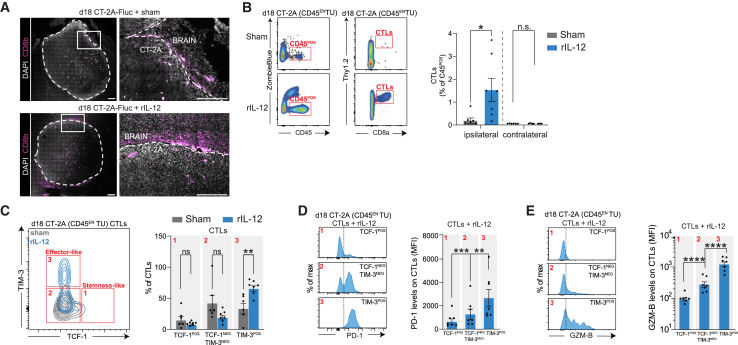
I.t.-injected rIL-12 increases the number of effector-like CD8^POS^ T cells at the tumor site (A) Higher number of CD8^POS^ T cells around rIL-12-treated CT-2A-FLuc tumor*.* Immunofluorescent imaging of CD8b at the tumor border (white dotted line) after 18 days of CT-2A-FLuc implantation comparing sham and rIL-12-treated mice (magnification 20×). Scale bar, 100 μm. (B) Increase of CD8^POS^ T cells at tumor site with intratumoral rIL-12*.* Representative flow cytometry plots of CD45^EN^ TU CT-2A tumor cells show the gating for live cells based on uptake of the viability dye ZombieBlue staining. Isolation of Thy1.2^POS^ and CD8^POS^ T cells, pre-gated for CD45^POS^CD11b^NEG^ in brains of tumor-bearing mice on day 18 post-tumor implantation, comparing rIL-12-treated and sham control mice (left). The brain tissue was enzymatically digested and enriched for CD45^EN^ tumor cells. The bar graph represents the quantification of CD8^POS^ T cell numbers in ipsilateral and contralateral hemispheres (*n* = 6 mice per group) as a percentage of CD45^POS^ cells in rIL-12- or sham-treated mice (right, bar graph). Data represent two independent experiments and are presented as the mean ± SEM (error bars). Data were analyzed using unpaired t test, ∗*p* < 0.05. (C) Increased CD8^POS^ T cell differentiation upon rIL-12 treatment of GB. Overlaid flow cytometry plots of CD8^POS^ T cells expressing TCF-1 against TIM-3 while comparing rIL-12 (blue) and sham (gray) treatment (left). Tumor tissue was harvested on day 18 post-tumor (CT-2A CD45^EN^ TU) implantation. Quantification of the percentage of CD8^POS^ T cells comparing TCF-1^POS^ (box 1, effector-like), TCF-1^NEG^TIM-3^NEG^ (box 2), and TIM-3^POS^ (box 3, stemness-like) showed a 32.2% increase of TIM-3^POS^ after rIL-12 (65.5%) treatment compared with sham (33.2%) (*n* = 6–7 mice per condition, right bar graph). Data represent two independent experiments and are presented as the mean ± SEM (error bars). Data were analyzed using two-way ANOVA ∗∗*p* < 0.01, n.s. (D) The activation-induced marker PD-1 is highly expressed in effector-like CD8^POS^ T cells*.* Percent of maximum PD-1 expression within TCF-1^POS^ (box 1), TCF-1^NEG^TIM-3^NEG^ (box 2), and TIM-3^POS^ (box 3) populations on day 18 post-tumor implantation (CT-2A CD45^EN^ TU), of CD8^POS^ T cells when treated with rIL-12 (left). The black dotted vertical line represents the FMO signal. Quantification of PD-1 levels per CD8^POS^ T cell by flow cytometry after rIL-12 treatment within TCF-1^POS^ (box 1 in C, MFI 2679), TCF-1^NEG^TIM-3^NEG^ (box 2 in C, MFI 1280), and TIM-3^POS^ (box 3 in C, MFI 652) populations. TIM-3^POS^ cells express significantly more PD-1 compared with TCF-1^POS^ and TIM-3^NEG^TCF-1^NEG^ cells. In contrast, TIM-3^NEG^TCF-1^NEG^ CD8^POS^ T cells express significantly more PD-1 per cell, compared with TCF-1^POS^ ones (right). Data represent two independent experiments and are presented as the mean ± SEM (error bars). Data were analyzed using two-way ANOVA, ∗∗*p* < 0.01, ∗∗∗*p* < 0.001. (E) Cytotoxic GZM-B is highly expressed in effector-like CD8^POS^ T cells*.* Percent of maximum GZM-B expression within TCF-1^POS^ (box 1 in C), TCF-1^NEG^TIM-3^NEG^ (box 2 in C), and TIM-3^POS^ (box 3 in C) populations on day 18 post-tumor (CT-2A CD45^EN^ TU) implantation, of CD8^POS^ T cells when treated with rIL-12 (left). The black dotted vertical line represents the FMO signal. Quantification of GZM-B levels per CD8^POS^ T cell by flow cytometry after rIL-12 treatment within TCF-1^POS^ (box 1 in C, MFI 1229), TCF-1^NEG^TIM-3^NEG^ (box 2 in C, MFI 277.3) and TIM-3^POS^ (box 3 in C, MFI 93.5) populations (*n* = 5–6 mice per group) (right). Data represent two independent experiments and are presented as the mean ± SEM (error bars). Data were analyzed using two-way ANOVA, ∗∗∗∗*p* < 0.0001.

We have shown that the survival benefit from i.t. rIL-12 is driven by CD8^POS^ T cells and that local DCs may provide the necessary signals to guide their activity. Here, we investigate whether rIL-12-induced, tumor-associated CD8^POS^ T cells have the potential to sense cues from rIL-12-activated DCs to enhance their functionality. CD8^POS^ T cells were present around the CT-2A tumor treated i.t. with either rIL-12 or sham (Figures 4A and S5A). Multiparametric flow cytometry was performed on CD45^EN^ TU cells to quantify CD8^POS^ T cells in the tumor hemisphere. rIL-12 treatment showed a 10-fold increase in CD8^POS^ T cells compared with sham-treated GB mice (Figure 4B). CD8^POS^ T cell accumulation in the tumor appeared to be tumor targeted, as we could not detect CD8^POS^ T cells in the contralateral hemisphere of either rIL-12 or sham-treated mice. Interestingly, the number of CD8^POS^ T cells was consistently low in our sham-treated tumors, underscoring the immunosuppressive nature of the CT-2A model.^53^ This conclusion is further supported by evaluating survival of mice that received sham treatment after CT-2A implantation comparing CD8^POS^ T cell-depleted and non-depleted mice, where no differences in survival were found (Figure S5B), and no differences in BLI or mice weights (Figure S5C). This was also observed in the human glioma survival analysis as high CD8a levels did not result in improved survival outcomes compared with low *CD8A* levels (Figure S5D).

Next to accumulation and proliferation of local CD8^POS^ T cells, CD8^POS^ T cell activity is necessary for tumor regression. However, the immunosuppressive nature of GB reduces CD8^POS^ T cell activity and can drive CD8^POS^ T cells into a hypo- or dysfunctional state.^54^ Countering this tumor-enforced immunosuppressive program has been a major challenge in the field as it is crucial for sustained anti-tumor activity by CD8^POS^ T cells.^54^

To study the effect of rIL-12 on CD8^POS^ T cell activity, we screened for relevant activity markers in GB-associated CD8^POS^ T cells using scRNA-seq datasets derived from human glioma (both *de novo* and recurrent) (Figures S5E–S5H) and murine GB models (CT-2A, GL261, and 005) (Figures S5I–S5K). Immune cells such as NK cells and T cells were distinguished based on specific markers, such as *KLRB1C* on NK cells, and *CD3D/E* on T cells. Helper and regulatory T cells were identified by expression of *CD4* and *FOXP3* genes, and CD8^POS^ T cells were marked by expression of *CD8A*/*B*. The CD8^POS^ T cell population has distinct subtypes, such as stem-like memory CD8^POS^ T cells, which have self-renewing capabilities, and effector-like CD8^POS^ T cells, which carry out cytotoxic functions targeting cells presenting tumor Ags.^55^ We observed the expression of signature genes *TCF7* (encoding TCF-1) and *HMP19* (encoding NSG2) in naive and stem-like CD8^POS^ T cells. In contrast, effector-like CD8^POS^ T cells express genes such as *HAVCR2* (encoding TIM-3), *PDCD1* (encoding PD-1), and *GZMB* (encoding granzyme-B), which are markers of their cytotoxic function and inhibitory state.^56^^,^^57^^,^^58^^,^^59^ In addition, we observed co-expression of *IL12RB1*, *IL12RB2*, and *STAT4*, markers of interest in both stem-like and effector-like CD8^POS^ T cells (Figures S5E and S5F). The same states and expression patterns of markers were found in the murine models (Figures S5I–S5K).

Next, we performed an analysis of the rIL-12 mediated response based on these identified markers. We characterized whether CD45^EN^ TU cells contained effector-like CD8^POS^ T cells using the markers *Pdcd1* (encoding PD-1),^60^ *Gzmb* (encoding granzyme-B),^61^ and *Cd101* (encoding CD101),^57^ which are signatures for tumor Ag reactivity, cytotoxicity, and differentiation, respectively (Figure S5L). Upon rIL-12 treatment the markers *Pdcd1*, *Gzmb*, and *Cd101* increased by 5.60-, 3.0-, and 2.9-fold in the CD45^EN^ TU samples, respectively, compared with sham-treated tumors^62^^,^^63^ (Figure S5M). We then performed an in-depth characterization of CD8^POS^ T cell states during rIL-12 treatment with a multiplex flow cytometric analysis of CD45^EN^ TU cells. Eighteen days post-tumor implantation (equivalent to 8 days post-rIL-12/sham treatment), 32.3% ± 9.1% (mean difference ± SEM) more TCF-1^NEG^ TIM-3^POS^ CD8^POS^T cells were observed compared with the sham-treated GB mice, indicating a transition of CD8^POS^ T cells from a stem-like to an effector-like state (Figure 4C). A prerequisite for effector-like CD8^POS^ T cells is that they engage with a tumor Ag-(cross-)presenting cell, which provides the necessary signals for them to acquire cytotoxic potential. PD-1 is transiently upregulated on CD8^POS^ T cells upon their interaction with a cross-presented Ag via their T cell receptor. Upon rIL-12 treatment, the TCF-1^NEG^TIM-3^POS^ CD8^POS^ T cells expressed the highest levels of PD-1 (4-fold higher compared with TCF-1^POS^TIM-3^NEG^ and 2-fold higher compared with TCF-1^NEG^TIM-3^NEG^), indicating that this CD8^POS^ T cell state had experienced prolonged or repeated engagement with their T cell receptor compared with the other states^60^ (Figures 4D and S5N). Next, we checked the tumor-killing potential of the TCF-1^NEG^TIM-3^POS^ CD8^POS^ T cells by determining their cytotoxic GZM-B levels. GZM-B levels gradually increased along the TCF-1-to-TIM-3 differentiation axis upon rIL-12 treatment, accentuating cytotoxic activity in TCF-1^NEG^TIM-3^POS^ CD8^POS^ T cells (GZM-B MFI levels were 13-fold higher compared with TCF-1^POS^TIM-3^NEG^, and 4-fold higher compared with TCF-1^NEG^TIM-3^NEG^) (Figure 4E).

In sum, the above data demonstrate that i.t. rIL-12-administration results in an increased number of CD8^POS^ T cells at the tumor site that progress toward an effector-like state.

### Effector-like CD8^POS^ T cells in the GB TME sustain 41BB expression post-rIL-12 treatment

Effector-like CD8^POS^ T cells require various signals to become activated and functional, including Ag recognition (signal 1), co-stimulatory signals (signal 2), and cytokine signaling (signal 3).^64^^,^^65^ We have shown that tumor-associated CD8^POS^ T cells were susceptible to DC-provided MHC-I-mediated Ag cross-presentation (signal 1) and rIL-12-based cytokine stimulation (signal 2) (Figure 3A), but we could not verify co-stimulation (signal 3). Previously, we suggested that rare *Ccr7*^POS^ DCs have the potential to provide these co-stimulatory signals, including *Tnfsf9* (encoding for 4-1BB ligand or 4-1BBL in Figure 3B) in a GB tumor. Here, we verified that the rIL-12-stimulated CD8^POS^ T cell states (e.g., effector-like CD8^POS^ T cells) have the machinery to bind to 4-1BBL provided by *Ccr7*^POS^ DCs at the tumor site. Indeed, the scRNA-seq datasets (*Tnfrsf9* in Figures 3C, S4D, S4E, and S4G) indicate that, next to *Ccr7*^POS^ DCs, CD8^POS^ T cells^66^^,^^67^ might express the co-stimulatory receptor 4-1BB.^68^ This suggests that stimulation by the 4-1BBL can be provided by intratumoral *Ccr7*^POS^ DCs at the tumor site (*Tnfsf9* in Figures 3B and S4F), which could potentially act on both CD8^POS^ T cells and adjacent DCs, thereby strengthening the anti-tumor response. Because 4-1BBL can bind to and activate 4-1BB on both cell types, similar to rIL-12 binding to IL12R on CD8^POS^ T cells and DCs (Il12rb2 in Figures 3C, S4E, and S4G), it suggests a potential dual role for 4-1BBL in enhancing immune responses. This pattern of activity was not observed with other co-stimulatory molecules, such as *Cd80*, which showed more cell type-specific effects. Notably, its receptor *Cd28* was predominantly expressed in the NK/T cell cluster and not in DCs.

**Figure 5:**
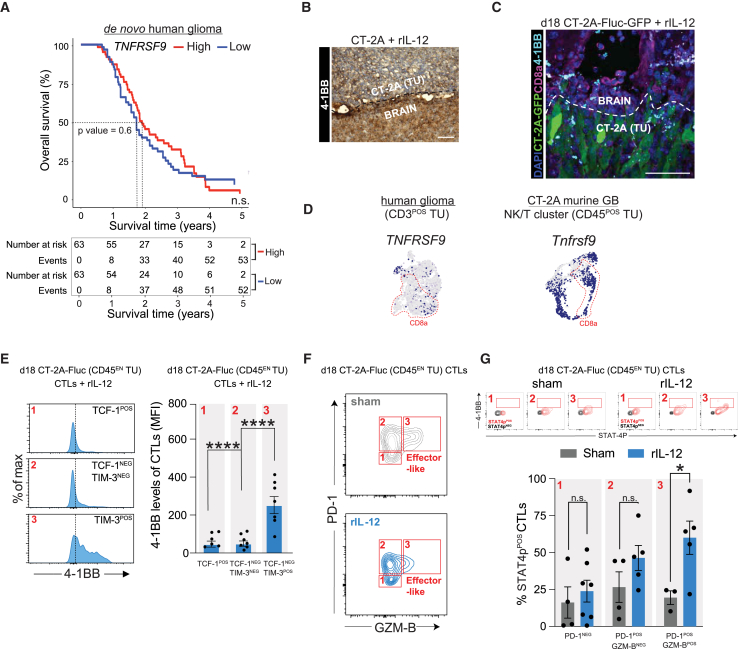
In line with rIL-12-activated DCs, effector-like CD8^POS^ T cells at the GB border elevate 41BB expression post-rIL-12 treatment (A) Survival probability of *de novo TNFRSF9.* Kaplan-Meier survival curves showing the survival outcomes over a period of 5 years of 63 GB patients (IDH-WT) per group with high (red) or low (blue) levels of *TNFRSF9*, each group had a median of ∼2 years (based on Miller et al.^50^). No differences were observed between groups. Log rank (Mantel-Cox) test, *p* = 0.6, n.s. (B) Immunohistochemistry shows 4-1BB expression at the tumor border*.* The CT-2A-bearing brain treated with rIL-12 was isolated at day 18 post-implantation and stained for 4-1BB. Brown color represents HRP signal (magnification 20×). Scale bar 100 μm. (C) CD8^POS^ T cells expressing 4-1BB are recruited at the tumor upon rIL-12 treatment. Immunofluorescence shows that 4-1BB (cyan) was expressed in CD8^POS^ T cells (pink) post-rIL-12 treatment at day 18 post-implantation at the CT-2A-Fluc-GFP (green) tumor (TU) border (white dotted line) (20× magnification). Scale bar, 50 μm. (D) 4-1BB expression in CD8^POS^ T cells. scRNA-seq analysis displaying *TNFRSF9* expression in *CD3*^*POS*^ tumor (TU) cells of *de novo* human glioma and *Tnfrsf9* expression in the NK/T cluster of CD45^POS^ tumor (TU) cells in CT-2A murine GB.^118^ The red dotted line represents the CD8a population. (E) 4-1BB is expressed by effector-like CD8^POS^ T cells during rIL-12 treatment*.* Percent of maximum 4-1BB expression (left histogram plot) within TCF-1^POS^ (box 1 in Figure 4C), TCF-1^NEG^TIM-3^NEG^ (box 2 in Figure 4C), and TIM-3^POS^ (box 3 in Figure 4C) populations on day 18 post-tumor (CT-2A CD45^EN^ TU) implantation of CD8^POS^ T cells when treated with rIL-12. The black dotted vertical line represents the FMO signal. Quantification by 4-1BB levels in *CD8*^*POS*^ T cells by flow cytometry (right bar graph) after rIL-12 treatment within TCF-1^POS^ (box 1 in Figure 4C, MFI: 251.9), TCF-1^NEG^TIM-3^NEG^ (box 2 in Figure 4C, MFI: 48.4) and TIM-3^POS^ (box 3 in Figure 4C, MFI: 46.3) populations (*n* = 4–7 mice per group). Data represent two independent experiments and are presented as the mean ± SEM (error bars). Data were analyzed using two-way ANOVA, ∗∗∗∗*p* < 0.0001 (datasets from Tomaszewski et al. and Miller et al.^45^^,^^50^). (F) Effector-like CD8^POS^ T cells can be identified by PD-1 and GZM-B during rIL-12 treatment. Counter plots of GZM-B expression against PD-1 within PD-1^NEG^ (box 1), PD-1^POS^GZM-B^NEG^ (box 2), and PD-1^POS^GZM-B^POS^, representing effector-like cells (box 3) populations on day 18 post-tumor (CT-2A CD45^EN^ TU) implantation of CD8^POS^ T cells when treated with sham (top) or rIL-12 (bottom). (G) 4-1BB^POS^ cells are more present in the CD8^POS^ T cell effector-like subset that is STAT4 phosphorylated during rIL-12 treatment. Cells were pre-gated for 4-1BB versus STAT4p comparing sham and rIL-12-treated conditions (top). Quantification by flow cytometry as shown in bar graphs of percentage of 4-1BB^POS^ cells post-rIL-12 treatment showing STAT4 phosphorylation of CD8^POS^ T cells within PD-1^NEG^ (box 1 in F), PD-1^POS^GZM-B^NEG^ (box 2 in F), and PD-1^POS^GZM-B^POS^ (box 3 in F) populations (bottom) (*n* = 3–7 mice per group). Data represent two independent experiments and are presented as the mean ± SEM (error bars). Data were analyzed using two-way ANOVA, ∗*p* < 0.05.

In GB, 4-1BB is not detectable compared with healthy brain tissue, likely due to suppressed expression of its ligand, 4-1BBL (Figure S6A). This lack of 4-1BB signaling in *de novo* GB may explain why no survival benefit is observed between patients with high versus low 4-1BB(L) expression levels (Figures 5A and S6B). Despite this observation, we were still able to detect 4-1BB^POS^ CD8^POS^ T cells at the tumor site in our murine GB model (Figures 5B, 5C, and S6C) and not in the spleen (Figure S6D). In the scRNA-seq datasets, we confirmed that effector-like CD8^POS^ T cells at the tumor site can express *TNFRSF9* (encoding for 4-1BB) (Figure 5D). Our findings in the *de novo* GB were validated in a recurrent glioma dataset (Figures S5E and S5F) and in CT-2A, GL261, 005 murine models (Figures 5D, S4D–S4G, S5J, and S5K). We further characterized the 4-1BB levels in the CT-2A-associated CD8^POS^ T cells upon rIL-12 treatment with flow cytometry. TCF-1^NEG^TIM-3^POS^CD8^POS^ T cells (effector-like T cells) expressed ∼5-fold more 41BB compared with TCF-1^POS^TIM-3^NEG^ or TCF-1^NEG^TIM-3^NEG^ cells (Figure 5E). The increase in 4-1BB of CD8^POS^ T cells was only observed at the tumor site (Figure S6D) and illustrates that only tumor-engaged CD8^POS^ T cells express 41BB.

By analogy to the previous 4-1BB^POS^*Ccr7*^POS^ DC analysis, we tested whether the 4-1BB^POS^CD8^POS^ T cells in a CT-2A model are rIL-12 responsive by analyzing STAT4-phosphorylation (STAT4p). In effector-like CD8^POS^ T cells, as identified by PD-1 and GZM-B markers (Figure 5F), we detected a 4-1BB^POS^ subpopulation that was enriched for STAT4p^POS^ cells (Figure 5G, top). rIL-12 treatment led to a 41.4% ± 1.131% (mean difference ± SEM) increase in STAT4p^POS^ in this 4-1BB^POS^ effector-like CD8^POS^ T cell subpopulation compared with the sham condition (Figure 5G, bottom). In other CD8^POS^ T cell states (either PD-1^NEG^GZM-B^POS^ or PD-1^NEG^ CD8^POS^ T cells), low numbers of CD8^POS^ T cells co-expressing 4-1BB were observed. Therefore, STAT4 phosphorylation did not increase compared with sham control (Figure 5G).

Taken together, our findings demonstrate that effector-like CD8^POS^ T cells at the tumor site during rIL-12 treatment express the 4-1BB receptor, making them susceptible to activation by 4-1BBL.

### Anti-tumor immunity triggered by combined rIL-12 and 4-1BBL immune stimuli enhanced survival in GB-bearing mice

Clinical trials with therapeutic i.t. IL-12 expression suggested that the activity of CD8^POS^ T cells was rapidly diminished due to the PD-L1-rich GB environment.^21^ Rather than inhibiting immunosuppression with ICI, which failed to enhance IL-12-mediated survival in GB patients,^10^^,^^11^^,^^12^^,^^13^^,^^14^^,^^15^^,^^16^ here we aimed to boost survival by providing a co-stimulatory molecule to support tumor-associated CD8^POS^ T cells directly, or indirectly through DCs. The co-stimulatory factor *TNFSF9* (encoding 4-1BBL) was selected because it was poorly induced in GB tissue, to the extent that patients with detectable *TNFSF9* (4-1BBL) expression levels did not gain in survival (Figures S6A and S6B).

**Figure 6:**
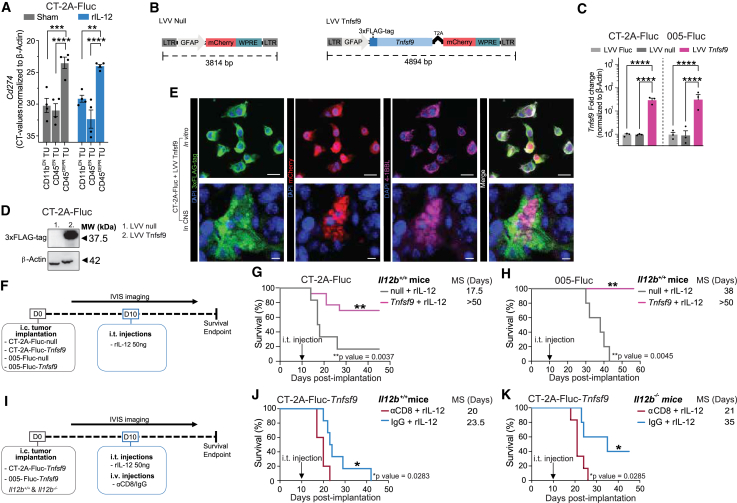
Anti-tumor immunity activated by combined rIL-12 and *Tnfsf9* immune stimuli increased the survival of GB-bearing mice (A) GB in mice express high levels of PD-L1 in non-immune compartment after rIL-12 treatment. CD11b^EN^, CD45^EN^, and CD45^DEPR^ TU populations were isolated from CT-2A-FLuc tumor-bearing hemisphere of brains using CD11b beads and CD45 beads (see Figure 2D). *Cd274* (encoding the PD-L1 gene) was expressed at significantly higher levels in CD45^DEPR^ tumor (TU) cells. No differences were observed between sham (gray) and rIL-12 treatment (blue). Gene expression levels were normalized to β-actin (*n* = 4 mice per group). Data represent four independent experiments and are presented as the mean ± SEM (error bars). Data were analyzed using one-way ANOVA, ∗∗*p* < 0.01, ∗∗∗*p* < 0.001, ∗∗∗∗*p* < 0.0001. (B) Lentivirus vector constructs expressing *Tnfsf9* or null*.* Schematic display of *Tnfsf9* lentivirus vectors (LVV); LVV null, a GFAP promotor followed mCherry and WPRE (left); LVV *Tnfsf9* containing mCherry labeled *Tnfsf9* and 3xFLAG-tag driven by a GFAP promotor (right). (C) *Tnfsf9* expression in GB mouse cells*.* CT-2A-FLuc and 005-FLuc cells transduced with the LVV *Tnfsf9* showed significant enhanced gene expression levels of *Tnfsf9* (encoding for 4-1BBL) compared with cells transduced with LVV null and non-transduced cells, normalized to β-actin. Data represent three independent *in vitro* experiments and are presented as the mean ± SEM (error bars). Data were analyzed using one-way ANOVA, ∗∗∗∗*p* < 0.0001. (D) 4-1BBL protein expression in CT-2A cells. 3xFLAG-tag protein levels (37.5 kDa) were only present in CT-2A-FLuc cells transduced with the LVV *Tnfsf9* compared with *in vitro* CT-2A cells transduced with LVV null, normalized to β-actin. 3xFLAG-tag detection enabled detection of transgene 4-1BBL and not endogenous 4-1BBL. (E) Homogenous 4-1BBL expression in transduced GB mouse cell line for brain implantation experiments. Immunofluorescent images of 4-1BBL overexpressing CT-2A cells post-LVV *Tnfsf9* transduction in culture stained for DAPI (blue), 3xFLAG-tag (green), mCherry (red), and 4-1BBL (pink) with a merged image. Scale bar, 50 μm. Tumor-bearing mouse brains confirmed transgene expression (mCherry-positive cells) co-localized with 3xFLAG-tag and 4-1BBL (40× magnification). Scale bar, 50 μm. (F) Experimental outline to test therapeutic effect of local expression of *Tnfsf9* and rIL-12 treatment. The *in vivo* approach is schematically displayed: CT-2A-FLuc-null, CT-2A-FLuc-*Tnfsf9*, 005-FLuc-null, and 005-FLuc-*Tnfsf9* cells (100,000 cells) were implanted i.c. and mice were treated i.t. with rIL-12 or sham (PBS or Fc control) 10 days after tumor implantation. (G) Survival benefit of local *Tnfsf9* expression in CT-2-FLuc-bearing mice post-rIL-12 treatment. Kaplan-Meier curves showing survival outcomes following treatment of CT-2A-FLuc-control with rIL-12 (solid gray) or CT-2A-FLuc-*Tnfsf9* treated with rIL-12 (solid pink) (*n* = 4–5 mice per group). Mice injected with CT-2A-FLuc-*Tnfsf9* tumor cells treated with rIL-12 (50 ng) had a median survival of >50 days (*p* = 0.0037) compared with mice implanted with tumor cells lacking 4-1BBL, median survival of 17.5 days. Data represent at least two independent experiments and are presented as the mean ± SEM (error bars). Data were analyzed using log rank (Mantel-Cox) test, ∗∗*p* < 0.01. Median survival in days (MS). (H) Survival benefit of local *Tnfsf9* expression in 005-FLuc-bearing mice post-rIL-12 treatment. Kaplan-Meier curves showing survival outcomes following treatment of 005-FLuc-control with rIL-12 (solid gray) or 005-FLuc-*Tnfsf9* treated with rIL-12 (solid pink) (*n* = 4–5 mice per group). Mice injected with 005-FLuc-*Tnfsf9* tumor cells treated with rIL-12 (50 ng) had a 100% survival (*p* = 0.0045) compared with mice implanted with tumor cells lacking *Tnfsf9*, median survival of 38 days. Data represent at least two independent experiments and are presented as the mean ± SEM (error bars). Data were analyzed using log rank (Mantel-Cox) test, ∗∗*p* < 0.01. Median survival in days (MS). (I) Experimental outline to test CD8 T cell dependency of *Tnfsf9* and rIL-12 combination treatment. Schematic display shows i.v. injection with or without CD8 T cell depletion (αCD8 or IgG control, respectively) on days 9 and 10 (50 and 100 μg on days 9 and 10, respectively) post-tumor (CT-2A-FLuc-*Tnfsf9*, 005-Fluc-*Tnfsf9*, 100,000 cells) implantation. Mice were injected i.t. with rIL-12 50 ng on day 10. (J) GB mouse survival benefit from *Tnfsf9* and rIL-12 combination treatment is CD8 T cell dependent*.* Kaplan-Meier curves of *Il12*^*+/+*^ mice showing survival outcomes of CT-2A-FLuc-*Tnfsf9* tumor-bearing mice all i.t. treated with rIL-12, after treatment with αCD8 (red) or IgG control (blue). Mice (*n* = 5–6 mice per group) treated with IgG control had a median survival of 23.5 days (*p* = 0.0283), compared with 20 days for mice treated with αCD8. Data represent two independent experiments and are presented as the mean ± SEM (error bars). Data were analyzed using log rank (Mantel-Cox) test, ∗*p* < 0.05. Median survival in days (MS). (K) GB mouse survival benefit from CD8 T cell recruitment induced by the *Tnfsf9* and rIL-12 combination treatment is not dependent on endogenous IL-12*.* Kaplan-Meier curves of *Il12*^−/−^ mice showing survival outcomes of CT-2A-FLuc-*Tnfsf9* tumor-bearing mice all treated with i.t. rIL-12, after treatment with αCD8 (red) or IgG control (blue). Mice (*n* = 5–6 mice per group) treated with IgG control had a median survival of 35 days (*p* = 0.0285), compared with 21 days for mice treated with αCD8. Data represent at least two independent experiments and are presented as the mean ± SEM (error bars). Data were analyzed using log rank (Mantel-Cox) test, ∗*p* < 0.05. Median survival in days (MS).

First, we determined whether our mouse model would be able to mimic the high levels of PD-L1 at the GB site during rIL-12 treatment. Indeed, *Cd274* (encoding PD-L1) was ∼125-fold increased, on tumor cells (CD45^DEPR^ TU cell fraction) compared with the immune cell fraction (represented by both the CD11b^EN^ and CD45^EN^ TU cells) (Figure 6A). We next addressed whether rIL-12-activated tumor-associated 4-1BB^POS^ immune cells (CD8^POS^ T cells and *Ccr7*^POS^ DCs) could be stimulated to further enhance anti-tumor regression in PD-L1-rich GB. To explore this, we designed an LVV encoding murine 4-1BBL (*Tnfsf9*) (Figure 6B). LVV-encoded *Tnfsf9* could be distinguished from endogenous *Tnfsf9* through an N-terminal 3xFLAG-tag, which in the recombinant protein localizes to the intracellular-facing side, ensuring that it does not interfere with 4-1BB binding. A T2A protease cleavage site separating mCherry fluorescent reporter transgene was included to confirm the transduction of tumor cells. An inactive mimic LVV (LVV null), encoding mCherry but lacking the 3xFLAG-tag and *Tnfsf9*, was designed as a control. Following LVV *Tnfsf9* transduction of CT-2A-FLuc and 005-FLuc cells, mCherry^POS^ cells were sorted via FACS and confirmed to overexpress *Tnfsf9* compared with LVV null cells. Our qRT-PCR analysis indicated a 30- and 38-fold increase of *Tnfsf9* (encoding 4-1BBL) in both CT-2A and 005 GB cell lines, respectively, compared with the controls (LVV null and non-transduced) (Figure 6C). Western blot analysis was performed using 3xFLAG-tag detection to confirm that the transgene was expressed as a full-length protein with the expected size of 37.5 kDa (Figure 6D). We validated the uniform recombinant protein expression in the transduced cells through colocalization of mCherry fluorescence with anti-3xFLAG-tag and anti-4-1BBL staining (Figure 6E). Importantly, although *in vitro* all transduced cells expressed the construct, *Tnfsf9* and 3xFLAG-tag expression was not uniformly observed throughout CT-2A-FLuc-*Tnfsf9* tumors on day 18 post-implantation. We hypothesize that this might be due to transgene instability in tumor cells, promoter inactivity or silencing, or potential overgrowth by small numbers of non-expressing *Tnfsf9* GB cells, rather than an effect of rIL-12 treatment.

Next, we assessed the survival rates of GB-bearing mice in response to rIL-12 and tumors expressing *Tnfsf9* (Figure 6F). Mouse brains were engrafted with CT-2A-FLuc- *Tnfsf9* or CT-2A-FLuc-null GB cells and treated injected i.t. with rIL-12 or sham on day 10 post-implantation. The observed increase in survival was attributed to a host-mediated effect, as no differences in cell proliferation were detected between CT-2A-FLuc-*Tnfsf9* and CT-2A-FLuc-null cell lines following *in vitro* exposure to rIL-12 or sham treatment (Figure S7A). Mice implanted with CT-2A-FLuc-*Tnfsf9* tumors and treated with rIL-12 showed a prolonged median survival compared with those implanted with CT-2A-FLuc-null cells and treated with rIL-12 (Figures 6G, S7B, and S7C). The survival advantages following rIL-12 treatment with the co-stimulatory signal *Tnfsf9* were confirmed in the 005 GB mouse model, showing that all mice implanted with 005-FLuc-*Tnfsf9* survived for >50 days compared with mice implanted with 005-FLuc-null cells, which had a median survival of 38 days (Figure 6H). Mice that survived after initial implantation of CT-2A-FLuc-*Tnfsf9* or 005-FLuc-*Tnfsf9* cells were rechallenged with a second tumor (CT-2A-FLuc and 005-FLuc, respectively). Interestingly, only 5 out of 13 re-implanted mice developed new tumors in the CT-2A GB model, suggesting that protective immunity had developed during the rejection of *Tnfsf9*-expressing tumors. All 005-FLuc re-implanted mice did not regrow tumors (Table S4).

To evaluate the impact of co-stimulatory 4-1BBL on CD8^POS^ T cells during an rIL-12-mediated anti-tumor response, mice were systemically depleted of CD8^POS^ T cells with systemic anti-CD8 mAb to avoid CD8^POS^ T cell accumulation at the CT-2A-FLuc-*Tnfsf9* tumor site (Figure 6I). Improved median survival of 3.5 days was observed for non-depleted (IgG control) compared with the T cell-depleted (anti-CD8) CT-2A-FLuc-*Tnfsf9* implanted mice i.t. treated with rIL-12, indicating that the therapeutic effect remains dependent on CD8^POS^ T cells (Figures 6J and S7D). Next, we tested whether enhancing co-stimulation with *Tnfsf9* in absence of endogenous IL-12 could drive tumor regression, given that IL-12 is strongly suppressed at the TME in GB patients. Similar to *Il12b*^*+/+*^ mice, rIL-12 treatment improved median survival compared with the sham treatment in *Il12b*^−/−^ mice implanted with CT-2A-FLuc-*Tnfsf9* cells (Figures S7E and S7F), and depletion of CD8^POS^ T cells reversed the increase in survival (Figures 6K and S7G).

Interestingly, host-derived IL-12 did not appear to play a major role in the anti-tumor response to rIL-12 monotherapy (Figure S7H), but it was critical for the efficacy of *Tnfsf9* monotherapy and the combination treatment. In *Il12b*^*+*^*/*^*+*^ mice that did not receive rIL-12, *Tnfsf9* expression by the tumor cells significantly extended median survival by 27 days compared with CT-2A-FLuc-null control condition (Figure S7B). This survival benefit was lost in *Il12b*^−/−^ mice, where *Tnfsf9* expression without rIL−12 failed to improve outcomes, and survival was comparable with untreated controls (Figures 1A and S7E). These findings suggest that endogenous IL-12 supports the therapeutic activity of *Tnfsf9*. However, when *Tnfsf9* expression at the tumor was combined with rIL-12, survival outcomes were similar across genotypes: 69.2% of Il12b^+/+^ mice (Figures 6H and S7B) and 63.6% of Il12b^−/−^ mice (Figure S7E) survived beyond 50 days, indicating that exogenous rIL-12 can compensate for the absence of endogenous IL-12. These results suggest that, prior to rIL-12 administration (i.e., before day 10), early immune engagement of IL-12 receptor-expressing cells in the TME by endogenous IL-12 is important for inducing 4-1BB expression, which is necessary for an effective response to 4-1BBL-based therapy. In this context, endogenous IL-12 may enhance the immunostimulatory function of tumor-associated DCs, allowing them to overcome the suppressive tumor environment and efficiently prime CD8^POS^ T cells. These primed CD8^POS^ T cells can then be further co-stimulated through 4-1BB signaling, leading to improved polarization and effector function.

Taken together, these findings suggest that enhancing local expression of the co-stimulatory ligand 4-1BBL can boost the cytotoxic activity of CD8^POS^ T cells during rIL-12 treatment, even under the low endogenous IL-12 conditions typically observed in GB patients.

### rIL-12 administration combined with AAVF vector-mediated delivery of 4-1BBL in GFAP^POS^ cells increases survival in GB-bearing mice

**Figure 7:**
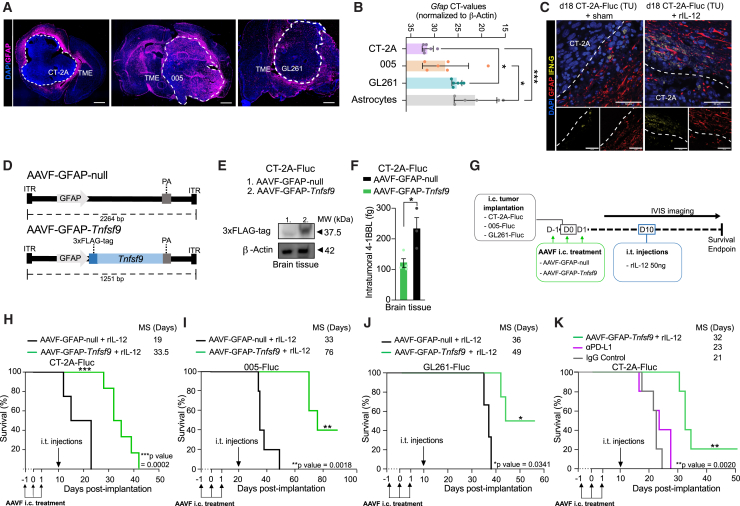
rIL-12 administration combined with AAVF-mediated delivery of *Tnfsf9* in GFAP^POS^ cells as a therapeutic intervention (A) GFAP^POS^ cell expression profile in the TME is dependent on the implanted mouse GB cell line. Immunofluorescence showing GFAP^POS^ astrocytes at the tumor border (whited dashed line) of CT-2A (left), 005 (middle), and GL261 (right) tumors 18 days post-implantation. With 005 and GL261 tumors, the GFAP^POS^ cells were retrieved in the brain tumor cell mass (4× magnification). Scale bar, 5 μm. (B) Endogenous GFAP expression in mouse GB cell lines and astrocytes. qRT-PCR analysis measuring *Gfap* expression levels for GB cell lines, CT-2A, 005, and GL261 and primary brain-derived astrocytes (*n* = 6/condition). Data represent three independent experiments and are presented as the mean ± SEM (error bars). Data were analyzed using one-way ANOVA, ∗*p* < 0.05, ∗∗∗*p* < 0.001. (C) CD8^POS^ T cells accumulate in proximity to GFAP^POS^ astrocytes in the peritumoral region following rIL-12 treatment. Representative immunofluorescence images of brain sections from CT-2A-FLuc tumor-bearing GREAT mice^109^ harvested on day 18 post-implantation. Sections show staining for GFAP (magenta, marking astrocytes), DAPI (blue, nuclear counterstain), and IFN-γ (white, marking activated immune cells). The left panel displays a tumor section from a mouse treated i.t. with sham (50 ng Fc), while the right panel shows a section from a mouse i.t. treated with 50 ng rIL-12 on day 10 post-tumor implantation. An increased number of IFN-γ^*POS*^ cells are observed in the rIL-12-treated condition, especially at the tumor border, marked by dashed white lines. Notably, IFN-γ^*POS*^ immune cells—likely CD8^*POS*^ T cells—are seen in close proximity to GFAP^*POS*^ astrocytes. Scale bars, 50 μm. (D) AAVF vector constructs to deliver *Tnfsf9* to tumor site*.* Schematic representation of AAVF-GFAP-*Tnfsf9* (encoding for 4-1BBL) and AAVF-GFAP-null (control) constructs. 3xFLAG-tag is fused to *Tnfsf9* and under a GFAP promotor with a poly(A) signal after the coding sequence. In the control AAVF-GFAP-null, the GFAP promoter and the poly(A) signal were connected without a coding sequence. (E) 4-1BBL protein expression at tumor site. 3xFLAG-tag protein was only detected in CT-2A-implanted mice brains (not treated with rIL-12) at day 18 post-implantation, injected with AAVF-GFAP-*Tnfsf9* (37.5 kDa) as normalized to β-actin (42 kDa) by western blot analysis. No fragmentation of the transgenic product was observed. (F) 4-1BBL protein levels in GB-containing brain hemisphere. 4-1BBL levels were determined in femtogram (fg) using Luminex in protein lysates from GB-bearing (CT-2A-FLuc) mice of both rIL-12- and sham-treated mice, collected at day 18 post-tumor implantation. Data represent three independent experiments and are presented as the mean ± SEM (error bars). Unpaired t test, ∗*p* < 0.05. (G) Graphic depiction of the treatment scheme of AAVF-GFAP-*Tnfsf9* experiments. *Tnfsf9*-coding or AAVF-null vectors were injected i.t. at three time points; 1 day prior to tumor implantation (CT-2A-FLuc, 005-FLuc, and GL261-FLuc), at the time of tumor implantation, and 1 day post-tumor implantation. rIL-12 was injected i.t. on day 10 post-implantation at the tumor site, and mice were followed by IVIS every 3–4 days. (H) Survival benefit with AAV-mediated delivery of *Tnfsf9* in rIL-12-treated CT-2A-FLuc-bearing mice. Kaplan-Meier curves displaying the percentage of survival of CT-2A-FLuc-bearing mice (12,500 cells at the time of injection) comparing AAVF-GFAP-*Tnfsf9* (green) and AAVF-GFAP-null (black) vectors both treated with rIL-12 (*n* = 4–6 mice per group). AAVF-GFAP-*Tnfsf9*- and rIL-12-treated mice had a median survival of 33.5 days (*p* = 0.0002) compared with AAVF-GFAP-null with a median survival of 19 days. Data represent at least two independent experiments and are presented as the mean ± SEM (error bars). Data were analyzed using the log rank (Mantel-Cox) test, ∗∗∗*p* < 0.001. Median survival in days (MS). (I) Recovery of survival benefit with AAV-mediated delivery of *Tnfsf9* into delayed rIL-12 treatment of 005-FLuc-bearing mice. Kaplan-Meier curves displaying the percentage of survival of 005-FLuc-bearing mice (50,000 cells at the time of injection) comparing AAVF-GFAP-*Tnfsf9* (green) and AAVF-GFAP-null (black) vectors both treated with rIL-12 on day 20 post-tumor implantation (*n* = 5 mice per group). AAVF-GFAP-*Tnfsf9*-rIL-12-treated mice had a median survival of >60 days (*p* = 0.0018) compared to AAVF-GFAP-null with a median survival of 33 days. Data represent at least two independent experiments and are presented as the mean ± SEM (error bars). Data were analyzed using log rank (Mantel-Cox) test, ∗∗*p* < 0.01. Median survival in days (MS). (J) Survival benefit with AAV-mediated delivery of *Tnfsf9* in rIL-12-treated GL261-FLuc-bearing mice. Kaplan-Meier curves displaying the percentage of survival of GL261-FLuc-bearing mice (50,000 cells at the time of injection) comparing AAVF-GFAP-*Tnfsf9* (green) and AAVF-GFAP-null (black) vectors both i.t. treated with rIL-12 on day 10 post-tumor implantation (*n* = 4 mice per group). AAVF-GFAP-*Tnfsf9* rIL-12-treated mice had a median survival of 49 days (*p* = 0.0341) compared with AAVF-GFAP-null with a median survival of 36 days. Data represent at least two independent experiments and are presented as the mean ± SEM (error bars). Data were analyzed using the log rank (Mantel-Cox) test, ∗*p* < 0.05. Median survival in days (MS). (K) The survival advantage of mice treated with AAVF-GFAP*-Tnfsf9* and rIL-12 compared with αPD-L1 therapy in CT-2A-FLuc-bearing mice. Kaplan-Meier curves show the percentage of survival of CT-2A-FLuc-bearing mice (50,000 cells at the time of injection). AAVF-GFAP-*Tnfsf9* injected on days −1, 0, and 1 and rIL-12 i.t. treated on day 10 post-tumor implantation (green) with αPD-L1 (pink) and IgG control (gray) (*n* = 5 mice per group). αPD-L1 and IgG control groups were treated i.p. with 200 μg in 100 μL volume on days 3, 5, and 14 post-tumor implantation. AAVF-GFAP-*Tnfsf9*- and rIL-12-treated mice had a median survival of 32 days, significantly improved (*p* = 0.0020) compared with the median survivals of mice treated with αPD-L1 (23 days) and IgG control (21 days). Data represent one independent experiment and are presented as the mean ± SEM (error bars). Data were analyzed using the log rank (Mantel-Cox) test, ∗∗*p* < 0.01. Median survival in days (MS).

Here, we tested whether a therapeutically relevant AAV vector-based gene therapy approach could effectively deliver *Tnfsf9* (encoding 4-1BBL) to enhance IL-12-mediated CD8^POS^ T cell activity. We focused on targeting GFAP^POS^ cells, as the GFAP marker is strongly present at the tumor site in GB patients.^69^ Elevated expression of GFAP has been reported in both malignant cells, predominantly in astrocyte-like cells,^70^^,^^71^ and in reactive astrocytes around the tumor (Figures S8A and S8B). To assess varying levels of GFAP expression in both the tumor and peritumoral regions—as seen in patients—we applied our strategy across three GB mouse models, which themselves exhibit distinct patterns of GFAP expression (Figures 7A and S8C). Notably, while our tumor cell lines express *Gfap*, astrocytes exhibit significantly higher expression potential (Figures 7B and S8D). Moreover, we anticipate that non-dividing or slowly dividing GFAP^POS^ astrocytes in the peritumoral regions are particularly well-suited for this strategy, as they are likely to support more sustained AAV-vector–mediated transgene expression over time. To demonstrate targeting of host-derived GFAP^POS^ cells, we injected AAVF-GFAP-GFP i.c. into the brain of a non-tumor-bearing mouse brain, resulting in selective expression in GFAP^POS^ cells (Figure S8E). We also confirmed that GFAP^POS^ cells in the TME are a preferred target for our strategy as they are capable of interacting with CD8^POS^ T cells (Figure 7C). Specifically, IFN-γ^POS^ cells—indicative of activated NK/T cells—were found in close proximity to GFAP^POS^ astrocytes in the peritumoral region of CT-2A tumors, particularly along the tumor border. This spatial association was observed in both sham- and rIL-12-treated mice; however, the rIL-12-treated group showed a higher number of activated IFN-γ^POS^ cells, consistent with the expected increase in inflammation under pro-inflammatory conditions.

AAVF vector constructs with a *GFAP* promoter were designed to express 4-1BBL-3xFLAG-tag (AAVF-GFAP-*Tnfsf9*) compared with a control vector, lacking the transgene (AAVF-GFAP*-*null) (Figure 7D). These cassettes were packaged into an AAVF capsid, selected for its robust transduction of astrocytes in the peritumoral region.^72^ To validate full-length recombinant 4-1BBL in i.c. injected AAV vector-treated mice with CT-2A-FLuc tumors, western blot analysis was performed with an anti-FLAG-tag antibody (Figure 7E). We confirmed that 37.5-kDa 4-1BBL was expressed in AAVF-GFAP-*Tnfsf9*-treated tumor brain samples and was not present in the AAVF-GFAP-null condition. Additionally, the concentration of i.t. 4-1BBL was measured with Luminex and showed 45% increased levels in the tumor hemisphere of mice treated with AAVF-GFAP-*Tnfsf9* compared with AAVF-GFAP-null (Figure 7F).

AAVF-GFAP-*Tnfsf9* and AAVF-GFAP-null were tested in mice i.c. engrafted with three different syngeneic GB cell lines (CT-2A-FLuc, 005-FLuc, and GL261-FLuc) and treated with rIL-12 (Figure 7G). The treatment strategy involved three i.c. injections of AAVF vectors over 3 days, within a time frame that would not trigger anti-AAVF immunogenicity^73^ but still guaranteed sufficient *Tnfsf9* expression at the tumor site. These GB cell lines have different growth rates and survival profiles when implanted in mice (Figures 1C, 1E, 1F, S2D, S2H, and S2I). To have comparable tumor size among models, rIL-12 treatment was given at around half the expected survival time post-tumor implantation. CT-2A-FLuc and GL261-FLuc were treated on day 10 post-implantation and 005-FLuc cells on day 20. Interestingly, despite comparable BLI signals at these time points between the CT-2A and 005 models (days 10 and 20, respectively), delaying the i.t. rIL-12 injection in the 005 model rendered it less susceptible to rIL-12—likely because the window for effective immune modulation had passed (Figure S9A). This suggests that, once tumor progression reaches a certain threshold, even immunostimulatory interventions like rIL-12 may no longer be sufficient to overcome established tumor growth, particularly in slow-growing models like 005. Nonetheless, in these three murine GB models, AAVF-GFAP-*Tnfsf9* treatment combined with rIL-12 administration improved median survival compared with AAVF-GFAP*-*null combined with rIL-12 (Figures 7H–7J and S9B–S9D). A 14.5-day survival benefit was observed in the CT-2A-FLuc model. For mice implanted with 005-FLuc and GL261-FLuc, 43- and 13-day survival benefits were observed, respectively. Importantly, we also tested our AAVF-GFAP-*Tnfsf9* and AAVF-GFAP-null vectors to demonstrate that *Tnfsf9* monotherapy can effectively overcome the lack of rIL-12 responsiveness observed in the 005 model when treated at day 20 (Figure S9E). Notably, these findings closely resemble those in Figure S7B, where engineered *Tnfsf9*-expressing tumor cells in *Il12*^*+/+*^ mice showed a therapeutic benefit without rIL-12—likely because CD8 T cell priming and subsequent 4-1BB expression occurred during tumor engraftment. The improved survival advantage of our combination treatment for GL261 and 005 GB models compared with the CT-2A model could be due to the lower number of activated astrocytes at the CT-2A border and the lower *gfap* expression of the CT-2A cells (Figure 7A). Indeed, transgene expression—detected via the 3xFLAG-tag—was primarily observed in GFAP^POS^ cells at the tumor border (indicated by the white dotted line) following AAVF-GFAP-*Tnfsf9* treatment. This expression was absent in the AAVF-GFAP-null condition (Figures S9F and S9G), even though tumor cells could be transduced by both AAVF-GFAP-*Tnfsf9* and AAVF-GFAP-null vectors *in vitro* (Figure S9H).

We compared the AAVF-GFAP-*Tnfsf9* and rIL-12 co-therapy to another immunomodulatory approach, anti-PD-L1 treatment (Figures 7K and S9I). Following a previously reported regimen,^74^ 200 μg anti-PD-L1 or IgG control were administered intraperitoneally (i.p.) on days 7 and 14 post-CT-2A-FLuc implantation. AAVF-GFAP-*Tnfsf9* and rIL-12 treatment prolonged the median survival by 9 and 11 days compared with mice treated with either anti-PD-L1 or IgG control, respectively. This represents an improvement of ∼40% over anti-PD-L1 monotherapy, showing the advantage of this gene therapy approach with rIL-12. Liver markers, including albumin, ALT, and ALP, showed no significant differences between mice administered with AAVF-GFAP-*Tnfsf9*, IgG control, and anti-PD-L1, indicating minimal gene therapy-induced detectable systemic toxicity (Figure S9J).

These results demonstrate that combining rIL-12 with gene therapy delivery of the co-stimulatory factor 4-1BBL prolongs the survival rate of GB-bearing mice more effectively than anti-PD-L1 therapy.

## Discussion

**Figure 8:**
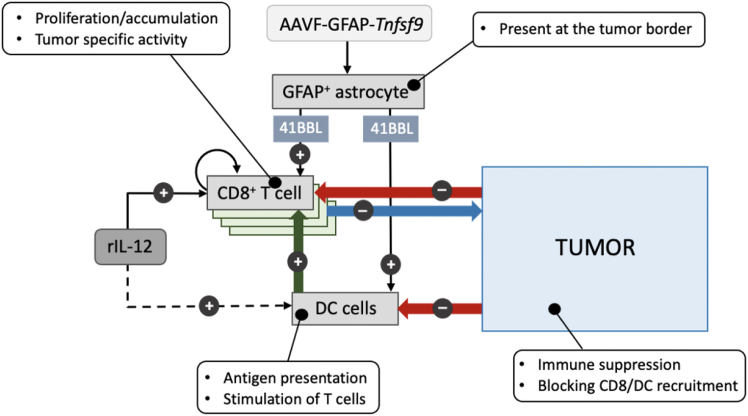
Schematic overview of rIL-12 and *Tnfsf9* co-therapy to enhance anti-GB immunity The diagram illustrates the proposed mechanism by which localized delivery of rIL-12 and AAVF vector-driven expression of 4-1BBL (encoded by *Tnfsf9*) in GFAP^POS^ astrocytes cooperatively enhance CD8^POS^ T cell-mediated anti-tumor responses. Intratumoral administration of rIL-12 stimulates both CD8^POS^ T cells and DCs, promoting 4-1BB expression next to activation, proliferation, and accumulation of T cells and DCs. AAVF-GFAP-*Tnfsf9* drives local 4-1BBL expression specifically in peritumoral astrocytes, providing additional co-stimulatory signals to infiltrating CD8^POS^ T cells and a subset of DCs (i.e., CCR7^POS^ DCs) that express 4-1BB. Moreover, activated DCs contribute to Ag presentation, cytokine production, and co-stimulation to further T cell stimulation. Together, these signals increase T cell effector activity and infiltration into the tumor resulting in tumor regression and the increase of survival of GB-bearing mice. The TME and tumor cells, characterized by immunosuppression and restricted immune cell recruitment, counteracts these effects. However, dual rIL-12 and 4-1BBL signaling synergizes to overcome these suppressive mechanisms, enhancing tumor-specific CD8^POS^ T cell activity at the tumor border and promoting anti-tumor immunity.

Tumor-reactive CD8^POS^ T cells are both rare and often dysfunctional in GB tumors.^26^^,^^27^^,^^28^ Efforts to restore their functionality have largely been unsuccessful to date.^21^^,^^75^ The accumulation and activity of CD8^POS^ T cells, which are essential for effective tumor clearance, are tightly regulated to minimize damage to healthy tissues by balancing stimulatory signals through the TCR, cytokines and co-stimulatory receptors with inhibitory signals through immune checkpoint and metabolite CTL receptors.^32^ To counteract the inhibitory and immunosuppressive signaling dominant in GB and shift the balance in favor of activating signals in the TME, we augmented the pro-inflammatory cytokine rIL-12 and the co-stimulatory factor 4-1BBL. Although both factors are well-described in oncology, little is known about their effect on GB. Historically the initial enthusiasm for pro-inflammatory agents such as IL-12 has declined due to its association with dose-dependent systemic toxicity in animals^76^ and clinical trials.^21^^,^^77^ Recently, alternative approaches for safely administrating therapy with pro-inflammatory stimuli have shown potential, including spatially controlled delivery and short-term administration.^78^^,^^79^^,^^80^ Therefore, we focused on administering a single low dose of soluble rIL-12 at the tumor site and utilizing AAV vectors to locally display 4-1BBL and improved survival in preclinical GB mouse models. A schematic summary of the proposed hypothesis illustrates how *Tnfsf9* treatment enhances rIL-12 therapy by promoting interactions among distinct immune cell populations, stimulatory ligands, and receptors at the tumor site (Figure 8).

Tumor-associated CD8^POS^ T cell responses are regulated by intratumoral DCs. DCs convert information from their surroundings into tailored instructions to guide T cell responses.^81^ DC-derived signals that drive T cell proliferation and activation include: (1) Ag peptide-mediated presentation by MHC molecules (e.g., MHC-I and MHC-II), (2) co-stimulatory ligands (e.g., CD80, CD86, and 4-1BBL), and (3) cytokines (e.g., IL-12 and type I IFNs).^32^^,^^42^^,^^82^^,^^83^ However, in GB, DC function and numbers are suppressed compromising anti-tumor immunity.^30^^,^^84^ We evaluated whether the lack of anti-tumor reactivity was due to insufficient DC instructions, dysfunction of CD8^POS^ T cells, or both. This was investigated using isolated murine GB-associated CD8^POS^ T cells from mouse brains. Tumor-infiltrating CD8^POS^ T cells retained the ability to secrete IFN-γ following MHC-I presentation of the tumor neoantigen, mImp3 by naive DCs,^43^ indicating the presence of a tumor-Ag-specific TCR. However, this response was only achieved when the CD8^POS^ T cells were stimulated with rIL-12, suggesting that the engagement between DCs and CD8^POS^ T cells necessary for activation is insufficient in the GB TME.

We then explored which functional DC states provide the necessary Ag cross-presentation and immunomodulatory signals to CD8^POS^ T cells in GB. By processing and analyzing of available scRNA-seq datasets we deduced that a discrete *Ccr7*^POS^ DC state in GBs expressed *Il12b* and high levels of MHC-I components, along with high levels of co-stimulatory molecules and pro-inflammatory cytokines essential for CD8^POS^ T cell activation. The CCR7 signature suggested that these DCs are migratory,^47^ involved in capturing tumor-associated Ags within the TME and subsequently trafficking to the draining lymph nodes to present Ags to CD8 T cells.^82^^,^^85^ The tumor-retained DCs referred to here as *Ccr7*^POS^ DCs arise from cDC precursor cells and have different names in other tumor types, including DC3, migratory DCs, mRegDCs, LAMP3^POS^ DCs, mature DCs, or activated DCs.^48^^,^^86^ Here, we found that, in addition to their IL-12 production potential,^87^ other key immune modulators were expressed by these DCs, such as co-stimulatory IL15/RA, CXCL16, 4-1BBL, CD80, and inhibitory PD-L1/2.^88^ Interestingly, the receptors for IL-12 and 4-1BBL products were also intrinsically expressed in this DC state, suggesting that there might be a positive feedback loop to enhance their activation and thereby promote a more robust anti-tumor immune response by CD8^POS^ T cells. Indeed, when exogenous rIL-12 was supplied to the tumor, it induced *Il12b*^POS^ DC accumulation and STAT4p, suggesting that *Ccr7*^*POS*^ DCs responded to rIL-12, directly or indirectly through other cells. This has been reported in other studies where IL-12R in DCs stimulates autocrine signaling to maintain IL-12 expression.^89^^,^^90^^,^^91^ A similar effect can be envisioned for exogenous 4-1BBL expression and 4-1BB receptor-expressing *Ccr7*^POS^ DCs.^92^ The underperformance of *Ccr7*^POS^ DCs in GB, despite their presence in scRNA-seq datasets, warrants further investigation. Our data suggest that these tumor-residing DCs are rare in mouse GB and even more limited in *de novo*/recurrent glioma patient datasets. Next to their limited presence compared with other types of cancer, such as melanoma, *Ccr7*^POS^ DCs also express high PD-L1 levels when they are activated, imposing suppression on CD8^POS^ T cells. These observations, together with the possibility that both DCs and CD8^POS^ T cells are not abundant enough to contribute effectively to the anti-tumor immune response, likely explain why levels of rIL-12 or 4-1BBL in GB do not affect patient survival. Therefore, survival data may not reflect the potential effect of supplementing exogenous IL-12 or 4-1BBL, which could provide the necessary signals to activate immune pathways.

Besides affecting DCs, IL-12 and 4-1BBL have been suggested to activate CD8^POS^ T cells,^93^ resulting in the release of cytotoxic molecules, such as granzymes,^94^ and can also support other immune cells, such as NK cells^95^^,^^96^ and regulatory/helper cells.^97^ Analyzing human and murine scRNA-seq datasets, we confirmed the expression of IL12Rβ1/2 and 4-1BB in NK and regulatory T cells and, with flow cytometry, we demonstrated that they phosphorylate STAT4 upon rIL-12 treatment. Nonetheless, antibody-based depletion of CD8 T cells, CD4 T cells, and NK cells in our GB models illustrated that CD8^POS^ T cells are primarily responsible for the rIL-12-induced immunity. This aligns with findings in tumor models in transgenic mice, where it was demonstrated that, although NK and CD4 T cells can have a supportive function, they are less likely to be directly involved in IL-12-mediated anti-tumor immunity.^98^^,^^99^ In the context of human glioma, the VDX trials showed that, besides the IFN-γ signature, predominantly the number of CD8 T cells, and not CD4 T cells, increased at the tumor site.^21^ Upon local rIL-12 exposure of the GB, the recruited CD8^POS^ T cells differentiated toward a more effector-like state. This state exhibited high levels of PD-1, GZM-B, and 4-1BB and increased STAT-4p. Overall, our data suggest that CD8^POS^ T cells are directly affected by rIL-12, and their activity is indirectly supported by *Ccr7*^POS^ DCs.

Given that CD8^POS^ T cells at the tumor site express 4-1BB, IL-12Rβ1/2, and tumor-specific Ags, their activation is likely limited by the scarcity or immunosuppressed state of *Ccr7*^POS^ DCs. To overcome this limitation, we investigated an alternative model that delivers stimulatory signals through mechanisms independent of DCs. We demonstrated that 4-1BBL overexpression at the tumor site increased the survival of rIL-12-treated animals, and we could recapitulate these results with AAV vector-mediated *Tnfsf9* gene therapy. The combination of *Tnfsf9* overexpression by tumor cells and rIL-12 nearly doubled (∼70% versus 40%, respectively) the proportion of long-term survivors compared with *Tnfsf9* overexpression alone. These findings suggest that *Tnfsf9* contributes to therapeutic efficacy or local rIL-12 treatment. Other therapeutic attempts with a combination of IL-12 and 4-1BBL have, to our knowledge, only been attempted with other cancers, such as colon carcinoma,^100^ liver metastasis,^101^ and melanoma.^102^

In contrast to tumoricidal drugs that act on tumor cells directly, AAV vector-based immunotherapy can also act in the vicinity of the tumor and the transgene products can work indirectly, e.g., through CD8^POS^ T cells. Recently, a successful AAV vector therapy approach has been deployed targeting the cytokine LIGHT in endothelial cells in the vasculature of GB with reduced CD8^POS^ T cells.^19^ In our strategy, 4-1BB^POS^ CD8^POS^ T cells recruited by rIL-12 were stimulated by AAV vector-mediated delivery of 4-1BBL. 4-1BB stimulation with agonist antibodies has shown promising effects on CD8^POS^ T cells in patients with advanced solid tumors,^103^ B cell lymphoma,^104^ or pancreatic cancer.^105^ Our strategy created a continuous reservoir of 4-1BB stimulation at the tumor site. This was achieved by packaging the AAV vector with an astrocyte-tropic AAVF capsid and using a GFAP promoter to drive the transgene, with GFAP being elevated in slow-dividing reactive astrocytes associated with the tumors^72^^,^^106^^,^^107^ and, to a certain extent, in proliferating tumor cells. Reduced expression in tumor cells is likely due to their proliferation contributing to AAV vector genome loss.^108^ GB models with higher GFAP expression in and around the tumor, such as GL261 and 005, were more susceptible to this therapeutic strategy than CT-2A, which exhibit lower GFAP expression. AAV vector was injected into the tumor cavity over 3 consecutive days to achieve sufficient therapeutic transgene expression, while avoiding immune-mediated elimination of the AAVF capsid. Local expression of *Tnfsf9* via AAVF vector-mediated delivery enhanced rIL-12-driven immunity and prolonged overall survival across all tested GB models. Notably, in the 005 model, mice treated with repeated AAVF injections alone showed a significant survival benefit, suggesting potential for clinical translation. However, this effect was not observed with a single dose of AAVF (data not shown), highlighting the necessity for dual therapy. Importantly, our combination therapeutic approach was shown to be more effective than ICI, such as anti-PD-L1. Our therapeutic strategy models the intraoperative administration of AAVF therapy directly into the resected tumor cavity via i.t. injection. For clinical translation, this protocol would require adaptation to enable practical and effective delivery methods. One potential approach could involve administering rIL-12 during tumor resection, combined with the implantation of a reservoir or depot system for sustained, localized release of AAVF vectors postoperatively. Such a system could facilitate prolonged vector expression at the resection site, potentially enhancing therapeutic efficacy. Prior to clinical implementation, rigorous assessment of safety, efficacy, and adherence to regulatory requirements will be necessary.

In conclusion, we have demonstrated an immuno-gene therapy that led to increased survival in GB-bearing mice. These findings potentially offer improved outcomes to GB patients compared with ICI therapies.

## Methods

### Experimental model and subject details

#### Animals

All animal experiments were performed in agreement with ethical guidelines of the National Institutes of Health for the Care and Use of Laboratory Animals. Experiments were conducted under the oversight of the Massachusetts General Hospital Institution Animal Care and Use Committee (IACUC). C57BL/6J were purchased from Charles River Labs (IACUC protocol 2009N000054). T.R.M. provided *Il12b*^*tm1.1Lky*^*/J* (IL-12 p40-YFP) mice (JAX: no. 006412)^51^ and the B6.129S1-*Il12b*^*tm1Jm*^/J (IL-12p40 KO) mice (JAX: no. 002693).^36^ C.129S4(B6)-*Ifng*^*tm3.1Lky*^/J (GREAT) mice (JAX: no. 017580)^109^ were crossed at least 11 times with the C57BL/6 mice. The GREAT mice were crossed and obtained from Dr. Chris Garris’s Laboratory. Animals were maintained in specific pathogen-free facilities at Massachusetts General Hospital (MGH) with unlimited access to water and food under a 12-h light/dark cycle. To study the immunomodulatory effects of rIL-12 and 4-1BBL on GB *in vivo*, C57BL/6J adult male and female mice were randomly assigned to each group.

#### *In vivo* bioluminescence analysis

*In vivo* tumor growth in brains was monitored by FLuc by BLI using a Xenogen *in vivo* 200 Imaging System (IVIS) (PerkinElmer). D-Luciferin (Gold Biotechnology) was reconstituted by adding 50 mL of 1× sterile phosphate-buffered saline (PBS) to the lyophilized pellet. Working solution (100 μL) was injected i.p. in mice. Imaging was acquired 5 min after injection and analysis was performed using Living Image software 4.3.1 (PerkinElmer).

#### Cell culture

The National Cancer Institute (NCI) provided mouse GB cells (CT-2A, GL261, and 005) syngeneic with strain C57BL/6J. HEK293T cells were purchased from ATCC. Cells were cultured at 37°C in a 5% CO_2_ humidified incubator. CT-2A cells were cultured in Dulbecco’s modified Eagle’s medium (DMEM) (Corning) supplemented with penicillin (100 units/mL) and streptomycin (100 mg/mL) (P/S) (Corning) and 10% fetal bovine serum (FBS) (Gemini Bioproducts, West Sacramento, CA). GL261 cells were cultured in Roswell Park Memorial Institute (RPMI) (Corning) with 10% FBS and 1% P/S. 005 cells were cultured in DMEM Nutrient Mixture F-12 (DMEM/F-12, Gibco Thermo Fisher Scientific). DMEM/F-12 was supplemented with 1% P/S, B-27 supplement (1×, Gibco Thermo Fisher Scientific), heparin (Sigma-Aldrich) (2 μg/mL), epidermal growth factor (R&D system) (20 ng/mL), and fibroblast growth factor (PeproTech) (20 ng/mL). Cells tested negative for mycoplasma contamination at periodic intervals throughout the study (Mycoplasma PCR Detection Kit G238; ABM, Richmond, BC, Canada).

For *in vivo* experiments, GB cells (CT-2A, GL261, and 005) cells were stably transduced with an LVV vector to express FLuc (Addgene no. 108542) and were used for all subsequent *in vivo* experiments.

The FLuc plasmid was obtained from Addgene (catalog no. 108542) and was transfected into HEK293T cells along with capsids and packaging material for lentivirus production. HEK293T cells were cultured for 24 h and fresh medium was provided 72 h after transfection for lentivirus production. The conditioned medium was spun down at 300 × *g* for cell debris removal. The supernatant was filtered through a 0.2-μm filter and the virus was pelleted at 330,000 × *g* for 2 h. All virus preparations were aliquoted and kept frozen at −80° until use. The FLuc virus was transduced into CT-2A, GL261, and 005 cells and transduced cells were selected with blasticidin.

To study the effect of 4-1BBL, GB cells (CT-2A-FLuc and 005-FLuc) were stably transduced with an LVV to express 4-1BBL under the GFAP promoter-tagged 3xFLAG-tag and with fluorescent label mCherry or the control vector lacking 4-1BBL.

### Method details

#### I.c. tumor implantation

Adult mice were anesthetized using 2.5% isoflurane (USP, Baxter Healthcare Corporation) in 100% oxygen via a nose cone and placed on a warm pad to avoid hypothermia. A total of 5 × 10^4^ CT-2A-FLuc was suspended in 1 μL Opti-MEM (Gibco, Waltham, MA). In total, 2 μL of the cell suspension was then implanted into the left striatum of C57BL/6J mice, IL-12 p40-YFP mice, or IL-12p40 KO (*Il12*^−/−^) mice using a Hamilton syringe (Sigma-Aldrich, Germany) and automatic stereotaxic injector (Stoelting, Wood Dale, IL) with a flow rate of 0.2 μL/min for 10 min. In reference to bregma, three coordinates for stereotactic implantation were chosen: anterior-posterior (AP) = 2.0 mm, medial-lateral = 0.5 mm, and dorsal-ventral = 2.5 mm. Overall survival of the mice was based on 20% weight loss, presence of apparent distress, or actual death. Tumor growth in mice was assessed by measuring BLI using IVIS (PerkinElmer, Waltham, MA) every 3 or 4 days starting from day 7 after tumor implantation.

#### IL-12 and AAVF vector treatment

For CT-2A-FLuc tumors, 10 days after i.c. injection, mice were treated with 5, 20, 50, 200, or 500 ng rIL-12-FC (Adipogen; catalog no. CHI-MF-11112-C025) or FC (Adipogen; catalog no. CHI-HF-210IG1-C100) (50 ng) sham control by i.t. injections using a Hamilton syringe (Sigma-Aldrich) and an automatic stereotaxic injector (Stoelting) with a flow rate of 0.2 μL/min for 10 min at the coordinates used for tumor implantations. For GL261-FLuc and 005-FLuc tumors, 50 ng rIL-12 or sham was injected i.t. at day 10 post-tumor implantation.

For three mouse GB models (CT-2A, GL261, and 005) AAVF vectors (AAVF-*Tnfsf9* and AAVF-null) were i.c. injected in a volume of 5 μL (5.0 × 10^13^ genome copies [gc]/mL at three time points. One day prior to tumor implantation (day −1), on day 0 (tumor implantation), and 1 day post-tumor implantation (day 1) the mice were injected with the AAVF vector using a Hamilton syringe (Sigma-Aldrich,) and an automatic stereotaxic injector (Stoelting) with a flow rate of 0.2 μL/min for 10 min. In reference to bregma, three coordinates for stereotactic implantation were chosen: AP = 2.0 mm, medial-lateral = 0.5 mm, and dorsal-ventral = 2.5 mm. The same coordinates were used for all three AAVF vector injections.

#### Depletion of CD8 cells, CD4 T cells, or NK1.1 cells

To deplete CD8 T cells, CD4 T cells, or NK1.1 cells in C57BL/6J and *Il12*^−/−^mice, endogenous CD8, CD4 T, or NK1.1 ells were depleted by i.v. injection of anti-mouse CD8β antibody (Bioxcell, Clone Lyt 3.2) anti-mouse CD4 antibody (Bioxcell, Clone GK1.5), anti-mouse NK1.1 antibody (Bioxcell, Clone PK136), or rat IgG2b isotype control (Bioxcell, Clone LTF-2) on day 9 (50 μg) and day 10 (100 μg) post-tumor implantation. On day 10, rIL-12 or sham was injected i.t. at the tumor site and mice were sacrificed on day 18 for flow cytometry of dissociated brain cells.

#### Anti-PD1/PD-L1

CT-2A tumor-bearing mice were administered with i.p. injections at days 3, 5, and 14, with anti-PD-L1 (Leinco Technologies, clone 10F.9G2) or with rat IgG2b isotype control (Bioxcell, clone LTF-2) at a dose of 200 μg/mouse in a volume of 100 μL. Tumor growth was measured by IVIS every 3–4 days and mice were euthanized based on 20% weight loss, presence of apparent distress, or actual death.

Whole blood (∼400 μL) was retro-orbitally collected and was sent to the Pathology core on day 15 for pathology toxicology analysis.

#### Retro-orbital blood collection

To identify the depletion of CD8 T cells in the blood post-i.v. injection of anti-CD8 antibody, 100 μL retro-orbital blood was collected via 1.2-mm glass capillaries (World Precision Instruments) in EDTA tubes to avoid coagulation on days 7, 11, and 18 post-tumor implantation. The collected blood was further processed immediately for RNA isolation.

#### Whole-blood collection

Mice were sacrificed by a lethal 100 μL i.p. injection containing ketamine (5 μL), xylazine (45 μL), and saline (50 μL) (Patterson Veterinary). Upon ceasing of all reflexes, whole blood was collected directly from the heart in EDTA tubes. The blood was processed immediately by centrifugation at 1,500 × *g* for 15 min to pellet the blood cells. The blood cell pellet was washed with PBS carefully and used for RNA isolation to determine the expression of CD8 in whole-blood cells. The supernatant was carefully centrifuged again at 2,500 × *g* to collect the plasma. The plasma was further analyzed at the Pathology core at MGH, with the comprehensive blood toxicology panel.

#### AAV plasmid constructs and production

AAV-F in pAR-9 was a kind gift from Casey Maguire (Addgene, plasmid no. 166921; https://www.addgene.org/166921/; RRID: Addgene_166921).^106^ The 4-1BBL expression construct was cloned into a GFAP-GFP AAVF vector plasmid (AltaBiotech), using the restriction enzymes NheI-HF and NcoI-HF (New England Biolabs) followed by Gibson assembly with NEBuilder HiFi DNA Assembly Master Mix (New England Biolabs). Both the AAVF-*Tnfsf9* and AAVF-null vector plasmids were then transformed into SURE Electroporation Competent cells (Agilent Technologies) by two pulses at 1,700 V. Plasmid DNA was isolated in nuclease-free water (Ambion Life Technologies) using the Qiaprep Spin miniprep kit (QIAGEN) after selection with 1 μg/mL ampicillin (ampicillin sodium salt, Sigma). Both plasmid constructs were fully sequenced with next-generation sequencing at the MGH CCIB DNA core and analyzed with SnapGene software version 6.0.2. Upon confirmation of the sequence, the plasmid constructs were isolated at a large scale by AltaBiotech at a concentration of 2 μg/μL. Subsequently, scAAVF vectors were produced by Packgene at a titer of 1.0 × 10^13^ gc/mL.

#### Western blots

Total protein was extracted from cultured cells using RIPA lysis buffer (Thermo Scientific). The tissue samples were homogenized in RIPA lysis buffer with a tissue homogenizer. RIPA buffer was supplemented with a protease inhibitor cocktail (Sigma-Aldrich). To remove non-soluble cell debris, samples were sonicated using a probe sonicator (Sonic Dismembrator Model 100, Fisher Scientific) at a setting of 3.0 for 5 s and centrifuged at 15,000 × *g* for 10 min at 4°C. Protein concentration was determined using the Pierce BCA Protein Assay Kit (Thermo Fisher Scientific). Absorbance was measured at 562 nm using the SynergyHI microplate reader (BioTek). Equal amounts of protein (20 μg) mixed with Laemmli SDS-Sample buffer (Boston BioProducts) were loaded and resolved by electrophoresis on NuPage 4%–12% Bis-Tris polyacrylamide gels (Thermo Fisher Scientific) in NuPage MES SDS Running Buffer (Thermo Fisher Scientific). After transfer onto nitrocellulose membranes using the iBlot 2 (Thermo Fisher Scientific), samples were subsequently incubated for 1 h at room temperature (RT) in 5% non-fat dry milk (LabScientific) in Tris-buffered saline (pH 7.4) with 0.05% Tween 20 (TBS-T) and probed with primary antibody mouse 3xFLAG-tag 1:1,000 (Merck, F3165) or goat-α,β-actin (Santa Cruz Biotechnology, I-19) overnight at 4°C. After washing three times with TBS-T for 10 min, membranes were incubated for 1 h at RT with secondary antibodies ECL donkey-anti-goat immunoglobulin G (IgG) (Sigma-Aldrich) and ECL sheep-anti-mouse IgG (Thermo Fisher Scientific) (1:5,000) corresponding to the primary antibodies. Membranes were developed with ECL or Femto staining (Thermo Fisher Scientific) and imaged on an Azure Biosystems C300 gel imager.

#### Luminex

To quantify protein concentrations in C57BL/6J mice and IL-12p40 KO (*Il12*^−/−^) mice, frozen tumor-bearing brains of mice treated with sham or rIL-12. Brains were harvested at day 18 post-tumor implantation and frozen. Tumor tissue was cut out of the brain and cut in small pieces. Tumor tissue was weighed in microcentrifuge tubes and 100 μL of RIPA lysis buffer (Thermo Scientific) was added per 100 mg of tissue. Stainless steel 3-mm tungsten carbide beads (QIAGEN) were added to homogenize the tissue using the TissueLyser system (QIAGEN) for 3 min at 0.25 Hz speed. Samples were centrifuged at 16,000 × *g* for 10 min at 4°C. Supernatant were transferred to new microcentrifuge tubes. Protein concentration was determined using the Pierce BCA Protein Assay Kit (Thermo Fisher Scientific) and samples were diluted to 10 mg protein/mL with 1× PBS. To proceed with ProcartaPlex mouse basic kit (Invitrogen) protocol, 25 μL of Universal Assay Buffer (catalog no. EPX-11111-000) was added to 25 μL of the diluted sample per sample well. Samples were incubated with beads overnight and analyzed by flow cytometry to detect events in the PE channel.

#### qRT-PCR

Total RNA was extracted using the Direct-Zol RNAmini kit (Zymo-research). RNA concentrations were measured using the NanoDrop Spectrophotometer ND-1000 (Thermo Fisher Scientific). For gene expression analysis using qRT-PCR, cDNA was synthesized from 200 ng total RNA and prepared using the SuperScript Vilo cDNA Synthesis Kit (Thermo Fisher Scientific). cDNA samples were diluted 10-fold with nuclease-free water. Gene expression was determined using the manufacturing protocol of PowerUp SYBR Green PCR Master Mix (Applied Biosystems). The cycling conditions using the standard protocol were: 2 min at 50°C, 10 min at 95°C, 40 cycles of 95°C for 15 s, and 60°C for 1 min, followed by a melt curve from 60°C to 95°C at 0.1°C/s, with 15 s hold at 95°C. Twenty-five sets of primers (Table S5) obtained from Origene (https://www.origene.com/) were used to specifically target the genes of interest by qRT-PCR. Gene expression was normalized to the housekeeping mRNA β-actin.

#### Tissue digestion

The mice were exsanguinated further with PBS perfusion. A Tumor Tissue Dissociation Kit (Miltenyi Biotec) was used to process the brain into a single-cell suspension. Brains were placed into a GentleMacs C-tube (Miltenyi Biotec) with 2.35 mL RPMI 1640 (Corning) containing enzymes D (100 μL), R (30 μL), and A (3.5 μL). According to the manufacturer’s protocol, the brains were dissociated using the gentle MACS Dissociator (Miltenyi Biotec) on the brain program settings. Samples were run through a 70-μm filter to obtain a single-cell suspension. Myelin removal was achieved using magnetic separation and anti-myelin beads (Miltenyi Biotec). The final cell suspension was resuspended in 1× Dulbecco’s PBS without calcium (Ca^2+^) or magnesium (Mg^2+^) (Corning), supplemented with 2 mM EDTA (Thermo Fisher Scientific) and 0.5% BSA (Sigma). Samples were then loaded onto a series of LS columns containing microbeads conjugated to anti-mouse CD11b and anti-mouse CD45 (Miltenyi Biotec), respectively, and separated into CD11b^POS^, CD11b^NEG^CD45^POS^, and CD11b^NEG^CD45^NEG^ (non-immune) cell populations using the MACS multi-stand (Milteny Biotec).

#### Antibody staining and flow cytometry

Cell surface proteins were stained for 20 min at 4°C. Intracellular and nuclear proteins were stained for 60 min at RT after permeabilization and fixation (Thermo Fisher Scientific) for 30 min at RT. To investigate T cells, samples were stained with different antibodies (Table S6). Stained cell samples were resuspended in 200 μL FACS buffer (Dulbecco’s PBS supplemented with 2 mM EDTA and 0.5% FBS) and transferred to FACS tubes (Stellar Scientific). A mixture of isolated lymph nodes derived from the thigh and spleens were passed through 70-mm cell strainers, pellets were then incubated with red blood cell lysis buffer (Boston Bioproducts) two times for 5 min and washed with PBS. These lymph nodes and splenocyte mixtures were used as single-stained controls. In all experiments, lymph nodes, spleens, and ipsilateral hemispheres implanted with CT-2A cells were mixed to measure the fluorescence minus one (FMO). For all studies, dead cells were stained using the fixable viability violet dyes—Zombie Red or Zombie Blue (Invitrogen)—for 10 min at RT, followed by blocking of Fc receptors with TruStain fcX (BioLegend) for 15 min at 4°C. Cells were analyzed on LSRFortessa or LSRFortessa X-20 flow cytometers (BD Biosciences) and data were analyzed with FlowJosoftware version 10.8.1.

#### Immunohistochemistry

Whole brains from mice were fixed overnight in 4% paraformaldehyde at 4°C. The following day, brains were transferred to a 30% sucrose (Sigma) solution and incubated until they sank, indicating proper cryoprotection. Brains were then embedded in optimal cutting temperature compound (Fisher Scientific) and snap frozen. Serial coronal sections (12 μm thick) were prepared using a cryostat and mounted onto Fisherbrand microscope slides (Canada). The sections were fixed again with 4% paraformaldehyde for 10 min at RT, followed by three 5-min rinses in PBS. Blocking was performed for 1 h at RT in blocking buffer consisting of 5% goat serum and 0.1% Tween 20 in PBS (PBS-T). Brain slices were then incubated with the primary antibodies (GFP 1:400, Invitrogen, catalog no. A11120; GFAP 1:400, Invitrogen, catalog no. 13-0300; CD8 1:400, Novus Biologicals, catalog no. NBP2-29475; IL12Rb1 1:400, Invitrogen, catalog no. PA5-95976; anti-4-1BB 1:100, Absolute Antibody, catalog no. Ab01052; 3xFLAG-tag 1:400, Abcam, catalog no. ab245893; 4-1BBL 1:100, Invitrogen, catalog no. MA529838; CD11c 1:400, Abcam, catalog no. ab33483), diluted in blocking buffer at 4°C overnight. Slices were rinsed three times in PBS-T for 5 min each. Secondary antibodies (goat anti-rabbit 1:400 Invitrogen, catalog no. A11008; goat anti-rat 1:400 Abcam, catalog no. ab150157; 1:400 goat anti-mouse Invitrogen, catalog no. A11001) were diluted in PBS-T and incubated for 1 h in the dark at RT. Slices were mounted with DAPI (Vectashield, Vector Labs, San Francisco, CA).

#### H&E staining

For H&E staining, brain slices were air dried under a fan for 20 min, before fixation in 100% ethanol for 10 min. Brains were rinsed briefly in Milli-Q (EMD Millipore), then stained for 10 min at RT with Harris Hematoxylin (Poly Scientific R&D). Slides were washed twice with Milli-Q for 2 min, then de-stained in 1% acetic acid (Sigma-Aldrich) for 6 s, followed by washing twice in Milli-Q. Samples were differentiated in 0.05% aqueous lithium carbonate (Poly Scientific R&D) for 30 s, after which they were washed in warm tap water for 2 min 1% Eosin Y solution (Electron Microscopy Sciences) was pipetted on top of the sections to counterstain for 4 s. Next, brains were de-stained in 95% ethanol for 20 s, followed by further de-staining and dehydration in 100% ethanol for 5 min. Brain sections were cleared in xylene (Sigma-Aldrich) for 15 min, mounted with Permount (Electron Microscopy Sciences) and imaged on a Keyence microscope at 4× magnification.

#### IFN-γ Elispot assay

GL261 tumor-bearing mice were sacrificed on day 14 post-tumor implantation and the ipsilateral hemisphere was dissociated into single-cell suspensions. Tumor single-cell suspensions were separated from myelin using magnetic separation and anti-myelin beads (Miltenyi Biotec). The myelin-negative cell pellet was incubated with microbeads conjugated to anti-mouse CD45 (Miltenyi Biotec). After magnetic separation, the CD45-positive cell pellet was incubated with CD8 microbeads (Miltenyi Biotec) to isolate for positive CD8 T cells. CD8^POS^ T cells were cultured in RPMI 1640 (Corning) with 10% FBS and 1% P/S, 1% GlutaMAX (Gibco), and 0.01% 2-mercaptoethanol (Thermo Fisher Scientific) stimulated with IL-2 overnight. Splenocytes were derived from a spleen that was filtered in PBS through a 70-mm cell strainer followed by incubation with red blood cell lysis buffer (Boston BioProducts) two times for 10 min. CD8^POS^ T cells and splenocytes were counted. A total of 150,000 CD8^POS^ T cells combined with 25,000 splenocytes was plated in a 3:1 ratio either with or without mImp3, GL261-specific neopeptide (AALLNKLYA) together with either 50 ng FC or rIL-12 overnight in 200 μL RPMI at 37°C on a pre-coated murine IFN-γ detection plate (ImmunoSpot). After following manufacturers’ protocol, wells were dried overnight, and images were quantified by ImageJ.

#### Cell viability assay

Cell proliferation was assessed *in vitro* by the WST reduction assay to determine cell viability (cell counting kit-8; Dojindo, Rockville, MD) of FACS-sorted GFP^POS^ cells. Cells were seeded at a low density (2 × 10^3^ cells/well) in a 96-well plate. After 24 h, the medium was removed, and 10% WST solution was added to the cells. The cells were incubated at 37°C for 1 h, and absorbance levels at wavelength 450 nm were measured using a microplate reader (SynergyH1; BioTek, Winooski, VT). Thereafter, the medium was changed, and cells were measured repeatedly every 24 h up until 90% confluency on day 5.

#### scRNA-seq analysis

##### scRNA-seq datasets tumor-bearing mice/human samples enriched for immune cells

For the scRNA-seq analysis, publicly available datasets or datasets provided by co-authors were used (Table S2). The Seurat v4-v5 R package was used to preprocess and analyze the data.^110^^,^^111^ Unless otherwise stated, the Seurat Pipeline was followed. Low-quality cells were excluded from the analysis. The count matrix and cell metadata were used to create a Seurat object, of which the standard Seurat Pipeline was followed by running NormalizeData, FindVariableFeatures (using variance stabilizing transformation), ScaleData, RunPCA, FindNeighbors, FindClusters, and RunUMAP. Cell types were annotated using published cell annotation matrices^112^ and projected on the other datasets to homogenize analyses between datasets. To examine the expression levels of genes of interest, “VlnPlot,” “FeaturePlot,” and “AverageExpression” functions from Seurat were used. The proportion of cells per cluster that expressed genes of interest (normalized counts >0) was also calculated.

For feature plots, Uniform Manifold Approximation and Projection (UMAP) visualizations and heatmap analysis, we utilized a subset of the scRNA-seq data from our preprint Miller et al.^49^ specifically incorporating data derived from Johnson et al.^113^ and Abdelfattah et al.^114^ Cell annotations were applied according to Miller et al.^49^ to ensure consistent classification. The data were normalized to 10,000 counts per cell, log-transformed, and the top 3,000 most highly expressed genes were selected for dimensionality reduction and downstream analysis. Principal-component analysis was used for dimensionality reduction, and a nearest neighbors’ graph was constructed with standard parameters (n_pcs = 40, n_neighbors = 10). UMAP was subsequently applied for visualization. All analyses and visualizations were conducted in Python using the Numpy, Pandas, and Scanpy^114^ libraries.

The survival information (survival time) and events were obtained from the G-SAM^115^ and GLASS^116^ cohorts. IDH-WT GB were exclusively considered for this analysis. Duplicate patient entries were excluded, and the values were maintained from the primary tumor only for the patient. For genes, the CPM-normalized value was used. For gene sets, the CPM-normalized and log-transformed matrix was uploaded to Seurat, and Module scores of gene sets were calculated using the AddModuleScore() function. CIBERSORTx^117^ was used to normalize the expression of genes or module scores to the myeloid contents in the cohorts. Discretized matrix was utilized from Miller et al.^50^ as a reference matrix for CIBERSORTx.^118^ We removed any library with a CIBERSORTx value of 0 for the myeloid lineage. Samples in the top 33% in terms of expression of genes of interest (or module scores) were labeled as "high." The bottom 33% were considered the "low" group. We used ggsurvfit (https://github.com/pharmaverse/ggsurvfit) to generate the Kaplan-Meier survival curve. A Cox proportional hazard model (https://github.com/therneau/survival) was used to determine differences in survival probabilities.

### Quantification and statistical analysis

Bar graphs, heat maps, and survival plots were made in GraphPad Prism 9.5.1. Error bars show the mean ± SEM. A one-way ANOVA, two-way ANOVA, multiple t tests, and log rank tests were applied to determine if conditions significantly differed. Statistical significance was specified as *p* < 0.05. Sequences and plasmid constructs were analyzed with SnapGene software version 6.0.2.

## Data availability

The datasets analyzed and generated during this study are available from the corresponding author upon reasonable request. All R codes of the current project are available on GitHub and other custom scripts for analyzing data are available upon request. Public datasets used or analyzed during this study are available in public domain.

## Acknowledgments

We thank all members of the Breakefield laboratory for their suggested ideas during laboratory meetings. We thank all laboratories within the Molecular Neurogenetics Unit at MGH for their input. We thank Dr. Casey Maguire for gifting the AAV-F in pAR-9 vector. We thank Dr. Dunn’s laboratory for generously providing the mImp3 peptide. We would like to thank Dr. Mark Issa for his expert advice on immunology within the TME. Special thanks to members of Dr. Mempel’s laboratory for sharing their expertise on the immune system and Dr. El Khoury’s laboratory for sharing their insight on tumor-associated microglia. We thank Mrs. Suzanne McDavitt for her skilled editorial assistance. X.O.B. acknowledges grant support from 10.13039/100000065National Institute of Neurological Disorders and Stroke NS122163, the 10.13039/100000002National Institutes of Health National Cancer Institute (NCI) CA179563, CA069246, and CA232103, and 10.13039/100000065NINDS NS122163 grants for supporting this work. U19 CA179563 was supported by the 10.13039/100015326NIH Common Fund, through the Office of Strategic Coordination/Office of the NIH Director. T.R.M. acknowledges grant support from 10.13039/100000002NIH grant R01 AL123349. D.R.-R. was supported by 10.13039/100002108Friedreich's Ataxia Research Alliance and fara Australia. K.B. is funded by R01 NS122163-01A1, 10.13039/100000002NIH K22 CA2802019-01, and 10.13039/100000005DOD HT9425-24-1-0119. T.R.L. acknowledges grant support from the 10.13039/501100005416Norwegian Research Council (grant no. 315566). L.N. acknowledges support from Prins Bernhard Cultuurfonds. S.M.v.d.L. acknowledges the Jo-Kolk scholarship.

## Author contributions

X.O.B. and K.B. conceived the study and designed the experiments. X.O.B. and K.B. supervised the project. T.S.v.S. initiated the project. K.B., T.R.L., L.N., S.M.v.d.L., T.X., and E.D.I. performed and analyzed the experiments. T.R.L. performed and imaged western blots. K.B. designed the plasmids and D.R.-R., S.M., and E.G. cloned the constructs. T.R.M. provided transgenic mice strains. T.R.M., J.K.L., and M.S. provided their expertise on the flow cytometry experiments, interpretation, and analysis. K.B., V.M., and A.J.E.M.d.R. performed bioinformatic analyses. K.B., C.P.C., C.A.E.F., and T.E.M. analyzed human scRNA-seq datasets. E.C.W., Y.S., and R.W.J. performed organotypic culture experiments. G.P.D. provided the mImp3 peptide and M.L. supported with the Elispot assay. L.N., T.R.L., and K.B. prepared the figures. T.R.L., L.N., and K.B. wrote the manuscript. All authors edited or commented on the manuscript.

## Declaration of interests

The authors declare no competing interests. PCT/US2023/075225 has been filed.

## Supplemental information

Document S1. Figures S1–S9 and Tables S1–S4

Table S5. List of primers used in this study

Table S6. Key resources table

Document S2. Article plus supplemental information
