# Supplementary material for: Intratumoral delivery of 4–1BBL boosts IL-12-triggered anti-glioma immunity
Source: Mol Ther. Author manuscript; Available in PMC 2025 Oct 7. (PMC12500232; doi:10.1016/j.ymthe.2025.08.028)
Supplement: Supplementary file 1 [file NIHMS2110668-supplement-MMC1.pdf]

YMTHE, Volume *33*

Supplemental Information

Intratumoral delivery of 4-1BBL boosts IL-12-triggered anti-glioma immunity

Taral R. Lunavat, Lisa Nieland, Sanne M. van de Looij, Alexandra J.E.M. de Reus, Charles P. Couturier, Chadi A. El Farran, Tyler E. Miller, Julia K. Lill, Maryam Schübel, Tianhe Xiao, Emilio Di Ianni, Elliot C. Woods, Yi Sun, David Rufino-Ramos, Thomas S. van Solinge, Shadi Mahjoum, Emily Grandell, Mao Li, Vamsi Mangena, Gavin P. Dunn, Russell W. Jenkins, Thorsten R. Mempel, Xandra O. Breakefield, and Koen Breyne

Table S1. Gene and protein nomenclature.

**Mouse**			**Human**		
Gene	RNA	Protein	Gene	RNA	Protein
Tnfsf9	*Tnfsf9*	41BBL	*TNFSF9*	*TNFSF9*	41BBL
Tnfrsf9	*Tnfrsf9*	41BBL	*TNFRSF9*	*TNFRSF9*	41BBL
Pdcd1	*Pdcd1*	PD-1	*PDCD1*	*PDCD1*	PD-1
Cd274	*Cd274*	PD-L1	*CD274*	*CD274*	PD-L1
Havcr2	*Havcr2*	TIM-3	*HAVCR2*	*HAVCR2*	TIM-3
Tcf7	*Tcf7*	TCF-1	*TCF7*	*TCF7*	TCF-1
I12a	*Il12a*	IL12A	*IL12A*	*IL12A*	IL12A
Il12b	*Il12b*	IL12B	*IL12B*	*IL12B*	IL12B

Table S2. scRNAseq datasets.

Dataset	Sample type	Enriched for specific cell types	Reference
**Human glioma**			
Mathewson 2021	IDH mutant and wild-type	Yes - T cells	[1]
Miller 2023	IDH mutant and wild-type	No - all cells	[2]
Pombo Antunes 2021	IDH wild-type	Yes - $CD45^{POS}$ cells	[3]
**Murine glioblastoma**			
Tomaszewski 2022	CT-2A	Yes - $CD45^{POS}$ cells	[4]
Chen 2023	005	Yes - $CD45^{POS}$ cells	[5]
Pombo Antunes 2021	GL261	Yes - $CD45^{POS}$ cells	[3]

Table S3. Murine GB cell line characterization and genetics.

GB model	Established	Pro-lifera-tive	Inva-sive	Histology	Genetic	Ref-er-ence
CT-2A	Chemical induction of methylcholanthrene Subcutaneous	+++	-	Astrocy-toma	*p53* WT/*Pten* deficient	[6,7]
GL261	Chemical induction of methylcholanthrene Intracranially	++	-	Ependy-moblas-toma	*K-Ras* mu-tant/*p53* mu-tant; *Pten* de-ficient	[6,7]
005	Retroviral transduc-tion	+	+++	High-grade glioma	*H-ras/AKT* activation; *Pten* ele-vated; *p53*+/-	[7,8]

Table S4. Re-implanted mice.

Strain	First implant	Survival time	Second implant	Outcome
BL6 WT mice	8/2/23		4/12/23	
1	CT-2A-FLuc-Tnfsf9	10 months	CT-2A-FLuc-Tnfsf9	tumor at day 16
2	CT-2A-FLuc-Tnfsf9	10 months	CT-2A-FLuc-Tnfsf9	tumor at day 16
3	CT-2A-FLuc-Tnfsf9	10 months	CT-2A-FLuc-Tnfsf9	No tumor
**Strain**	**First implant**	**Survival time**	**Second implant**	**Outcome**
BL6 WT mice	11/2/22		6/7/22	
4	CT-2A-FLuc-Tnfsf9	5 months	CT-2A-FLuc	no tumor
5	CT-2A-FLuc-Tnfsf9	5 months	CT-2A-FLuc	no tumor
6	CT-2A-FLuc-Tnfsf9	5 months	CT-2A-FLuc	no tumor
7	CT-2A-FLuc-Tnfsf9	5 months	CT-2A-FLuc	tumor at day 14
**Strain**	**First implant**	**Survival time**	**Second implant**	**Outcome**
BL6 WT mice	8/2/22		15/01/24	
8	CT-2A-FLuc-Tnfsf9	23 months	CT-2A-FLuc	tumor at day 10
9	CT-2A-FLuc-Tnfsf9	23 months	CT-2A-FLuc	tumor at day 30
10	CT-2A-FLuc-Tnfsf9	23 months	CT-2A-FLuc	no tumor
11	CT-2A-FLuc-Tnfsf9	23 months	CT-2A-FLuc	no tumor
12	CT-2A-FLuc-Tnfsf9	23 months	CT-2A-FLuc	no tumor
13	CT-2A-FLuc-Tnfsf9	23 months	CT-2A-FLuc	no tumor
**Strain**	**First implant**	**Survival time**	**Second implant**	**Outcome**
BL6 WT mice	12/1/24		14/03/2024	
1	005-FLuc-Tnfsf9	3 months	005-FLuc	No tumor
2	005-FLuc-Tnfsf9	3 months	005-FLuc	No tumor
3	005-FLuc-Tnfsf9	3 months	005-FLuc	No tumor
4	005-FLuc-Tnfsf9	3 months	005-FLuc	No tumor

Table S5: List of primers used in this study.

(See Supplemental Videos and Spreadsheets)

Table S6: Key resources table.

(See Supplemental Videos and Spreadsheets)

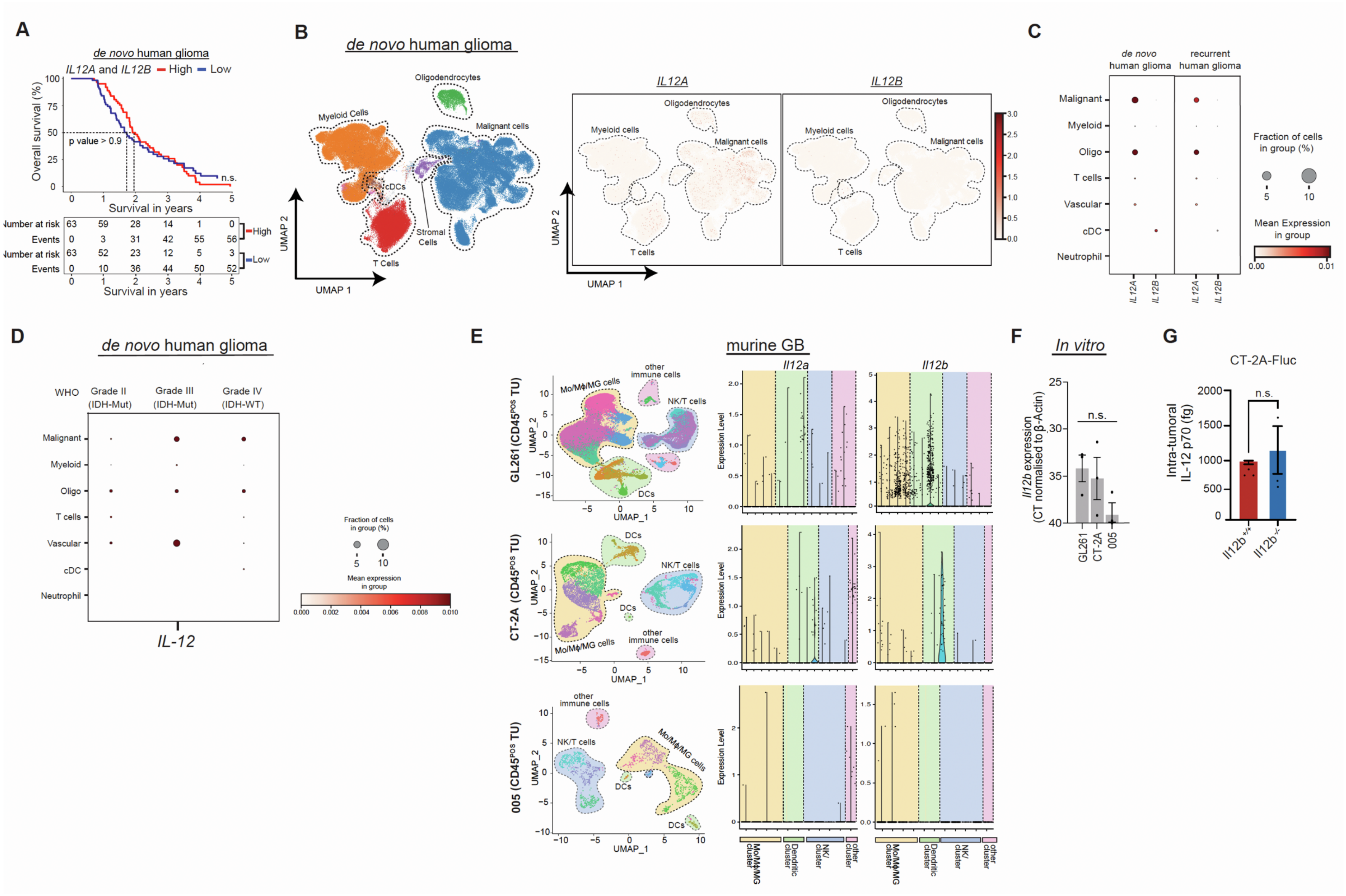

A
de novo human glioma
IL12A and IL12B High Low
Overall survival (%)
p value > 0.9
n.s.
Survival in years
Number at risk 63 59 28 14 1 0 High
Events 0 3 31 42 55 56
Number at risk 63 52 23 12 5 3 Low
Events 0 10 36 44 50 52
B
de novo human glioma
Oligodendrocytes
Myeloid Cells
Malignant cells
cDCs
Stromal Cells
T Cells
UMAP 1
UMAP 2
IL12A
IL12B
C
de novo human glioma
recurrent human glioma
Malignant
Myeloid
Oligo
T cells
Vascular
cDC
Neutrophil
Fraction of cells in group (%)
Mean Expression in group
D
de novo human glioma
WHO
Grade II (IDH-Mut)
Grade III (IDH-Mut)
Grade IV (IDH-WT)
IL-12
E
murine GB
GL261 (CD45POS TU)
CT-2A (CD45POS TU)
005 (CD45POS TU)
Mo/Mϕ/MG cells
other immune cells
NK/T cells
DCs
UMAP_1
UMAP_2
Il12a
Il12b
Expression Level
Mo/Mϕ/MG cluster
Dendritic cluster
NK/ cluster
other cluster
F
In vitro
Il12b expression (CT normalised to β-Actin)
n.s.
GL261
CT-2A
005
G
CT-2A-Fluc
Intra-tumoral IL-12 p70 (fg)
n.s.
Il12b+/+
Il12b-/-

Figure S1. IL-12A and IL-12B expression across human and murine glioma datasets.

(A) *Survival probability of de novo human glioma differentiating high and low expressing IL12A and IL12B.* Kaplan-Meier survival curves showing the overall survival outcomes over a period of 5 years of a total of 63 glioma patients (IDH-WT) per group with high (red) or low (blue) levels of *IL12A* and *IL12B* combined, each group had a median survival of ~2 years based on Miller *et al.*[2]. Samples in the top 33% in terms of expression of genes of interest (or module scores) were labeled as "high." The bottom 33% were considered the "low" group. No differences were observed between groups. Log-rank (Mantel-Cox) test, p-value = 0.9, not significant (n.s.).

(B) *UMAP projections of expression of IL12A and IL12B in de novo human glioma.* The scRNAseq dataset of human glioma cells from Miller *et al.*,[2] were analyzed. Distinct cell type subsets were clustered, annotated and visualized with a high-resolution color coded UMAP projection. Malignant cells, Myeloid cells, oligodendrocytes, DCs, T cells and stromal cells are depicted (left). *IL12A* and *IL12B* UMAP projections show low expression in each cell cluster (right).

(C) *IL12A and IL12B expression in de novo human glioma and recurrent glioma.* Dot plot showed that the expression of IL12A and IL12B were present at low levels in both primary and recurrent glioma (Datasets from Miller *et al.*,)[2].

(D) *Expression of IL12A and IL12B at different grade levels of de novo human glioma.* Human *IL12A* and *IL12B* were expressed at low levels in IDH mutant human glioma grade II, III and IV (including WT glioma). No significant difference was observed between the glioma grades. (Datasets from Miller *et al.*,)[2].

(E) *UMAP projections of immune cell populations in murine GB.* scRNAseq datasets of murine GB were analyzed (Datasets from Pombo Antunes *et al.,* Tomaszewski *et al.,* and Chen *et al.*)[3-5]. Distinct cell types were clustered, annotated and visualized with a high-resolution color coded UMAP projection. To visualize *Il12a* and *Il12b* expression in different cell lines, single cell violin plots were used to compare transcript levels in Mo/Mφ/MG cells cluster (TAM, proliferative TAM and monocytes), dendritic cells cluster (DC1, DC2, DC3 and DC4), NK/T cells cluster (reg T cells, NK cells, T cells) and other cell

cluster (B cells, plasma B cells, mast cells). The data suggested that *Il12b* was expressed at minimal levels in DC cells.

(F) *Murine GB cell lines express minimal levels of Il12b.* GL261, CT-2A and 005 cells show low expression levels of *Il12b.* Data represent three independent experiments and are presented as the mean with SEM (error bars). Data were analyzed using one way ANOVA, not significant (n.s.).

(G) *IL-12 protein levels in GB-containing brain hemisphere.* IL-12 levels femtogram (fg) were determined using Luminex in GB-bearing (CT-2A-FLuc) *Il12*$^{+/+}$ (red) and *Il12*$^{-/-}$ (blue) mice. Values were normalized to the non-tumor hemisphere. Data represents three independent experiments and are presented as the mean with SEM (error bars). Unpaired t-test, not significant (n.s).

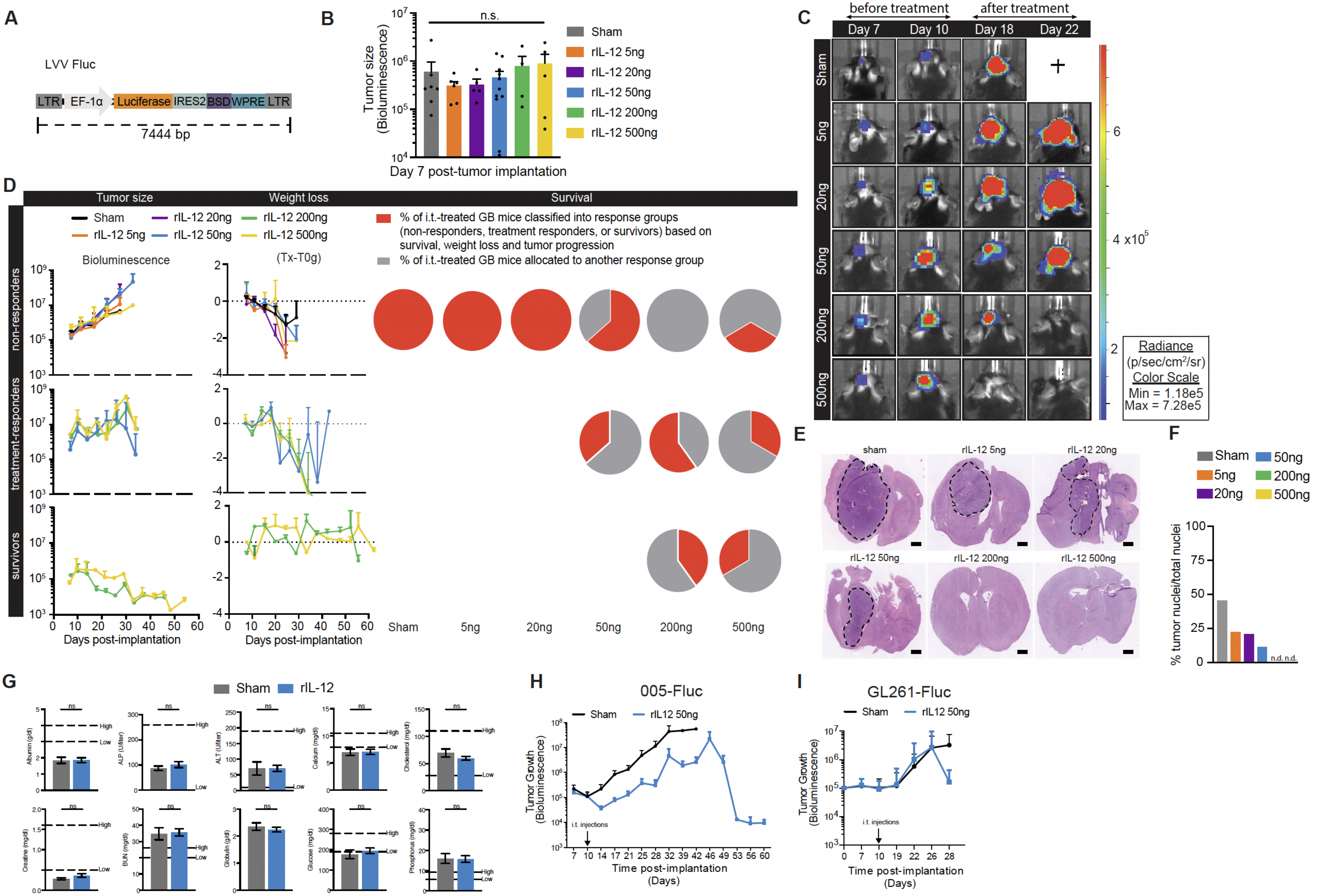
A
LVV Fluc
LTR EF-1α Luciferase IRES2 BSD WPRE LTR
7444 bp
B
n.s.
Tumor size (Bioluminescence)
Day 7 post-tumor implantation
Sham
rIL-12 5ng
rIL-12 20ng
rIL-12 50ng
rIL-12 200ng
rIL-12 500ng
C
before treatment
after treatment
Day 7
Day 10
Day 18
Day 22
Radiance (p/sec/cm²/sr)
Color Scale
Min = 1.18e5
Max = 7.28e5
D
Tumor size
Weight loss
Survival
non-responders
treatment-responders
survivors
Bioluminescence
(Tx-T0g)
% of i.t.-treated GB mice classified into response groups (non-responders, treatment responders, or survivors) based on survival, weight loss and tumor progression
% of i.t.-treated GB mice allocated to another response group
Days post-implantation
E
sham
rIL-12 5ng
rIL-12 20ng
rIL-12 50ng
rIL-12 200ng
rIL-12 500ng
F
% tumor nuclei/total nuclei
n.d. n.d.
G
Sham
rIL-12
H
005-Fluc
Tumor Growth (Bioluminescence)
i.t. injections
Time post-implantation (Days)
I
GL261-Fluc
rIL12 50ng

Figure S2. Intra-tumoral administration of recombinant IL-12 induces therapeutic responses in murine glioma.

(A) *Lentiviral construct.* Schematic display of LVV-Firefly Luciferase (FLuc) containing luciferase, IRES2, BSD and WPRE driven by the EF-1α promoter.

(B) *Bioluminescence measurements.* To ensure equal tumor size between groups, CT-2A-FLuc bioluminescence levels were measured in GB-bearing mice at day 7 post-tumor implantation, prior to i.t. treatment of sham or rIL-12. No significant differences were observed between groups (50 ng sham, n= 7; 5 ng rIL-12, n = 6; 20 ng rIL-12, n= 5; 50 ng rIL-12, n= 11; 200 ng rIL-12, n=5, and 500 ng rIL-12, n=6). Multiple t-test, not significant (n.s.)

(C) *Regression of tumors in GB-bearing mice with rIL-12 over time.* FLuc readings demonstrate that tumors were established in mice brains before treatment on days 7 and 10. Different outcomes were observed after treatment on days 18 and 22 post tumor-implantation depending on the rIL-12 dose that GB-bearing mice received. The shown images are representative in vivo imaging system (IVIS) images of GB-bearing mice from each treatment condition and "+" indicates that animals died prior to the imaging timepoint.

(D) *Classification of rIL-12 treated GB-bearing mice based on multiple parameters.* Based on tumor growth and weight loss, three categories could be distinguished. Non-responders (n=27) represent mice that had similar results as the sham treatment. Treatment-responders (n=9) represent mice that performed better than the sham-treated mice, but still died. Survivors (n=4) represent mice that had visible tumor regression due to treatment. In the left graphs, CT-2A-FLuc-bearing mice treated rIL-12 or sham were monitored every 3-4 days by IVIS, which is representative of the tumor size in the brain. Dotted line represents the background signal. In the middle graphs, the weight of the mice was tracked over time. The dotted line represents the weight at start of the experiment (T_0), while Tx denotes specific time points at which weights were measured. Weight loss was normalized to each mouse its initial body weight and expressed in grams relative to the starting weight at treatment onset (Tx–T0). The pie graphs on right represent the percentage of mice that are allocated to a certain category based on

survival, weight, and tumor size. Data represents two independent experiments and are presented as the mean with SEM (error bars).

(E) *Tumor sizes in brains of rIL-12 treated GB-bearing mice*. Brain sections of mice 22 days post-i.c. implantation with CT-2A-FLuc and i.t. treatment with sham or rIL-12 (n=1 per dosage) were stained for hematoxylin and eosin (H&E) (4x magnification, scale bar = 5 µm). The black dotted line indicates the tumor border.

(F) *Hematoxylin & Eosin.* Quantification of H&E histology images shown as the percentage of tumor nuclei over the total number of nuclei. Images were analyzed by Image J using the color deconvolution plugin (n=1 per group; n.d. – not determined).

(G) *Blood chemistry analysis.* Whole blood from GB-bearing mice treated with 50 ng rIL-12 (n=3) or sham (n=3) showed no significant differences for systemic toxicity at post-tumor implantation. Albumin, Alkaline Phosphatase (ALP), Alanine transaminase (ALT), calcium, cholesterol, creatine, blood urea nitrogen (BUN), globulin, glucose and phosphorus were determined. (n=4-5 mice per group). Data represents two independent experiments and are presented as the mean with SEM (error bars). Data were analyzed using unpaired t-test, not significant (n.s.).

(H&I) Average bioluminescence intensity (BLI) levels of 005-FLuc and GL261-FLuc tumor-bearing mice were measured over time comparing sham (solid black) and rIL-12 (solid green) (n=4-5 mice per group).

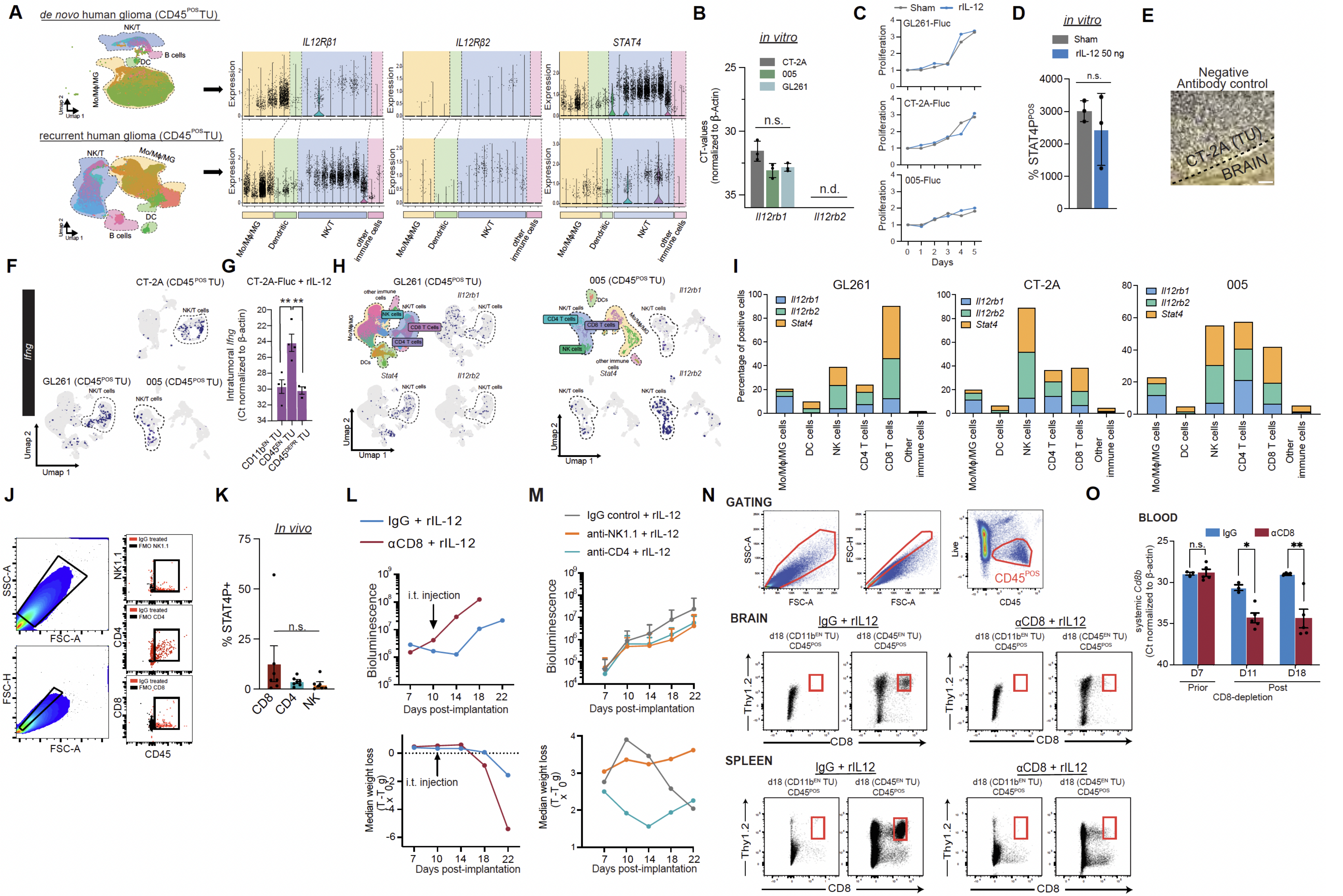
A
de novo human glioma (CD45POS TU)
recurrent human glioma (CD45POS TU)
NK/T
B cells
DC
Mo/Mϕ/MG
IL12Rβ1
IL12Rβ2
STAT4
Expression
Mo/Mϕ/MG
Dendritic
NK/T
other immune cells
B
in vitro
CT-2A
005
GL261
n.s.
n.d.
CT-values (normalized to β-Actin)
Il12rb1
Il12rb2
C
Sham
rIL-12
GL261-Fluc
CT-2A-Fluc
005-Fluc
Proliferation
Days
D
in vitro
Sham
rIL-12 50 ng
n.s.
% STAT4PPOS
E
Negative Antibody control
CT-2A (TU)
BRAIN
F
Ifng
CT-2A (CD45POS TU)
GL261 (CD45POS TU)
005 (CD45POS TU)
NK/T cells
Umap 1
Umap 2
G
CT-2A-Fluc + rIL-12
Intratumoral Ifng (Ct normalized to β-actin)
CD11bEN TU
CD45EN TU
CD45DEPR TU
H
GL261 (CD45POS TU)
005 (CD45POS TU)
Il12rb1
Il12rb2
Stat4
other immune cells
NK cells
CD8 T cells
CD4 T cells
DCs
I
GL261
CT-2A
005
Percentage of positive cells
Il12rb1
Il12rb2
Stat4
Mo/Mϕ/MG cells
DC cells
NK cells
CD4 T cells
CD8 T cells
Other immune cells
J
SSC-A
FSC-A
FSC-H
NK1.1
CD4
CD8
CD45
IgG treated
FMO NK1.1
FMO CD4
FMO CD8
K
In vivo
% STAT4P+
n.s.
CD8
CD4
NK
L
IgG + rIL-12
αCD8 + rIL-12
Bioluminescence
i.t. injection
Days post-implantation
Median weight loss (T-T0 g)
M
IgG control + rIL-12
anti-NK1.1 + rIL-12
anti-CD4 + rIL-12
Bioluminescence
Days post-implantation
Median weight loss (T-T0 g)
N
GATING
SSC-A
FSC-H
Live
FSC-A
CD45
CD45POS
BRAIN
IgG + rIL12
αCD8 + rIL12
d18 (CD11bEN TU) CD45POS
d18 (CD45EN TU) CD45POS
Thy1.2
CD8
SPLEEN
O
BLOOD
IgG
αCD8
n.s.
*
**
systemic Cd8b (Ct normalized to β-actin)
D7
D11
D18
Prior
Post
CD8-depletion

Figure S3. Expression and functional analysis of IL-12 receptor components and downstream STAT4 signaling in glioma-infiltrating immune subsets.

(A) *Expression of IL12Rβ1, IL12Rβ2 and STAT4 in de novo and recurrent human glioma in immune cell populations*. scRNAseq cluster datasets of CD45^{POS}-sorted cells derived from *de novo* human glioma tumors (n=7) and human recurrent glioma tumors (n=4) were analyzed (left). Distinct cell type subsets were clustered, annotated and visualized with a high-resolution color coded UMAP projection. To visualize *IL12Rβ1, IL12Rβ2* and *STAT4* expression in different datasets, single cell violin plots were used to compare transcript levels in Mo/Mϕ/MG cells (TAM, proliferative, TAM and monocytes), dendritic cells (DC1, DC2, DC3 and DC4), NK/T cells (reg T cells, NK cells, T cells) and other cell cluster (B cells, plasma B cells, mast cells) (Datasets from Pombo Antunes *et al.,* Tomaszewski *et al.,* and Chen *et al.*)[3-5].

(B) *Expression of Il12rb1/2 in mouse cell lines.* The expression of *Il12rb1* was determined in three different mouse glioma cell lines (CT-2A, 005 and GL261) using qRT-PCR. The expression of *Il12rb1* did not show any significant difference, whereas *Il12rb2* was not determined (n.d.) in any of the cell lines. Data represent three independent experiments and were analyzed using two-way ANOVA multiple comparison test, not significant (n.s.).

(C) *Cell viability assay.* CT-2A-FLuc, 005-FLuc and GL261-FLuc cell lines were exposed to 50 ng rIL-12 or sham control and cell viability (proliferation) was measured over a period of 5 days. Data represents triplicates. No significant differences were observed between groups, unpaired t-test.

(D) *Quantification of STAT4P in CT-2A-FLuc cultured cells.* CT-2A-FLuc cells were exposed to sham or rIL-12 for 24 hours, STAT4P protein levels were quantified by flow cytometry. Data represent three independent experiments and are presented as the mean with SEM (error bars). Data was analyzed using paired t-test, not significant (n.s.).

(E) *Primary antibody (IL12RB1) control staining in brain tissues implanted with a CT-2A tumor.* Immunohistochemistry negative control lacking primary antibody (IL12RB1) in the CT-2A tumor (TU) cells in the brain TME (magnification 40x; scale bar = 100 µm).

(F) *Expression of Ifn-γ in immune cells populations of mouse GB cell lines*. scRNAseq cluster datasets of CD45^{POS}-sorted tumor cells derived from GL261 [3], CT-2A [4] and 005 [5] cells were analyzed. Distinct cell type subsets were clustered, annotated and visualized with a coded UMAP projection and NK/T cells were highlighted with a dotted line. (Datasets from Pombo Antunes *et al.,* Tomaszewski *et al.,* and Chen *et al.*)

(G) *Expression of Ifn-γ in CT-2A-FLuc implanted mice.* The brain tissue was analyzed eight days after rIL12-treatment. *Ifn-γ* was expressed significantly higher in CD45^{EN} TU compared to CD11b^{EN} TU (p-value = 0,0075) and CD45^{DEPR} TU cells (p-value = 0.0067). Data represent CT values normalized to β-actin. Data represent three independent experiments and are presented as the mean with SEM (error bars), Data were analyzed using one-way ANOVA, **p < 0.01.

(H) *Expression of Il12rb1, Il12rb2 and Stat4 in GL261 and 005 murine GB cell lines*. scRNAseq cluster datasets of CD45^{POS}-sorted cells derived from GL261 (n=3) [3], and 005 (n=4) [5] tumor-bearing C57BL6 mice were analyzed (left). Distinct cell type subsets were clustered, annotated and visualized with a high-resolution color coded UMAP projection. To visualize *Il12rb1*, *Il12rb2* and *Stat4* expression in different datasets, single cell feature plots were used to compare transcript levels in NK/T cells cluster (reg T cells, NK cells, T cells) as demonstrated by dotted lines. (Datasets from Pombo Antunes *et al.,* and Chen *et al.*)

(I) *Percentages of positive cells in each immune cell cluster represented by bar graphs.* Datasets (**Table 1**) were analyzed for *Il12rb1, Il12rb2* and *Stat4* expression in GL261, CT-2A and 005 GB cells, including Mo/Mϕ/MG cells, dendritic cells, natural killer cells, Regulatory T cells, T cells, and other immune cells clusters. (Datasets from Pombo Antunes *et al.,* Tomaszewski *et al.,* and Chen *et al.*)[3-5].

(J) *Gating strategy for identifying lymphocyte subsets by flow cytometry.*

Forward scatter area (FSC-A) vs. forward scatter height (FSC-H) was used to gate singlets, followed by gating on populations based on side scatter (SSC-A) vs. FSC-A. Subsequent gating was performed to identify CD4^{POS}, CD8^{POS}, and NK1.1^{POS} cell populations. Cells gated for CD45 were further analyzed for CD4, CD8 and NK1.1 expression. Red dots represent cells from the IgG-treated fraction. Black dots represent FMO for CD8 ^{POS}, CD4 ^{POS}, and NK1.1 ^{POS}. Black box represent positive signal for each of the markers.

(K) *Quantification of STAT4p percentages in CD8, CD4 T cells and NK1.1 cells.* Bar graphs represent quantification of the percentages of STAT4p^{POS} cells within CD8/CD4/NK cells (n=6 mouse/group). CD8 T cells showed the highest percentage of STAT4p (12.6%), followed by CD4 T cells (3.7%) and NK cells (2.3%). Data represent three independent experiments and are presented as the mean with SEM (error bars). Data was analyzed using multiple comparison one-way ANOVA, not significant (n.s.).

(L) *Tumor growth and weight* were measured over time in tumor-bearing mice injected with IgG and rIL-12 (solid blue), anti-CD8 and rIL-12 (solid red). After T-cell depletion, mice had increased tumor sizes as measured by BLI. Weights of all mice dropped starting day 14 after tumor cell injection. (n = 6-8 mice per group). Weight loss was normalized to each mouse its initial body weight and expressed in grams relative to the starting weight at treatment onset (Tx–T0).

(M) *Tumor growth and mice weight* were measured over time in tumor-bearing mice injected with anti-CD4 or anti-NK1.1 compared to IgG control all treated i.t. with rIL-12 at day 10 post-tumor implantation. BLI signal did not differ between groups. Weights of NK1.1 depleted mice increased over time whereas CD4-depleted mice had reduced weights over time. (n = 6-8 mice per group).

(N) *Validation of CD8^{POS} T cell depletion* shown by the absence of CD8^{POS} cells in representative flow cytometry plots showing cell fractions for CD11b^{EN} TU or CD45^{EN} TU, pre-gated for CD45^{EN} cells, after treatment with anti-CD8 compared to IgG control for brain and spleen samples.

(O) *Systemic Cd8b expression levels measured over time.* Gene expression levels in retro-orbital blood samples obtained from CD8-depleted mice (n=5) show a significant reduction in *Cd8b* at day 11 and 18 (*p = 0.014, **p = 0.0013, respectively), post CD8-depletion compared to IgG control mice (n=3); no

significant drop of *Cd8b* was observed at day 7 (prior to CD8-depletion). Data represent two independent experiments and are presented as the mean with SEM (error bars). Data was analyzed using two-way ANOVA, *p<0.05, **p<0.01, not significant (n.s.).

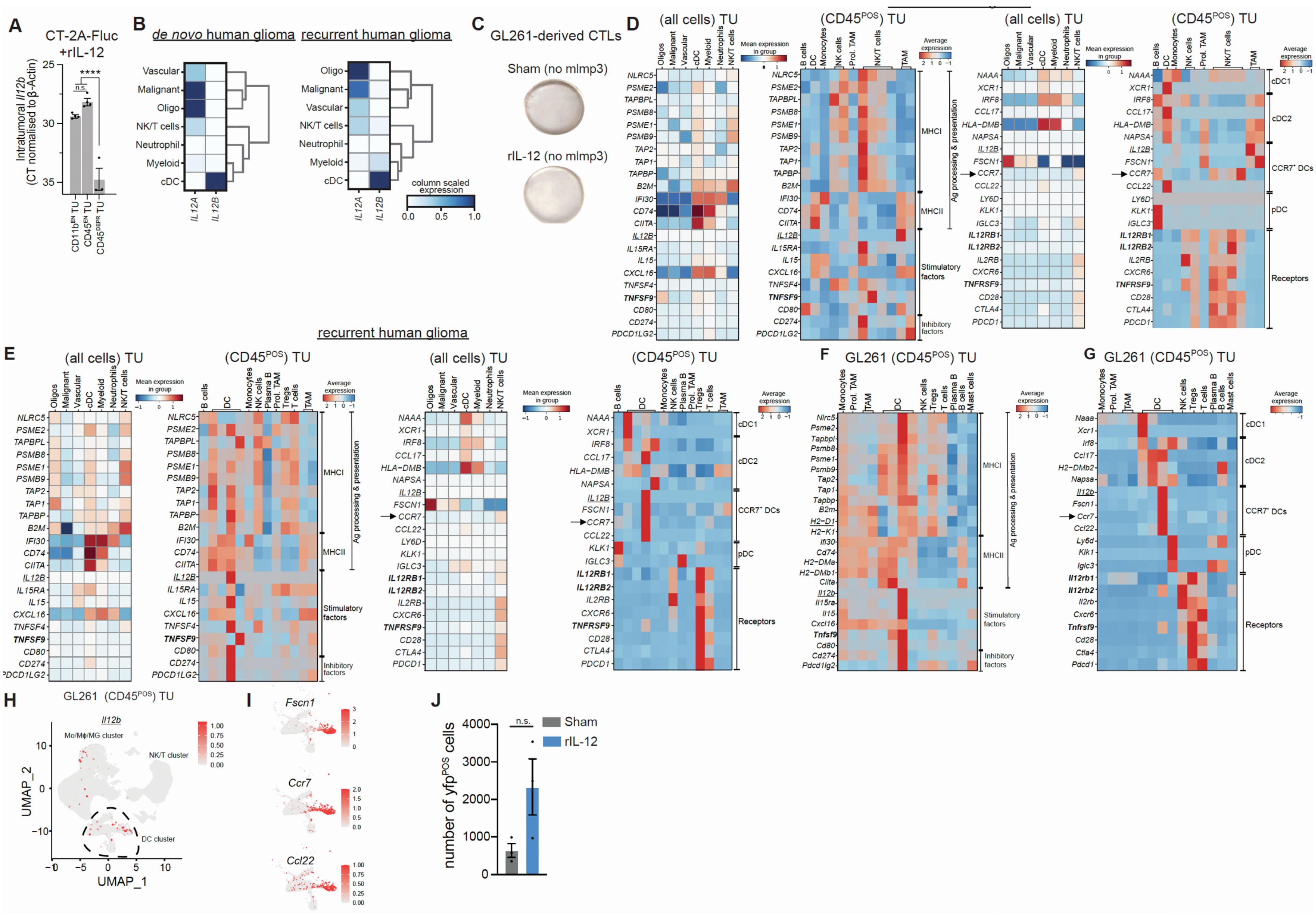
A
CT-2A-Fluc +rIL-12
Intratumoral Il12b (CT normalised to β-Actin)
n.s.
****
CD11b^EN TU
CD45^EN TU
CD45^DEPR TU
B
de novo human glioma
recurrent human glioma
Vascular
Malignant
Oligo
NK/T cells
Neutrophil
Myeloid
cDC
IL12A
IL12B
column scaled expression
C
GL261-derived CTLs
Sham (no mImp3)
rIL-12 (no mImp3)
D
(all cells) TU
(CD45^POS) TU
Mean expression in group
Average expression
MHCI
MHCII
Ag processing & presentation
Stimulatory factors
Inhibitory factors
cDC1
cDC2
CCR7+ DCs
pDC
Receptors
recurrent human glioma
E
(all cells) TU
(CD45^POS) TU
F
GL261 (CD45^POS) TU
G
GL261 (CD45^POS) TU
H
GL261 (CD45^POS) TU
Il12b
Mo/Mφ/MG cluster
NK/T cluster
DC cluster
UMAP_1
UMAP_2
I
Fscn1
Ccr7
Ccl22
J
number of yfp^POS cells
n.s.
Sham
rIL-12

Figure S4. Dendritic cells activation and antigen presentation signatures in human and murine glioma.

(A) *Intratumoral Il12b* expression in CT-2A-FLuc cell populations post-rIL12 treatment. *Il12b* was predominantly expressed by CD11b^{EN} TU and CD45^{EN} TU cells compared to CD45^{DEPR} TU cells. Data represent three independent experiments and are presented as the mean with SEM (error bars). Data were analyzed using one way ANOVA, ****$p < 0.0001$, not significant (n.s.).

(B) *IL12A and IL12B expression in de novo human glioma and recurrent glioma.* Using hierarchical clustering, *IL12B* expression was found to be highest in the dendritic cell (DC) cluster across both primary and recurrent glioma samples (dataset from Miller *et al.*)[2] .

(C) *Representative images of* IFN-γ *Elispot assay.* Control groups showing minimal number of IFN-γ positive spots when CD8^{POS} T cells and naïve splenocytes were co-cultured without mImp3 (GL261-specific peptide) either with Fc (top) or rIL-12 (bottom) treatment for 24h.

(D) *Heatmaps of de novo human glioma showing regulatory factors for CTLs. IL12B* was not detected in de novo patient glioma datasets due to lack of sufficient tumor associated DCs. The arrow points to the *CCR7* gene (Datasets from Mathewson *et al.,* Miller *et al.,* and Pombo Antunes *et al.,*)[1-3].

(E) *Heatmaps of recurrent human glioma showing regulatory factors for CTLs.* In the dataset where immune cells were enriched, *IL12B*-expressing DCs could be detected and displayed *CCR7* and *TNFRSF9* (encoding for 4-1BB) co-expression. *IL12R, TNFSF9, IL12RB1, IL12RB2* and *TNFRSF9* are highlighted in bold. The arrow points to the *CCR7* gene. Datasets from Mathewson *et al.,* Miller *et al.,* and Pombo Antunes *et al.,*)[1-3].

(F & G) GL261 data confirms CT-2A associated DCs with CTL modulating capabilities. Heatmaps showing co-expression of CCR7^{POS} DC cluster genes in GL261 CD45^{POS} tumor cell dataset [3]. *H2-d1, Il12rb1, Il12rb2*, *Il12b and Tnfrsf9* are highlighted in bold. The arrow points at the *Ccr7* gene. (Dataset from Pombo Antunes *et al.*)[3].

(H & I) *Il12b-DCs have migratory potential*. *Il12b* was highly expressed in Ccr-7^{POS}-DCs subcluster in a GL261 (CD45^{POS}) tumor (TU); (dataset from Tomaszewski *et al.,*)[3]. *Ccr-7^{POS}-DCs in scRNAseq*

dataset. The cells positive in panel H (marked with dotted line) match with the dendritic specific markers *Fscn1, Ccr7,* and *Ccl22* in GL261 (CD45^{POS}) tumor (TU). (Dataset from Pombo Antunes *et al.*)[3].

(J) *Quantification of IHC images showing the number of yfp^{POS} cells.* The number of yfp^{POS} to label IL-12^{POS} DCs were quantified at the tumor border (CT-2A-FLuc) using Image J comparing sham and rIL-12 treated mice (n=3 per group). Although a higher number of yfp^{POS} cells were observed post rIL-12 therapy, no significant differences between groups were observed. Data are presented as the mean with SEM (error bars). Data were analyzed using unpaired t-test, not significant (n.s.)

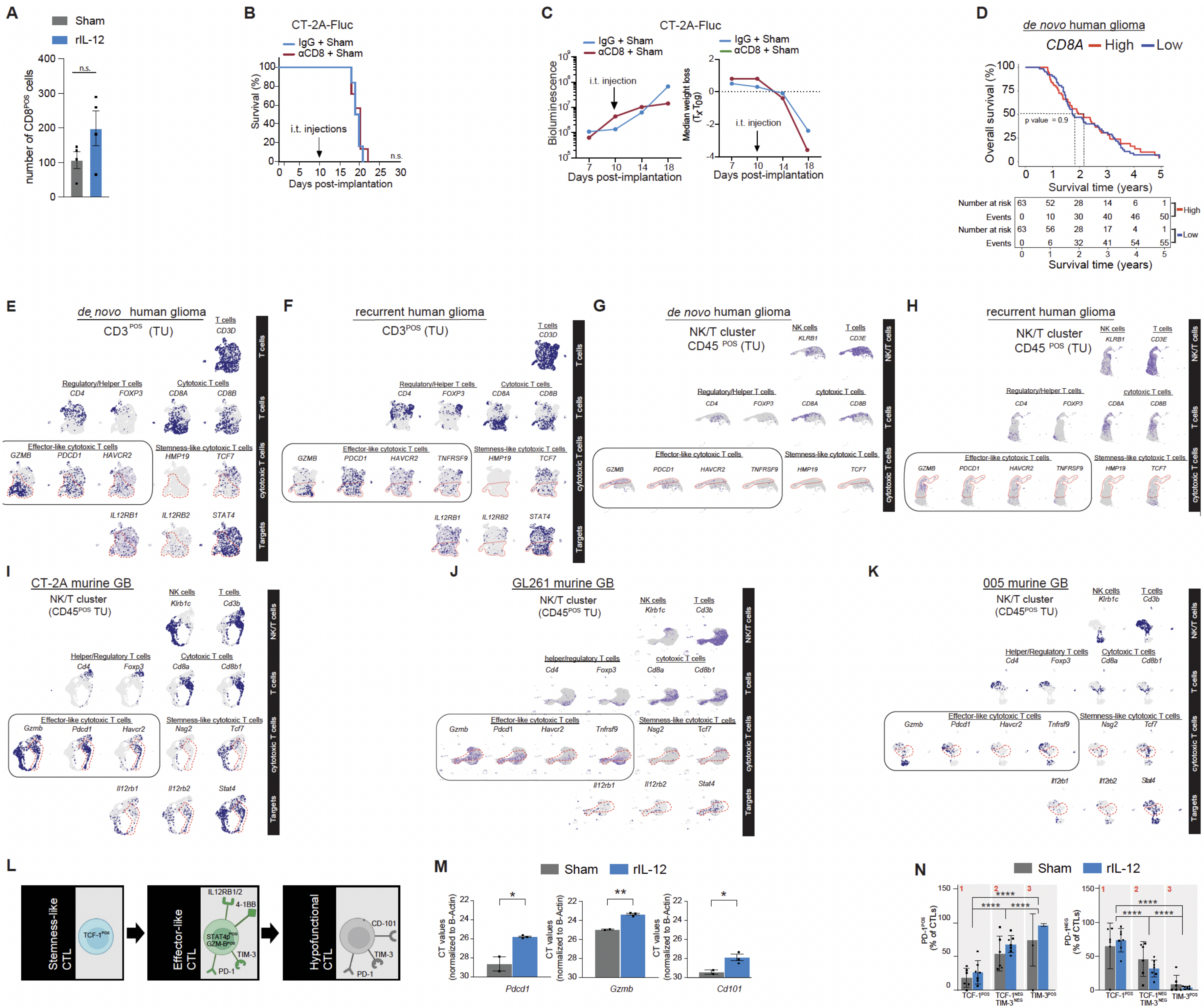

A
Sham
rIL-12
number of CD8POS cells
n.s.
B
CT-2A-Fluc
IgG + Sham
αCD8 + Sham
Survival (%)
i.t. injections
n.s.
Days post-implantation
C
CT-2A-Fluc
IgG + Sham
αCD8 + Sham
Bioluminescence
i.t. injection
Days post-implantation
Median weight loss
i.t. injection
Days post-implantation
D
de novo human glioma
CD8A High Low
Overall survival (%)
p value = 0.9
Survival time (years)
Number at risk 63 52 28 14 6 1
Events 0 10 30 40 46 50
High
Number at risk 63 56 28 17 4 1
Events 0 6 32 41 54 55
Low
Survival time (years)
E
de novo human glioma
CD3POS (TU)
Regulatory/Helper T cells
Cytotoxic T cells
Effector-like cytotoxic T cells
Stemness-like cytotoxic T cells
F
recurrent human glioma
CD3POS (TU)
G
de novo human glioma
NK/T cluster
CD45 POS (TU)
H
recurrent human glioma
NK/T cluster
CD45 POS (TU)
I
CT-2A murine GB
NK/T cluster
(CD45POS TU)
J
GL261 murine GB
NK/T cluster
(CD45POS TU)
K
005 murine GB
NK/T cluster
(CD45POS TU)
L
Stemness-like CTL
TCF-1POS
Effector-like CTL
IL12RB1/2
4-1BB
STAT4pPOS
GZM-BPOS
TIM-3
PD-1
Hypofunctional CTL
CD-101
TIM-3
PD-1
M
Sham
rIL-12
CT values (normalized to B-Actin)
Pdcd1
Gzmb
Cd101
N
Sham
rIL-12
PD-1POS (% of CTLs)
PD-1NEG (% of CTLs)
TCF-1POS
TCF-1NEG TIM-3NEG
TIM-3POS

Figure S5. rIL-12 modulates CD8 T cell landscape in glioma by promoting effector differentiation.

(A) *Quantification of the number of CD8^{POS} cells as determined by at mouse tumor.* The number of CD8^{POS} T cells was quantified at the tumor border (CT-2A-FLuc) using Image J, comparing sham and rIL-12-treated mice. Although a higher number of CD8^{POS} cells was observed post rIL-12 therapy, no significant differences between groups were observed. Data are presented as the mean with SEM (error bars). Data were analyzed using unpaired t-test, not significant (n.s.).

(B) *Lack of CD8 T cell-mediated immunity in the CT-2A model without anti-GB therapy.* Kaplan-Meier curves showing survival outcome of tumor-bearing mice injected without CD8-depletion (IgG) (solid blue) and with CD8 depletion (anti-CD8b) (solid green), (n = 6-8 mice/group). Both groups had a median survival of 20 days. Log-rank (Mantel-Cox) test, not significant (n.s.).

(C) Tumor growth and body weight were measured over time in tumor-bearing mice injected with IgG and sham, anti-CD8 and sham (panel B). After T-cell depletion, mice had increased tumor sizes as measured by BLI. The weights of all mice dropped starting on day 14 after tumor cell injection. Weight loss was normalized to each mouse its initial body weight and expressed in grams relative to the starting weight at treatment onset (Tx–T0).

(D) *Survival probability of de novo human glioma, differentiating high and low CD8a.* Kaplan-Meier survival curves based on Miller *et al.*,[2] showing the survival outcomes over a period of 5 years of 63 glioma patients (IDH-WT) per group with high (red) or low (blue) levels of *CD8a*, each group had a median of ~2 years[2]. No differences were observed between groups. Log-rank (Mantel-Cox) test, p-value = 0.9, not significant (n.s). Median survival in days (MS).

(E, F, G, H) *Differentiating stemness- and effector-like CTLs in de novo and recurrent glioma*. scRNAseq analysis distinguishes, if present, NK cells from T cells based on *KLRB1* and *CD3E* expression, respectively. Then, helper and regulatory T cells, expressing *CD4* and *FOXP3* genes, were discriminated from the CD8^{POS} T cells marked by *CD8A/B* expression. In the CD8^{POS} T cells cluster, we observed naïve and stemness-like CD8^{POS} T cells, expressing *TCF7* (encoding TCF-1) and *HMP19*

genes, that were different from the CD8^{POS} T cells with an effector-like phenotype expressing *HAVCR2* (encoding TIM-3), *PDCD1* (encoding PD-1), *GZM-B* (encoding cytotoxic granzyme-B) and *TNFRSF9* (encoding 4-1BB). The targets *IL12RB1, IL12RB2*, and *STAT4* were also identified in the effector-like CD8^{POS} T cells. CD8^{POS} T cells were marked with a red dotted line.

(I, J, K) *Differentiating stemness- and effector-like CD8^{POS} T cells in CT-2A, GL261 and 005 murine GB*. scRNAseq analysis of CT-2A (CD45^{POS} TU) cells (dataset from Chen *et al.,*)[4], distinguishes NK cells from T cells based on *Klrb1c* and *Cd3b* expression, respectively. Then, helper and regulatory T cells, expressing *Cd4* and *Foxp3* genes, were discriminated from the CD8^{POS} T cells by marked *Cd8a/b* expression. In the CD8^{POS} T cell cluster, we observed naïve and stemness-like CD8^{POS} T cells, expressing *Tcf7* (encoding TCF-1) and *Nsg2* genes, that were different from the CD8^{POS} T cells with an effector-like phenotype expressing *Havcr2* (encoding TIM-3), *Pdcd1* (encoding for PD-1), *Gzmb* (encoding for cytotoxic granzyme-B), *Tnfrsf9* (encoding for 4-1BB), *Il12rb1, Il12rb2*, and *Stat4*. CD8a T cells are marked with a red dotted line.

(L) *Schematic overview to illustrate the stages of T cell differentiation*. Stemness-like CD8^{POS} T cells (TCF-1^{POS}) mature into effector-like CD8^{POS} T cells (TCF-1^{NEG}, GZM-B^{POS}) and express PD-1, TIM-3, 4-1BB, and IL-12 receptor, and finally become hypofunctional with marked CD101 expression.

(M) *Makers of differentiation, cytotoxicity, and hypofunctionality of CD8^{POS} T cells are increased upon rIL-12 treatment of GB.* Gene expression levels showed that *Pdcd1* (the PD-1 gene – average CT values 25.7 sham; 28.6 rIL-12), *Gzmb* (average CT values 23.3 sham; 24.9 rIL-12), and *Cd101* (average CT values 27.9 sham; 29.4 rIL-12), transcripts were increased with 5.6-, 3.0- and 2.9-fold, respectively in rIL-12 treated (blue) GB brains compared to Fc control (grey). Bar graphs represent transcript measurement from the total mouse brain at day 18 post tumor implantation, Data represent three independent experiments and are presented as the mean with SEM (error bars). Data were analyzed using paired student t-test, *$p < 0.05$, **$p < 0.01$.

(N) *PD-1^{POS} cells are more present in differentiated CD8^{POS} T cells at the tumor site.* Quantification of flow cytometry PD-1^{POS} and PD-1^{NEG} cells in different CD8^{POS} T cell subsets (TCF-1^{POS} (box 1 in Figure

4-C), TCF-1^{NEG}TIM-3^{NEG} (box 2 in Figure 4-C) and TIM-3^{POS} (box 3 in in Figure 4-C) comparing rIL-12 (blue) and sham control (grey). Within the TIM-3^{POS} population, a 4-fold higher number of PD-1^{POS} cells was observed compared to the TCF-1^{POS} subset and 2-fold increased PD-1 levels compared to TIM-3^{NEG}TCF-1^{NEG} cells (left). The opposite was observed for PD-1^{NEG} CD8^{POS} T cells (right). Data represent two independent experiments and are presented as the mean with SEM (error bars). Data were analyzed using two-way ANOVA, ****$p < 0.0001$.

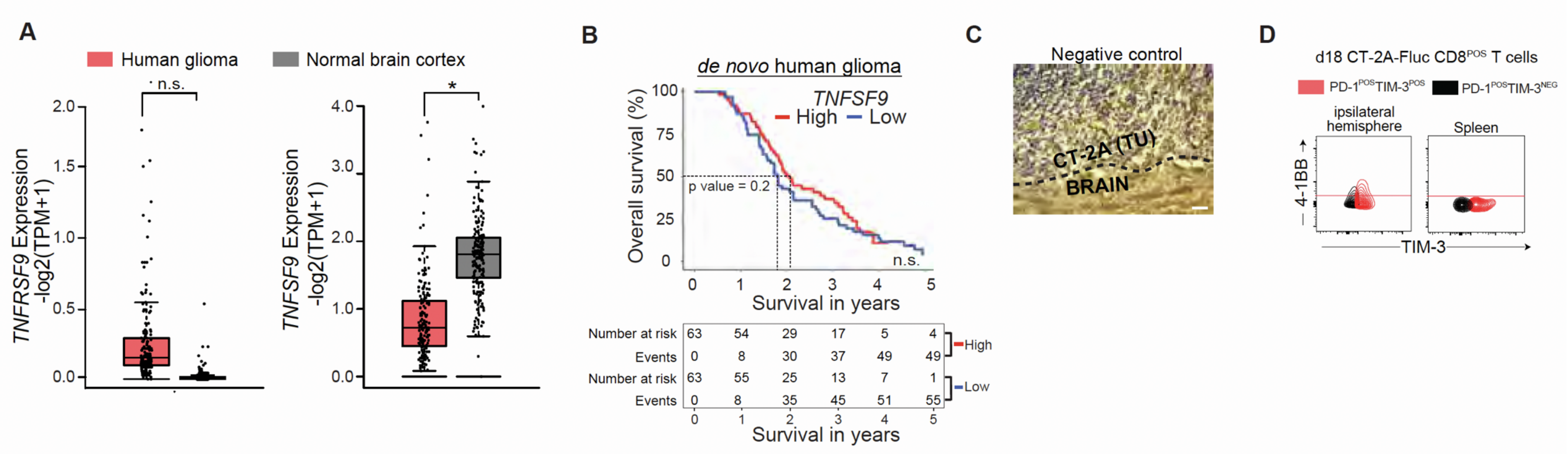
A
Human glioma
Normal brain cortex
n.s.
*
TNFRSF9 Expression -log2(TPM+1)
TNFSF9 Expression -log2(TPM+1)
B
de novo human glioma
TNFSF9
High
Low
Overall survival (%)
p value = 0.2
n.s.
Survival in years
Number at risk 63 54 29 17 5 4
Events 0 8 30 37 49 49
Number at risk 63 55 25 13 7 1
Events 0 8 35 45 51 55
C
Negative control
CT-2A (TU)
BRAIN
D
d18 CT-2A-Fluc CD8POS T cells
PD-1POSTIM-3POS
PD-1POSTIM-3NEG
ipsilateral hemisphere
Spleen
4-1BB
TIM-3

Figure S6. Expression and relevance of TNFSF9 (4-1BBL) in glioma and tumor infiltrating CD8 T cells.

(A) *Expression of TNFRSF9 and TNFSF9 in glioma patients.* Box plots showing the expression of *TNFRSF9* (encoding for 4-1BB) and *TNFSF9* (encoding for 4-1BBL) gene in GB patients (n = 163) compared to tissue of normal brain cortex (n = 207). The plot showed significant low expression of *TNFSF9* in glioma tissue as compared to normal brain. The data was generated from GEPIA 2.0 (http://gepia2.cancer-pku.cn/#index) [9] using the TCGA portal. *$p < 0.05$, not significant (n.s.).

(B) *Survival probability of de novo human glioma differentiating high and low expressing TNFSF9.* Kaplan-Meier survival curves based on Miller *et al.*,[2] showing the overall survival outcomes over a period of 5 years of a total of 63 glioma patients (IDH-WT) per group with high (red) or low (low) levels of *TNFSF9* (encoding for 4-1BBL), both groups had a median of ~2 years [2]. No differences were observed between groups. Log-rank (Mantel-Cox) test, p-value > 0.9, not significant (n.s). Median survival in days (MS). (C) *Primary antibody (4-1BB) control staining in brain tissues implanted with a CT-2A tumor.* Immuno-histochemistry lacking primary antibody (4-1BB) in the CT-2A tumor (TU) cells in the brain TME (mag-nification 40x; scale bar = 100 μm).

(D) *Representative flow cytometry counter plots.* Overlaid counter plots of 4-1BB expression within the PD-1^{POS}TIM-3^{POS} (effector-like $CD8^{POS}$ T cells) and PD-1^{POS}TIM-3^{NEG} (other $CD8^{POS}$ T cells) populations showed the increased presence of 4-1BB receptor in the ipsilateral hemisphere compared to the spleen.

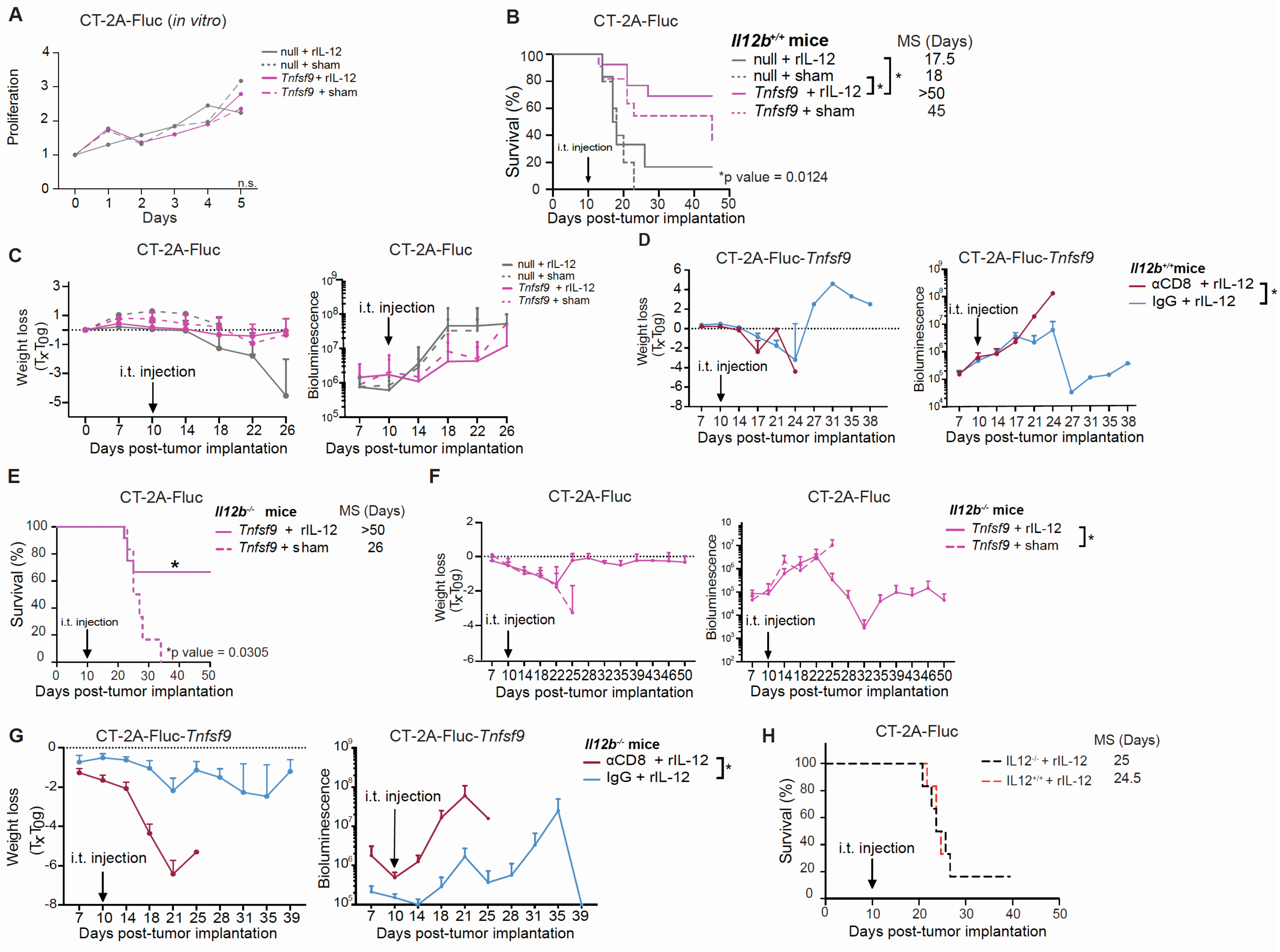

A
CT-2A-Fluc (in vitro)
Proliferation
Days
null + rIL-12
null + sham
Tnfsf9 + rIL-12
Tnfsf9 + sham
n.s.
B
CT-2A-Fluc
Il12b+/+ mice
MS (Days)
null + rIL-12 17.5
null + sham 18
Tnfsf9 + rIL-12 >50
Tnfsf9 + sham 45
Survival (%)
i.t. injection
*p value = 0.0124
Days post-tumor implantation
C
CT-2A-Fluc
Weight loss (Tx-T0g)
Bioluminescence
D
CT-2A-Fluc-Tnfsf9
Il12b+/+mice
αCD8 + rIL-12
IgG + rIL-12
E
Il12b-/- mice
Tnfsf9 + rIL-12 >50
Tnfsf9 + sham 26
*p value = 0.0305
F
G
H
IL12-/- + rIL-12 25
IL12+/+ + rIL-12 24.5

Figure S7. ***Tnfsf9*** **enhances rIL-12–mediated anti-tumor immunity in glioma.**

(A) *Cell viability assay.* CT-2A-FLuc-null and CT-2A-FLuc-*Tnfsf9* cell lines were exposed to 50 ng rIL-12 or sham control *in vitro* and cell viability (proliferation) was measured over a period of 5 days. Data represents triplicates and no significant differences were observed between groups, unpaired t-test, not significant (n.s.).

(B) *Survival benefit of local Tnfsf9 expression in CT-2-FLuc-bearing mice*. Kaplan-Meier curves showing survival outcomes following treatment of CT-2A-FLuc-null with sham (dashed grey) or CT-2A-FLuc-Tnfsf9 treated with sham (dashed pink) and CT-2A-FLuc-null with rIL-12 (solid grey) or CT-2A-FLuc-*Tnfsf9* treated with rIL-12 (solid pink) (n= 4-5 mice per group). Mice injected with CT-2A-FLuc-*Tnfsf9* tumor cells treated with rIL-12 (50 ng) had a median survival of >50 days, mice treated with sham control had a median survival of 45 days. Compared to mice implanted with tumor cells lacking *Tnfsf9* treated with rIL-12 or sham had a median survival of 17.5 or 18 days, respectively. Data represent at least two independent experiments and are presented as the mean with ± SEM (error bars). Data were analyzed using the Log-rank (Mantel-Cox) test, *p = 0.0124 (null + sham vs Tnfsf9 + rIL12 and null + rIL12 vs Tnfsf9 + rIL12). Median survival in days (MS).

(C) Weight (left) and tumor growth (right) (from panel B) were measured over time in $Il12^{+/+}$ mice injected with CT-2A-FLuc-null or CT-2A-FLuc-*Tnfsf9* tumor cells and treated with rIL-12 (solid pink) or sham (dashed pink). Mice treated with Fc control showed a weight drop at day 22, while rIL-12 treated mice maintained their weight over 50 days. Mice treated with rIL-12 showed a decrease in tumor size starting at day 22, with increasing size in Fc treated mice to day 28 - time of death (n=9-12 mice per group). Weight loss was normalized to each mouse its initial body weight and expressed in grams relative to the starting weight at treatment onset (Tx–T0).

(D) Weight (left) and tumor growth (right) were measured over time in tumor-bearing $Il12b^{+/+}$ mice injected with IgG and rIL-12 (solid blue) or anti-CD8 and rIL-12 (solid red). After T-cell depletion, weights of mice dropped at day 21 and these mice had significantly increased tumor sizes compared to IgG

control. (n=5-6 mice per group). Weight loss was normalized to each mouse its initial body weight and expressed in grams relative to the starting weight at treatment onset (Tx–T0).

(E) *GB mouse survival upon Tnfsf9 and rIL-12 combination treatment is not dependent on endogenous IL-12.* Kaplan-Meier curves of *Il12*$^{-/-}$ mice showing survival outcome of CT-2A-FLuc-*Tnfsf9* tumor-bearing mice injected i.t. with rIL-12 (solid pink), or the sham (dashed pink). Mice (n= 9-12 mice per genotype) treated with rIL-12 had a median survival of >50 days compared to 26 days for sham treated. Data represent at least two independent experiments and are presented as the mean with ± SEM (error bars). Data were analyzed using the Log-rank (Mantel-Cox) test, $^*p < 0.05$. Median survival in days (MS).

(F) Weight (left) and tumor growth (right) (from panel E) were measured over time in WT mice injected with CT-2A-FLuc-control (grey) or CT-2A-FLuc-Tnfsf9 (red) tumor cells comparing rIL-12 (solid) to sham control (dashed) treatment (n = 5-11 mice per group). Weight loss was normalized to each mouse its initial body weight and expressed in grams relative to the starting weight at treatment onset (Tx–T0).

(G) Weight (left) and tumor growth (right) were measured over time in tumor-bearing *Il12b*$^{-/-}$ mice injected with IgG and rIL-12 (solid blue) or anti-CD8 and rIL-12 (solid red). After T-cell depletion, weights of mice dropped at day 14 and these mice had significantly increased tumor sizes compared to IgG control. (n=5-6 mice per group). Data represents at least two independent experiments and were analyzed using multiple t-test, $^*p < 0.05$. Weight loss was normalized to each mouse its initial body weight and expressed in grams relative to the starting weight at treatment onset (Tx–T0).

(H) *Survival curves of GB-bearing mice in Il12b*$^{+/+}$ *and Il12b*$^{-/-}$ *mice treated with rIL-12.* Kaplan-Meier survival curves showing no overall survival benefit of CT-2A tumor-bearing *Il12*$^{+/+}$ mice (red) and *Il12*$^{-/-}$ mice (blue) (n = 5-6 mice per group) after rIL-12 treatment (median survival of 24.5 days and 25 days, respectively). Data represents at least two independent experiments. No differences were observed between the groups. Log-rank (Mantel-Cox) test, not significant (n.s). Median survival in days (MS).

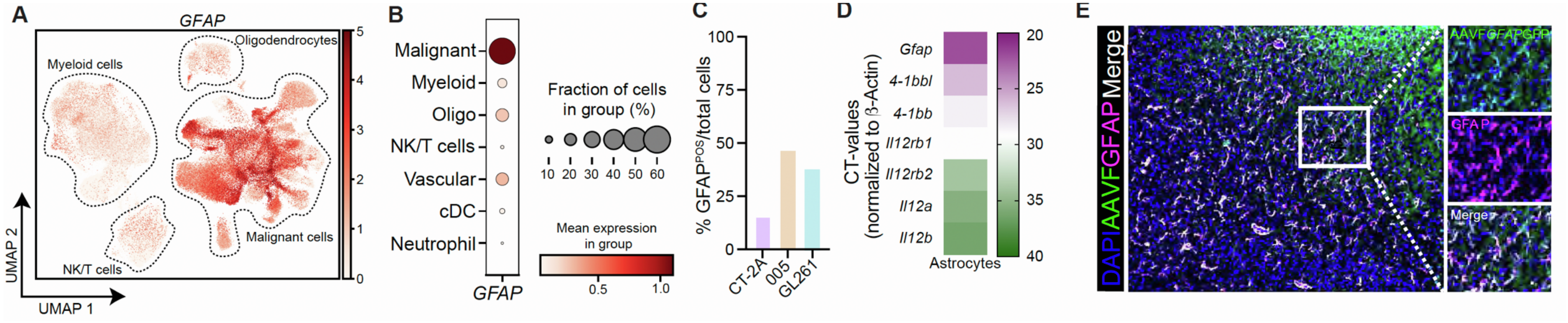
A
GFAP
Oligodendrocytes
Myeloid cells
Malignant cells
NK/T cells
UMAP 2
UMAP 1
5
4
3
2
1
0
B
Malignant
Myeloid
Oligo
NK/T cells
Vascular
cDC
Neutrophil
GFAP
Fraction of cells in group (%)
10 20 30 40 50 60
Mean expression in group
0.5
1.0
C
% GFAP^POS/total cells
100
75
50
25
0
CT-2A
005
GL261
D
CT-values (normalized to β-Actin)
Gfap
4-1bbl
4-1bb
Il12rb1
Il12rb2
Il12a
Il12b
Astrocytes
20
25
30
35
40
E
DAPIAAVFGFAPMerge
AAVFGFAPGFP
GFAP
Merge

Figure S8. Assessment of GFAP expression and cytokine receptor signatures in astrocytes within glioma.

(A) *UMAP projections of expression of GFAP in human glioma.* The scRNAseq dataset [2] of human glioma cells were analyzed. Distinct cell type subsets were clustered, annotated and visualized with a high-resolution color coded UMAP projection. *GFAP* was most expressed in the malignant cluster, compared to the myeloid, oligodendrocyte, DC, and NK/T cell clusters.

(B) *Expression of GFAP in human glioma.* Human *GFAP* (see panel A) was expressed at high levels in malignant cluster as depicted in a dot plot (dataset from Miller *et al*.,)[2].

(C) Quantification of the number of $GFAP^{POS}$ shown as a percentage of total cells as stained for by DAPI comparing CT-2A, 005 and GL61 GB models. Images of brain tumors in mice were analyzed by Image J (n=1 per group). (Dataset from Miller *et al*.,)[2].

(D) Gene expression levels shown for *Gfap*, *Il12a*, *Il12b*, *Il12rb1*, *Il12rb2 41bb* and *41bbl* mRNA measured in primary mouse astrocytes. Data are plotted as CT values normalized to β-actin and displayed as a heatmap.

(E) Immunofluorescence of a brain without tumor i.c. injected with AAVF-*GFAP*-GFP vector showed successful targeting of GFAP astrocytes after 14 days post-injection. (10x magnification, scale bar = 10 µm, left; 40x magnification, scale bar = 50 µm, right).

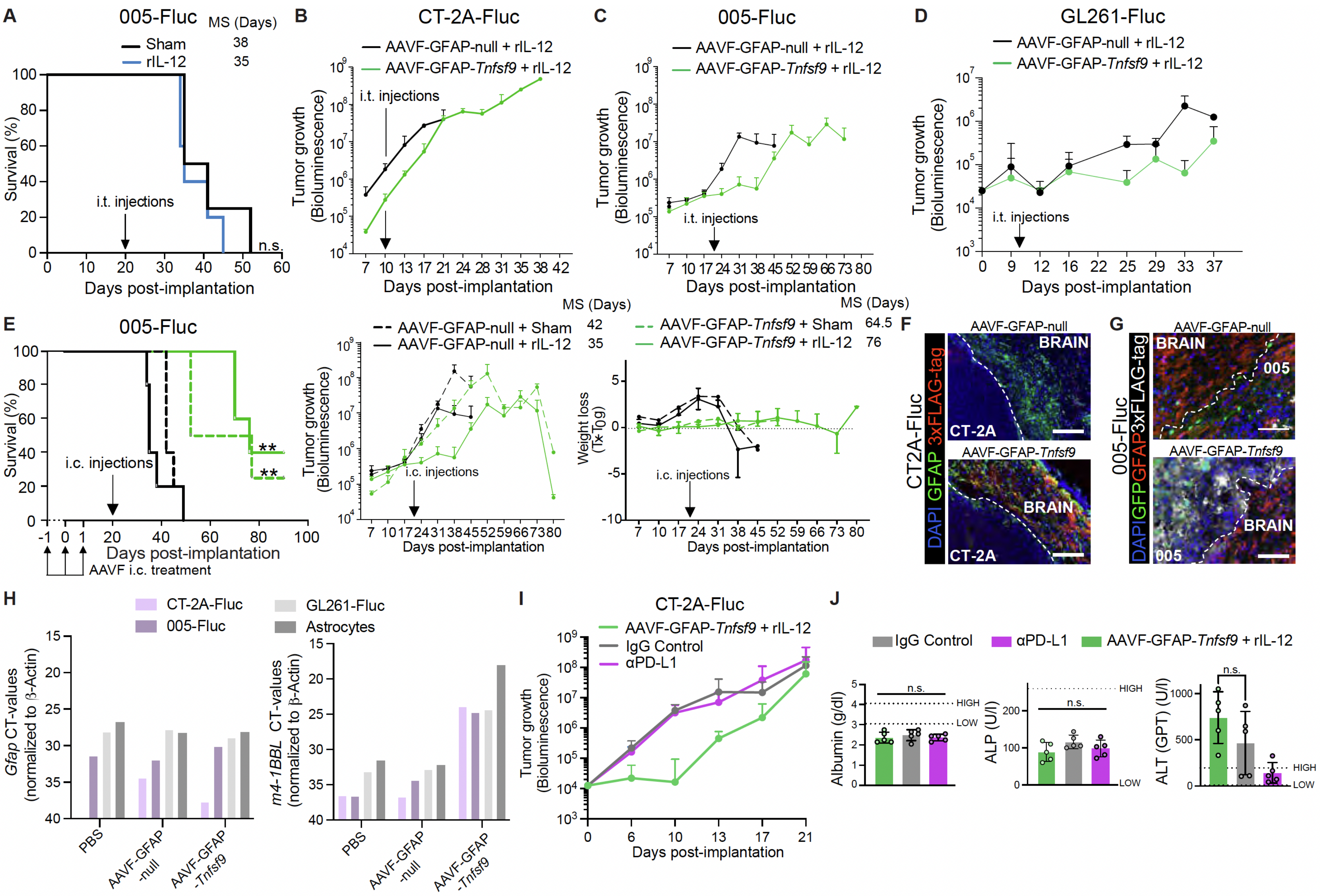

A
005-Fluc
MS (Days)
Sham 38
rIL-12 35
Survival (%)
i.t. injections
n.s.
Days post-implantation
B
CT-2A-Fluc
AAVF-GFAP-null + rIL-12
AAVF-GFAP-Tnfsf9 + rIL-12
Tumor growth (Bioluminescence)
i.t. injections
Days post-implantation
C
005-Fluc
AAVF-GFAP-null + rIL-12
AAVF-GFAP-Tnfsf9 + rIL-12
Tumor growth (Bioluminescence)
i.t. injections
Days post-implantation
D
GL261-Fluc
AAVF-GFAP-null + rIL-12
AAVF-GFAP-Tnfsf9 + rIL-12
Tumor growth (Bioluminescence)
i.t. injections
Days post-implantation
E
005-Fluc
MS (Days)
AAVF-GFAP-null + Sham 42
AAVF-GFAP-null + rIL-12 35
AAVF-GFAP-Tnfsf9 + Sham 64.5
AAVF-GFAP-Tnfsf9 + rIL-12 76
Survival (%)
i.c. injections
**
**
Days post-implantation
AAVF i.c. treatment
Tumor growth (Bioluminescence)
i.c. injections
Days post-implantation
Weight loss (T*T0g)
i.c. injections
Days post-implantation
F
CT2A-Fluc
DAPI GFAP 3xFLAG-tag
AAVF-GFAP-null
BRAIN
CT-2A
AAVF-GFAP-Tnfsf9
BRAIN
CT-2A
G
005-Fluc
DAPI GFP GFAP 3xFLAG-tag
AAVF-GFAP-null
BRAIN
005
AAVF-GFAP-Tnfsf9
BRAIN
005
H
CT-2A-Fluc
005-Fluc
GL261-Fluc
Astrocytes
Gfap CT-values (normalized to β-Actin)
m4-1BBL CT-values (normalized to β-Actin)
PBS
AAVF-GFAP -null
AAVF-GFAP -Tnfsf9
I
CT-2A-Fluc
AAVF-GFAP-Tnfsf9 + rIL-12
IgG Control
αPD-L1
Tumor growth (Bioluminescence)
Days post-implantation
J
IgG Control
αPD-L1
AAVF-GFAP-Tnfsf9 + rIL-12
Albumin (g/dl)
ALP (U/l)
ALT (GPT) (U/l)
n.s.
HIGH
LOW

Figure S9. Astrocyte-directed *Tnfsf9* expression enhances rIL-12–mediated glioma control without systemic toxicity.

(A) *Therapeutic survival benefit is reduced by delayed treatment of 005-FLuc-bearing mice with rIL-12.* Kaplan-Meier curves display the survival percentage of 005-FLuc-bearing mice (100,000 cells at the time of injection) with treatment at day 20 post-tumor implantation, comparing i.c. injection of 50 ng rIL-12 (blue) to sham control (black) (n=4-5 mice per group). Log-rank (Mantel-Cox) test, no significant difference was observed for rIL-12-treated mice with a median survival of 35 days compared to sham with a median survival of 38 days. Median survival in days (MS); not significant (n.s.).

(B) Average bioluminescence levels (representing tumor size) of CT-2A-FLuc tumor-bearing mice were measured over time, comparing AAVF-GFAP-null treated with rIL-12; (solid black) and AAVF-GFAP-*Tnfsf9* treated with rIL-12 (solid green) (n=4-6 mice per group).

(C) Average bioluminescence levels of 005-FLuc tumor-bearing mice were measured over time comparing AAVF-GFAP-null treated with rIL-12; (solid black) and AAVF-GFAP-*Tnfsf9* treated with rIL-12 (solid green) (n=4-5 mice per group).

(D) Average bioluminescence levels (representing tumor size) of GL261-FLuc tumor-bearing mice were measured over time, comparing AAVF-GFAP-null treated with rIL-12; (solid black) and AAVF-GFAP-*Tnfsf9* treated with rIL-12 (solid green) (n=4-5 mice per group).

(E) *Intratumoral rIL-12 administration combined with AAVF-mediated Tnfsf9 expression improves survival in 005 glioma-bearing mice*. Kaplan-Meier curve (left), weight loss (middle), and tumor growth (right) graphs of mice i.c. implanted with 005-FLuc glioma cells (50,000 cells) and treated with AAVF-GFAP vectors. Mice received three i.c. injections of either AAVF-GFAP-*Tnfsf9* or AAVF-GFAP-null vector on days –1, 0, and +1, followed by either rIL-12 or sham treatment on day 20 post-tumor implantation. The AAVF-GFAP-*Tnfsf9* treated with rIL-12 group (solid green) showed prolonged median survival (76 days) and delayed tumor progression compared to all other groups. The AAVF-GFAP-*Tnfsf9* treated with sham group (dashed green) showed modest survival benefit, whereas AAVF-GFAP-null mice receiving either rIL-12 (solid black) or sham (dashed black) showed accelerated tumor growth and

reduced survival (35 and 42 days, respectively). Body weight remained relatively stable in the double-treated group, correlating with tumor control. These findings highlight the importance of both early co-stimulation (*Tnfsf9*) and cytokine activation (rIL-12) in promoting durable anti-tumor responses in the 005-glioma model. n = 5-6 mice/group; log-rank (Mantel-Cox) test, **$p < 0.01$. Median survival in days (MS).

(F, G) *Immunofluorescence of brain sections from mice implanted with CT-2A-Fluc tumor (12,500 cells) or 005-Fluc tumor (50,000 cells) i.t. injected with AAVF-GFAP null and Tnfsf9 vector (three times)*. Images show successful targeting of GFAP astrocytes and 3x FLAG-tag (red) in TME in the tumor vicinity at 18 days in CT-2A tumor and at 28 days in 005 tumor post implantation. The white dotted line represents the tumor border. (40x magnification, scale bar = 50 μm).

(H) *AAV vectors can transduce both murine GB cell lines and primary astrocytes.* CT-2A cells (light purple), 005 cells (dark purple), GL261 (light grey), and primary derived astrocytes (grey) were trans-duced with AAVF-GFAP-*Tnfsf9* or AAVF-GFAP-null control and maintained for 7 days in culture. mRNA levels showed increased *Gfap* expression in astrocytes compared to CT-2A and 005 cells; all four cell types showed increased levels of the *Tnfsf9* transgene only after incubating with AAVF-GFAP-*Tnfsf9*, and not with the AAVF-GFAP-null control, compared to PBS control. Astrocytes expressed the highest levels of both *Gfap* and *Tnfsf9*. Data are plotted as CT values normalized to β-actin. Data represents one independent experiment.

(I) Average bioluminescence levels (representing the tumor size) of CT-2A-FLuc tumor-bearing mice were measured over time comparing AAVF-GFAP-*Tnfsf9* treated with rIL-12; (solid green); anti-PD-L1 not treated with rIL-12 (solid blue); or IgG control not treated with rIL-12 (solid grey) (n = 4-5 mice per group). Multiple t-test, not significant (n.s.).

(J) *Toxicity analysis post-therapy.* The blood of mice (n=5 per group) that received AAVF-GFAP-*Tnfsf9* and rIL-12 (green), αPD-L1 (blue), or IgG control (grey) was tested for toxicity markers systemically, including Albumin, ALP and ALT. No significance differences were observed. Blood was collected retro-orbitally on day 15 post-tumor implantation. Data represent one independent experiment and are

presented as the mean with ± SEM (error bars). Data were analyzed using one way ANOVA, not significant (n.s.).

References

1. Mathewson ND, Ashenberg O, Tirosh I, et al. Inhibitory CD161 receptor identified in glioma-infiltrating T cells by single-cell analysis. *Cell.* 2021;184(5):1281-1298 e1226.
2. Miller TE, El Farran CA, Couturier CP, et al. Programs, origins and immunomodulatory functions of myeloid cells in glioma. *Nature.* 2025;640(8060):1072-1082.
3. Pombo Antunes AR, Scheyltjens I, Lodi F, et al. Single-cell profiling of myeloid cells in glioblastoma across species and disease stage reveals macrophage competition and specialization. *Nat Neurosci.* 2021;24(4):595-610.
4. Tomaszewski WH, Waibl-Polania J, Chakraborty M, et al. Neuronal CaMKK2 promotes immunosuppression and checkpoint blockade resistance in glioblastoma. *Nat Commun.* 2022;13(1):6483.
5. Chen D, Varanasi SK, Hara T, et al. CTLA-4 blockade induces a microglia-Th1 cell partnership that stimulates microglia phagocytosis and anti-tumor function in glioblastoma. *Immunity.* 2023;56(9):2086-2104 e2088.
6. Haddad AF, Young JS, Amara D, et al. Mouse models of glioblastoma for the evaluation of novel therapeutic strategies. *Neurooncol Adv.* 2021;3(1):vdab100.
7. Khalsa JK, Cheng N, Keegan J, et al. Immune phenotyping of diverse syngeneic murine brain tumors identifies immunologically distinct types. *Nat Commun.* 2020;11(1):3912.
8. Marumoto T, Tashiro A, Friedmann-Morvinski D, et al. Development of a novel mouse glioma model using lentiviral vectors. *Nat Med.* 2009;15(1):110-116.
9. Tang Z, Kang B, Li C, Chen T, Zhang Z. GEPIA2: an enhanced web server for large-scale expression profiling and interactive analysis. *Nucleic Acids Res.* 2019;47(W1):W556-W560.
